# Natural Product-Oriented Photo-Induced Denitrogenative Annulations of 1-Alkenylbenzotriazoles

**DOI:** 10.3390/molecules28010363

**Published:** 2023-01-02

**Authors:** Zhiguo Wang, Yi Chen, Zhen Dong, Yefeng Tang

**Affiliations:** 1 School of Pharmaceutical Sciences, MOE Key Laboratory of Bioorganic Phosphorus Chemistry & Chemical Biology, Tsinghua University, Beijing 100084, China; 2 College of Chemical Engineering, Qinghai University, Xining 810016, China

**Keywords:** indole alkaloid, 1-alkenylbenzotriazole, photochemical transformation, denitrogenative annulation, retro-Fries rearrangement

## Abstract

The photo-induced denitrogenative annulations of a variety of 1-alkenylbenzotriazoles were investigated. By judiciously manipulating the structural variations of 1-alkenylbenzotriazoles, two characteristic polycyclic skeletons associated with monoterpene indole alkaloids were constructed through a diverted and controllable manner. The present work not only enriches the photochemistry of 1-alkenylbenzotriazoles, but also offers a unified approach to access skeletally diverse indole alkaloid scaffolds.

## 1. Introduction

**Figure 1:**
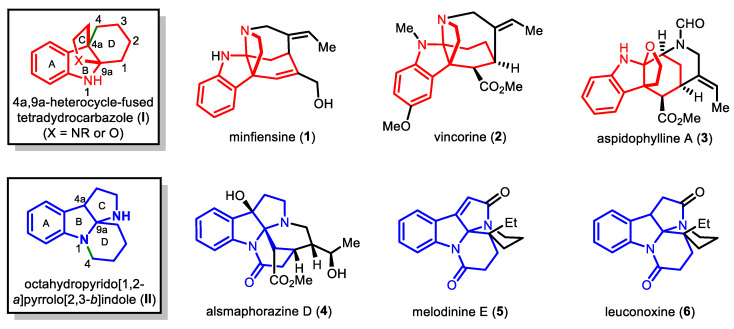
Representative indole alkaloids containing the tetracyclic skeleton **I** and **II**.

Monoterpene indole alkaloids comprise a large class of natural products that exhibit diverse molecular architectures and broad biological activities [1,2]. Among them, an array of *strychnos* and akuammiline alkaloids featuring a 4a,9a-heterocycle-fused tetrahydrocarbazole skeleton (**I**, Figure 1), as shown in minfiensine (**1**), vincorine (**2**) and aspidophylline A (**3**), have spurred great interest of synthetic chemists in the past decades. Characterized by a unique [4.3.3]propellane core containing two adjacent quaternary stereocenters, tetracyclic skeleton **I** has been viewed as one of the major synthetic challenges associated with this group of natural products, and thus a plethora of synthetic approaches have been developed to access it and related scaffolds [3,4,5]. Besides the above-mentioned molecules, there also exist some monoterpene indole alkaloids that bear the same A/B/C tricyclic ring system to **I** but differ in the connectivity patterns between the A/B/C and D rings [1]. Taking alsmaphorazine D (**4**), melodinine E (**5**) and leuconoxine (**6**) as examples, all of them share a characteristic octahydropyrido[1,2-a]pyrrolo[2,3-b]indole core (**II**), in which the D ring is connected to A/B/C ring at N1 and C9a instead of C4a and C9a as shown in **I**. The structural similarity between **I** and **II** could be rationalized by their inherent biosynthetic relationship. It has been suggested that the latter should be biosynthetically derived from the former through the cleavage of the C4a–C4 bond followed by the formation of a N1–C4 bond [6,7]. Of note, although this biosynthetic hypothesis seems to be inspiring, it has remained yet to be validated in practice [8].

**Scheme 1:**
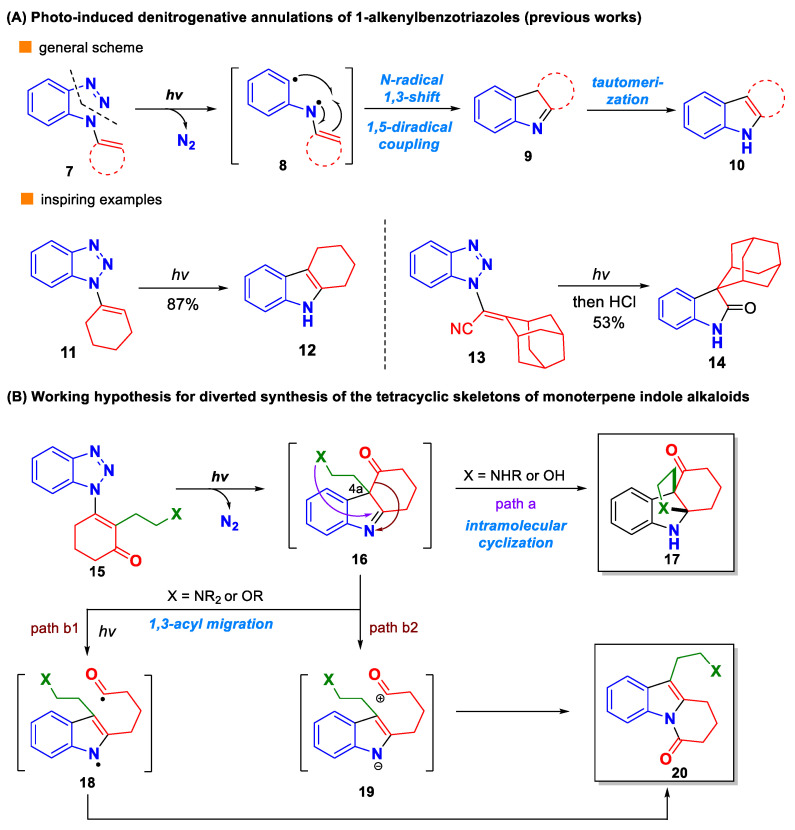
Research background and working hypothesis (**A**,**B**).

1,2,3-Benzotriazoles are an important class of heterocycles that have found widespread applications in organic synthesis, medicinal chemistry, and material science [9,10,11,12,13]. Historically, it has been reported that some 1-substituted 1,2,3-benzotriazoles could undergo denitrogenative transformations to generate other valuable products. This unique reactivity has aroused considerable interest from the synthetic community in recent years, which leads to the development of a broad range of novel denitrogenative transformations of 1,2,3-benzotriazoles [14,15,16,17,18,19]. Among them, some photochemical reactions are particularly attractive due to their appealing synthetic potential and environmentally benign nature [20,21,22,23,24,25,26,27]. For example, it has been reported that 1-vinyl-1,2,3-benzotriazole **7** could undergo sequential photo-induced nitrogen extrusion, N-radical 1,3-shift, 1,5-diradical combination and tautomerization to yield the indole product **10** (Scheme 1A). From a synthetic point of view, this unique photo transformation represents a promising synthetic tool to access indole-derived natural products. However, such potential has rarely been explored, with only sporadic cases documented [22,23]. In 1980s, Wender and co-workers reported a seminal work on this subject, in which a series of natural product-relevant indole scaffolds were prepared by this method, as exemplified by the case leading to tetrahydrocarbazole **12** [22]. Another notable example was reported by Johnson and co-workers, in which a spiro-oxindole derivative **14** was obtained through the photo-induced denitrogenative annulation of **13** followed by hydrolysis of the in situ-generated indolenine intermediate [23].

In recent years, our group has been striving to develop conceptually novel and practically useful denitrogenative transformations of 1,2,3-benzotriazoles [28,29,30]. During this course, we realized that the photo-induced denitrogenative annulation of 1-alkenylbenzotriazoles, if combined with rational design, might serve as an enabling tool for the synthesis of monoterpene indole alkaloids. As depicted in Scheme 1B, we envisioned that a functionalized 1-alkenylbenzotriazole like **15** could undergo the photo-induced denitrogenative annulation to give 2,3,3-trisubstituted indolenine intermediate **16**. Once formed, the imine moiety of **16** could be captured by a nitrogen- or oxygen-derived nucleophile (e.g., NHR or OH) pre-installed on the side chain at C4a (path a), thus affording the 4a,9a-heterocycle-fused tetrahydrocarbazole derivative **17**. On the other hand, if the nitrogen- or oxygen-derived nucleophile exists in a masked form (e.g., NR_2_, or OR), a different reaction pathway could be imagined for the intermediate **16**. Based on some inspiring cases [8], 1,3-acyl migration could take place, giving rise to the dihydropyrido[1,2-a]indolone derivative **20**, either through the diradical intermediate **18** (retro-photo-Fries rearrangement, path b1) [31,32,33,34,35] or zwitterion species **19** (path b2) [36,37,38,39]. Finally, **20** could be elaborated into the tetracyclic skeleton **II** through some additional operations. From a synthetic perspective, the above-mentioned synthetic blueprint appears to be attractive, since it enables the facile access of two different polycyclic skeletons associated with monoterpene indole alkaloids in a diverted and controllable manner.

## 2. Results and Discussion

At the initial of our study, two 1-alkenylbenzotrizoles **15a** and **15b**, which bear a NHBoc and OH moiety on the side chain, respectively, were prepared through a couple of steps (for details, see Supplementary Materials). With these precursors in hand, we then focused our attention on exploring the designed photo-induced denitrogenative annulation reaction. Based on some relevant works [17,18,19,20,21,22], we first chose to conduct the photoreaction of **15a** with MeCN as the solvent and 254 nm UV lamp as the light resource. To our disappointment, the reaction outcomes turned out to be complicated, and no desired product **17a** could be identified in the reaction mixtures (Table 1, entry 1). In sharp comparison, the photoreaction of **15b** appeared to be more encouraging, with the expected product **17b** obtained in 23% yield (entry 2). Besides, we also identified another unstable product (ca. 30%) in the reaction, which was assigned to be the 5/3/5 tricyclic compound **21** based on the extensive spectroscopic studies. Furtherly, we conducted a condition screening using **15b** as the substrate. However, only a slightly improved yield of **17b** (30%) was obtained when the reaction was performed in THF (entry 3).

**Scheme 2:**
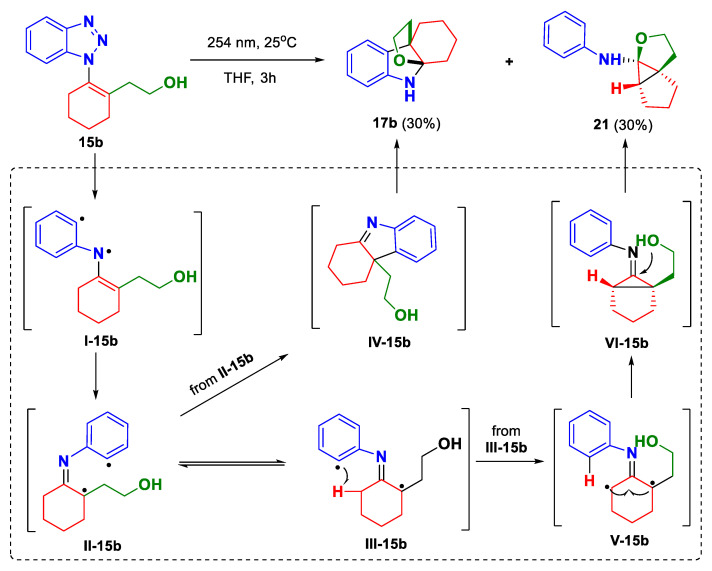
Initial results of the photo-induced denitrogenative annulation.

The above-mentioned results suggest that although the designed photo-induced denitrogenative annulation is feasible, the reaction outcomes largely rely on the judicious manipulation of the substrate structures. The distinct results associated with **15a** and **15b** indicate that a nucleophile with favorable steric and electronic nature should be employed to capture the in situ-generated indolenine intermediate. Apparently, the OH group appears to be a better choice than the NHBoc for the current reaction. On the other hand, the identification of byproduct **21** indicated that there exist some unexpected reaction pathways besides the desired one. The plausible mechanism of the photoreaction of **15b** is outlined in Scheme 2. Simply, upon photo irradiation, **15b** will undergo nitrogen extrusion to generate diradical species **I-15b.** Subsequently, **I-15b** will go through radical 1,3-shift to give 1,5-diradicals **II-15b** and **III-15b** as a pair of cis/trans isomers. Between them, the cis-isomer **II-15b** could advance to **17b** though 1,5-diradical combination followed by imine capture by the hydroxyl group. Comparably, for trans-isomer **III-15b**, a 1,5-hydrogen abstraction will take place preferentially, leading to a new 1,3-diradical species (**V-15b)** [40]. Finally, a 1,3-diradical combination followed by the cyclization of the hydroxyl group onto the imine moiety will give rise to the observed byproduct **21**.

**Scheme 3:**
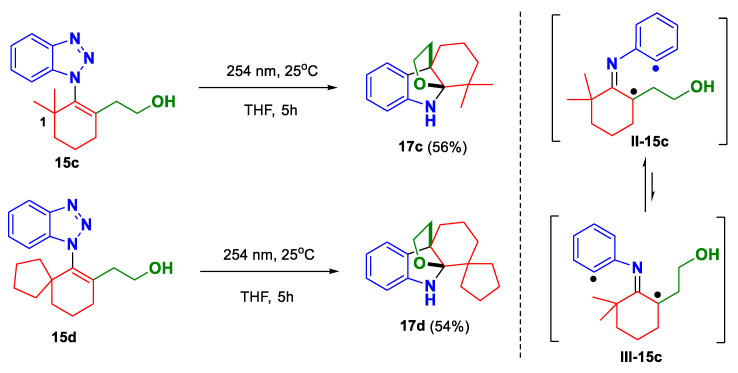
Photo-induced denitrogenative annulation of 1-alkenylbenzotrizoles **15c** and **15d**.

Having the mechanistic rationalization in mind, we sought to improve the reaction by judiciously manipulating the substrate structure. As we envisioned, introducing two substitutions on the C1 position of the substrate (e.g., **15c** and **15d** in Scheme 3) will facilitate the reaction from two aspects. First, the competitive 1,5-hydrogen abstraction would be precluded in this scenario due to the absence of suitable C–H bonds on the C1 position. Second, the equilibrium between the *cis*- and *trans*-imine intermediates will shift towards the desired direction. Taking **15c** as an example, **II-15c** should be thermodynamically more favorable than **III-15c**, and thus the desired reaction pathway would take place preferentially. To our delight, this speculation was validated quickly in practice. As shown in Scheme 3, when 1-alkenylbenzotrizoles **15c** and **15d** were submitted to the photoreaction, the corresponding annulation products **17c** (56%) and **17d** (54%) were obtained in notably improved yields.

**Scheme 4:**
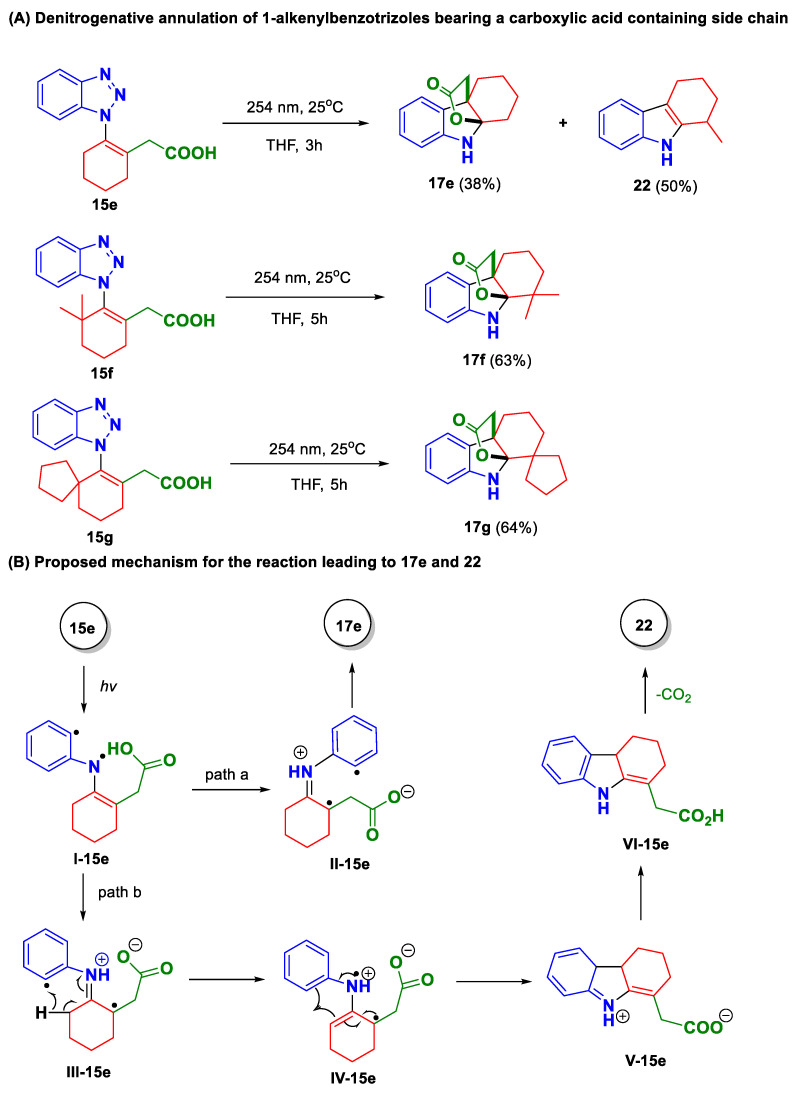
Photo-induced denitrogenative annulation of 1-alkenylbenzotrizole bearing a carboxylic acid-containing side chain (**A**,**B**).

Besides the C1 substitute effect, we found that the structural variation on the side chain also exerted a profound effect on the reaction outcome. Indeed, when 1-alkenylbenzotrizole **15e** bearing a carboxylic acid on side chain was attempted in the photoreaction, a different result was obtained. In this case, two products were isolated, between which the minor component turned out to be the desired product **17e** (38%) and the major one was assigned as tetrahydrocarbazole **22** (Scheme 4A). The structure of **22** was confirmed by the comparison of its spectroscopic data with those reported in literature [41]. Although the exact mechanism for the formation of **22** remains unclear at this stage, a plausible one is suggested in Scheme 4B. Naturally, the reaction also starts from the denitrogenation of **15e**. However, different from the above-mentioned cases, in the current scenario the resulting **I-15e** more likely advances to the zwitterion species **II-15e** and **III-15e** through N-radical 1,3-shift and intramolecular proton transfer from the carboxylic acid to the imine moiety. Between them, **II-15e** could convert to **17e** following the same pathway as suggested above. Comparably, **III-15e** might go through an intramolecular 1,5-hydrogen transfer followed by radical 1,3-shift to yield N-radical cation species **IV-15e**. Subsequently, a Nazarov-type cyclization would take place, and the resulting product **V-15e** could undergo sequential tautomerization and decarboxylation to yield byproduct **22**.

Taking the above outcomes into consideration, we decided to integrate the structural modifications on the C-1 position and C-4a side chain into a single molecule. To this end, 1-alkenylbenzotrizoles **15f** and **15g** were synthetized. As we expected, both **15f** and **15g** proved to be excellent substrates for the photoreaction by providing the corresponding products in good yields (**17f**: 63%; **17g**: 64%).

Having the 4a,9a-heterocycle-fused tetrahydrocarbazole skeleton (**I**) secured in an acceptable efficiency, we then moved to explore the proposed chemistry leading to the tetracyclic skeleton **II** (Figure 1). According to our design, two structural modifications should be implemented on the 1-alkenylbenzotrizoles. First, the nucleophile on the side chain should be masked, and thus the in situ-generated indolenine intermediate (e.g., **16**, Scheme 1) could not be captured. Secondly, a carbonyl group should be introduced onto the C-4 position of the cyclohexene unit, which allows the proposed 1,3-acyl migration to take place. Taking these rationalizations in mind, 1-alkenylbenzotrizoles **15h** and **15i** were prepared and evaluated in the photoreactions. To our delight, the designed chemistry worked well under the conventional photo conditions (254 nm UV, CH_3_CN, 25 ℃), with the desired dihydropyrido[1,2-a]indolone derivatives **20h** and **20i** obtained in 42% and 35% yields, respectively. Although the yields of the above reactions appeared to be moderated, their overall efficiency is appreciable, since they actually integrate several transformations into a single operation.

**Scheme 5:**
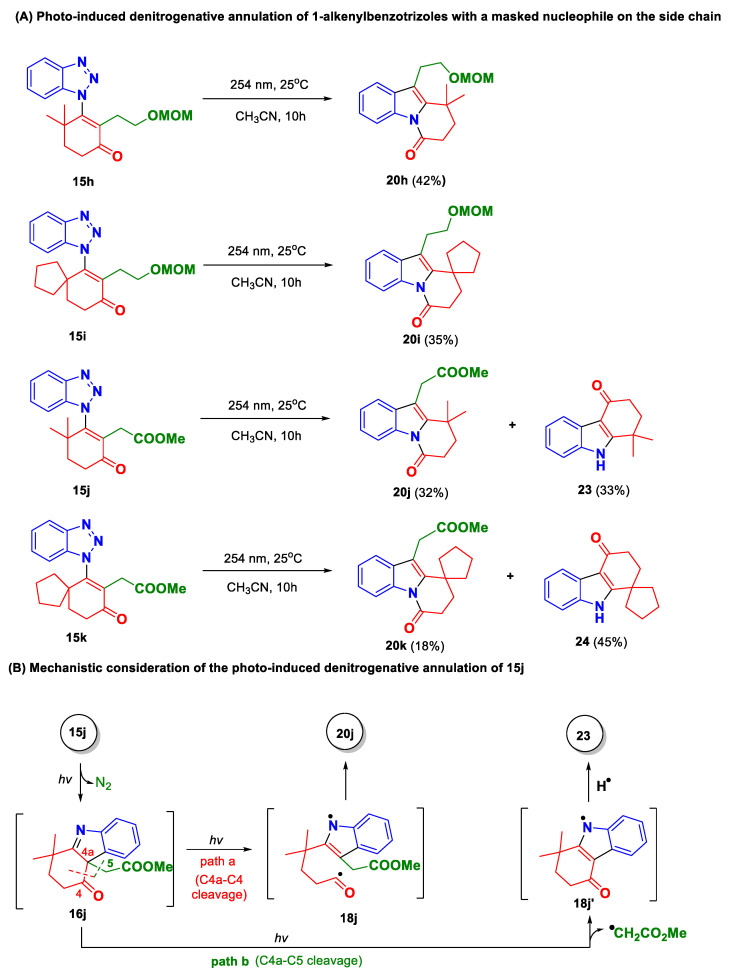
Photo-induced denitrogenative annulation of 1-alkenylbenzotrizoles with a masked nucleophile on the side chain (**A**,**B**).

Naturally, we also attempted the carboxylate-derived 1-alkenylbenzotrizoles **15j** and **15k** in the photoreaction. Again, these two substrates showed slightly different reactivity from **15h** and **15i**. As shown, besides the expected products **20j** and **20k**, we also identified another product in these reactions, which were assigned to be the tricyclic compounds **23** and **24**, respectively. Interestingly, compared with **20j** and **20k**, **23** and **24** not only showed different tricyclic ring systems, but also lost a unit of -CH_2_CO_2_Me. A plausible mechanism for the above-mentioned transformations is suggested in Scheme 5B. Taking **15j** as example, a photo-induced denitrogenative annulation will take place first, leading to indolenine intermediate **16j**. Without a suitable nucleophile to trap the imine moiety, **16j** would go through two different pathways upon further photo irradiation. On one hand, the proposed retro-photo-Fries rearrangement will take place through the cleavage of C4a-C4 bond (path a), and the resulting diradical species **18j** readily converts to the product **20j** through 1,6-radical combination. On the other hand, the cleavage of C4a-C5 bond might also occur as a competitive pathway (path b), with the release of an α-carbonyl radical species. Subsequently, the resulting **18j’** will abstract a hydrogen form the reaction system to yield the byproduct **23**. Interestingly, we did not observe similar byproducts in the reactions with **15h** and **15i**, indicating that the generation of a stabilized α-carbonyl radical species should be the driving force of path b.

## 3. Materials and Methods

### 3.1. General Information

Commercially available reagents were purchased from commercial sources and used as received without further purification. If no further details are given, the reaction was performed under ambient atmosphere and temperature. Analytical thin layer chromatography (TLC) was performed on silica gel-coated plates (Merck, 60 F254) with the indicated solvent mixture, and visualization was performed using ultraviolet (UV) irradiation (λ = 254 nm) and/or staining with aqueous KMnO_4_. If not specially mentioned, flash column chromatography used silica gel (200–300 mesh) supplied by Tsingtao Haiyang Chemicals (Qingdao, China).

^1^H NMR spectra were recorded on a Bruker Avance III 400 (400 MHz) spectrometer. TMS (δH 0.00) were used as the internal reference. ^13^C NMR spectra were recorded on a Bruker Advance III 400 (100 MHz) spectrometer in CDCl_3_ (δC 77.16) using their central resonance as the internal reference. All ^13^C NMR spectra were proton decoupled. High-resolution mass spectra (HRMS) were recorded on a Waters Xevo G2 QTOF MS. A commercially available UV lamp (model: Philips TUV 25W/G25 T8, emission wave-length range: 200–280 nm; λmax: 254 nm) was used as light resource.

### 3.2. General Procedure

A quartz tube was charged with 1-alkenylbenzotriazoles (40 mg) at N_2_ atmosphere. The freshly distilled THF or MeCN (5.0 mL) was added and the reaction mixture was stirred at room temperature for 3–10 h upon photolysis (254 nm). The resulting solution was concentrated in vacuo. The residue was directly purified by flash chromatography (silica gel, 200–300 mesh, hexanes/EtOAc or CH_2_Cl_2_/MeOH) to yield the corresponding product (Irradiation system, see Supplementary Materials).

2-(2-(1H-Benzo[d][1,2,3]triazol-1-yl)cyclohex-1-en-1-yl) ethanol (**15b**): colorless oil; Yield: 86%; ^1^H NMR (400 MHz, CDCl_3_) δ 1.78–1.93 (m, 4H), 1.99 (t, *J* = 6.5 Hz, 2H), 2.33–2.39 (m, 2H), 2.41–2.47 (m, 2H), 2.53 (t, *J* = 5.3 Hz, 1H), 3.59–3.65 (m, 2H), 7.35 (ddd, *J* = 8.1, 5.9, 1.9 Hz, 1H), 7.42–7.51 (m, 2H), 8.02 (dd, *J* = 8.4, 1.0 Hz, 1H). ^13^C NMR (100 MHz, CDCl_3_) δ 145.6, 136.6, 132.9, 130.0, 127.8, 124.1, 120.1, 110.3, 60.0, 35.5, 29.6, 28.8, 22.9, 22.2. HRMS m/z calcd for C_14_H_18_N_3_O [M+H]^+^: 244.1450; found: 244.1449.

(4bR,8aR)-5,6,7,8-Tetrahydro-9H-8a,4b-(epoxyethano)carbazole (**17b**): white solid; Yield: 30%; ^1^H NMR (400 MHz, CDCl_3_) δ 1.35–1.47 (m, 3H), 1.60–1.68 (m, 2H), 1.70–1.80 (m, 1H), 1.92–1.99 (m, 2H), 2.17–2.25 (m, 2H), 3.57–3.67 (m, 1H), 3.89–3.96 (m, 1H), 4.30 (s, 1H), 6.61 (m, 1H), 6.76 (t, *J* = 7.4 Hz, 1H), 7.02–7.08 (m, 2H).^13^C NMR (100 MHz, CDCl_3_) δ 148.3, 135.6, 127.8, 122.8, 119.2, 109.2, 102.4, 66.2, 53.5, 38.0, 33.1, 32.4, 20.8, 19.7. HRMS m/z calcd for C_14_H_18_NO [M+H]^+^: 216.1388; found: 216.1387.

N-Phenylhexahydro-3aH-cyclopenta[2,3]cyclopropa[1,2-b]furan-3a-amine (**21**): white solid; Yield: ca. 30%; ^1^H NMR (400 MHz, CDCl_3_) δ 1.49–1.64 (m, 2H), 1.70–1.99 (m, 4H), 1.97–2.07 (m, 1H), 2.05–2.16 (m, 1H), 2.46 (q, *J* = 10.2 Hz, 1H), 3.53 (ddd, *J* = 10.1, 9.0, 7.7 Hz, 1H), 4.12 (td, *J* = 9.0, 2.2 Hz, 1H), 4.62 (s, 1H), 6.72–6.81 (m, 3H), 7.18 (td, *J* = 8.5, 7.2 Hz, 2H). ^13^C NMR (100 MHz, CDCl_3_) δ 145.8, 129.3, 118.7, 113.8, 79.4, 65.7, 39.8, 31.9, 30.8, 28.6, 26.3, 23.2. HRMS m/z calcd for C_14_H_18_NO [M+H]^+^: 216.1388; found: 216.1387.

2-(2-(1H-Benzo[d][1,2,3]triazol-1-yl)-3,3-dimethylcyclohex-1-en-1-yl) ethanol (**15c**): colorless oil; Yield: 92%; ^1^H NMR (400 MHz, CDCl_3_) δ 0.89 (s, 3H), 1.15 (s, 3H), 1.50 (s, 1H), 1.56–1.74 (m, 2H), 1.75–1.83 (m, 2H), 1.83–1.94 (m, 2H), 2.26–2.46 (m, 2H), 3.50 (m, 2H), 7.36 (ddd, *J* = 8.1, 6.6, 1.4 Hz, 1H), 7.40–7.51 (m, 2H), 8.06 (dt, *J* = 8.3, 1.1 Hz, 1H). ^13^C NMR (100 MHz, CDCl_3_) δ 145.1, 138.2, 137.3, 134.8, 127.7, 123.9, 119.9, 110.9, 60.2, 39.2, 36.5, 35.8, 30.0, 28.5, 27.9, 19.0. HRMS m/z calcd for C_16_H_22_N_3_O [M+H]^+^: 272.1763; found: 272.1761.

(4bR,8aR)-8,8-Dimethyl-5,6,7,8-tetrahydro-9H-8a,4b-(epoxyethano)carbazole (**17c**): white solid; Yield: 56%; M.p. 106–107 ℃; ^1^H NMR (400 MHz, CDCl_3_) δ 1.08 (s, 3H), 1.09 (s, 3H), 1.36–1.45 (m, 3H), 1.51–1.66 (m, 2H), 2.05–2.15 (m, 1H), 2.20–2.37 (m, 2H), 3.59 (ddd, *J* = 10.5, 8.1, 6.5 Hz, 1H), 3.91 (td, *J* = 8.1, 1.8 Hz, 1H), 4.41 (s, 1H), 6.63 (dt, *J* = 7.4, 1.0 Hz, 1H), 6.75 (td, *J* = 7.4, 1.0 Hz, 1H), 7.04 (t, *J* = 7.5 Hz, 2H). ^13^C NMR (100 MHz, CDCl_3_) δ 147.8, 136.9, 127.7, 122.3, 119.0, 109.3, 105.9, 67.4, 54.3, 38.5, 37.4, 36.3, 33.6, 26.5, 25.2, 17.5. HRMS m/z calcd for C_16_H_22_NO [M+H]^+^: 244.1701; found: 244.1702.

2-(6-(1H-Benzo[d][1,2,3]triazol-1-yl)spiro[4.5]dec-6-en-7-yl) ethanol (**15d**): colorless oil; Yield: 91%; ^1^H NMR (400 MHz, CDCl_3_) δ 1.03–1.13 (m, 1H), 1.13–1.19 (m, 1H), 1.20–1.31 (m, 1H), 1.35–1.75 (m, 7H), 1.78–1.88 (m, 4H), 2.03–2.15 (m, 1H), 2.26–2.38 (m, 2H), 3.39–3.52 (m, 2H), 7.33 (ddd, *J* = 8.1, 6.6, 1.3 Hz, 1H), 7.38–7.49 (m, 2H), 7.97–8.04 (m, 1H). ^13^C NMR (100 MHz, CDCl_3_) δ 145.0, 138.9, 135.9, 135.1, 127.8, 123.9, 119.9, 110.7, 60.3, 47.5, 37.6, 36.5, 36.0, 36.0, 29.7, 24.2, 23.9, 19.7. HRMS m/z calcd for C_18_H_24_N_3_O [M+H]^+^: 298.1919; found: 298.1921.

(4b′R,8a′R)-6′,7′-Dihydro-5′H,9′H-spiro[cyclopentane-1,8′-[8a,4b](epoxyethano)carbazole] (**17d**): white solid; Yield: 54%; M.p. 84–85 ℃; ^1^H NMR (400 MHz, CDCl_3_) δ 1.34–1.50 (m, 4H), 1.52–1.75 (m, 7H), 1.80–1.90 (m, 2H), 1.92–2.01 (m, 1H), 2.10–2.25 (m, 2H), 3.54 (ddd, *J* = 11.3, 8.3, 5.8 Hz, 1H), 3.91 (ddd, *J* = 8.7, 7.7, 1.3 Hz, 1H), 4.38 (s, 1H), 6.57 (dt, *J* = 7.6, 0.8 Hz, 1H), 6.72 (td, *J* = 7.4, 1.0 Hz, 1H), 6.99–7.06 (m, 2H). ^13^C NMR (100 MHz, CDCl_3_) δ 148.9, 135.7, 127.8, 122.8, 118.6, 108.3, 106.2, 66.4, 54.7, 49.0, 39.7, 35.8, 34.3, 32.5, 32.2, 26.2, 25.0, 17.1. HRMS m/z calcd for C_18_H_24_NO [M+H]^+^: 270.1858; found: 270.1857.

2-(2-(1H-Benzo[d][1,2,3]triazol-1-yl) cyclohex-1-en-1-yl) acetic acid (**15e**): white solid; Yield: 93%; ^1^H NMR (400 MHz, CDCl_3_) δ 1.79–2.00 (m, 4H), 2.38–2.58 (m, 4H), 2.86 (s, 2H), 7.39 (m, 1H), 7.44–7.56 (m, 2H), 8.07 (d, *J* = 8.3 Hz, 1H), 11.34 (s, 1H). ^13^C NMR (100 MHz, CDCl_3_) δ 174.9, 145.2, 133.0, 132.3, 131.7, 128.1, 124.4, 119.9, 110.3, 37.8, 29.8, 29.4, 22.7, 22.0. HRMS m/z calcd for C_14_H_16_N_3_O_2_ [M+H]^+^: 258.1243; found: 258.1244.

(4bR,8aR)-5,6,7,8-Tetrahydro-9H-8a,4b-(epoxyethano)carbazol-11-one (**17e**): yellow solid; Yield: 38%; ^1^H NMR (400 MHz, CDCl_3_) δ 1.24–1.46 (m, 3H), 1.60–1.83 (m, 3H), 2.06–2.12 (m, 1H), 2.36–2.44 (m, 1H), 2.84–2.99 (m, 2H), 4.82 (s, 1H), 6.72 (dt, *J* = 7.8, 0.8 Hz, 1H), 6.85 (td, *J* = 7.5, 1.0 Hz, 1H), 7.06–7.16 (m, 2H). ^13^C NMR (100 MHz, CDCl_3_) δ 174.1, 145.8, 134.9, 128.8, 123.1, 120.7, 110.4, 50.3, 38.4, 33.4, 33.0, 22.1, 20.2. HRMS m/z calcd for C_14_H_16_NO_2_ [M+H]^+^: 230.1181; found: 230.1183.

1-Methyl-2,3,4,9-tetrahydro-1H-carbazole (**22**): light yellow oil; Yield: 50%; ^1^H NMR (400 MHz, CDCl_3_) δ 1.31 (d, *J* = 7.0 Hz, 3H), 1.49–1.58 (m, 1H), 1.71–1.85 (m, 1H), 1.96–2.09 (m, 2H), 2.66–2.78 (m, 2H), 2.93–3.05 (m, 1H), 7.04–7.16 (m, 2H), 7.27–7.33 (m, 1H), 7.44–7.50 (m, 1H), 7.76 (s, 1H). ^13^C NMR (100 MHz, CDCl_3_) δ 138.7, 135.8, 127.8, 121.2, 119.3, 118.1, 110.5, 109.9, 32.5, 28.8, 22.1, 21.3, 20.4. HRMS m/z calcd for C_13_H_16_N [M+H]^+^: 186.1283; found: 186.1278.

2-(2-(1H-Benzo[d][1,2,3]triazol-1-yl)-3,3-dimethylcyclohex-1-en-1-yl)acetic acid (**15f**): white solid; Yield: 94%; M.p. 190–191 °C; ^1^H NMR (400 MHz, CDCl_3_) δ 0.86 (s, 3H), 1.18 (s, 3H), 1.77–1.83 (m, 2H), 1.85–1.96 (m, 2H), 2.30 (dt, *J* = 17.9, 5.8 Hz, 1H), 2.40 (d, *J* = 16.7 Hz, 1H), 2.48 (dt, *J* = 18.1, 6.4 Hz, 1H), 2.58 (d, *J* = 16.7 Hz, 1H), 7.33 (ddd, *J* = 7.9, 6.5, 1.2 Hz, 1H), 7.38–7.47 (m, 2H), 8.04 (d, *J* = 8.2 Hz, 1H), 11.31 (s, 1H). ^13^C NMR (100 MHz, CDCl_3_) δ 175.4, 144.8, 138.9, 134.8, 134.1, 127.9, 124.2, 119.7, 111.1, 39.0, 37.7, 36.6, 30.4, 28.3, 27.6, 18.8. HRMS m/z calcd for C_16_H_20_N_3_O_2_ [M+H]^+^: 286.1556; found: 286.1556.

(4bR,8aR)-8,8-Dimethyl-5,6,7,8-tetrahydro-9H-8a,4b-(epoxyethano)carbazol-11-one (**17f**): white solid; Yield: 63%; M.p. 117–118 °C; ^1^H NMR (400 MHz, CDCl_3_) δ 1.12 (s, 3H), 1.23 (s, 3H), 1.31–1.41 (m, 1H), 1.45–1.54 (m, 3H), 1.55–1.67 (m, 1H), 2.08–2.17 (m, 1H), 2.84–3.01 (m, 2H), 4.81 (s, 1H), 6.72 (dt, *J* = 7.8, 0.7 Hz, 1H), 6.84 (td, *J* = 7.5, 1.0 Hz, 1H), 7.04 (dd, *J* = 7.5, 1.2 Hz, 1H), 7.11 (td, *J* = 7.7, 1.3 Hz, 1H). ^13^C NMR (100 MHz, CDCl_3_) δ 174.8, 145.2, 136.1, 128.7, 122.7, 120.7, 110.4, 51.4, 40.7, 37.3, 37.2, 33.9, 26.4, 23.8, 17.3. HRMS m/z calcd for C_16_H_20_NO_2_ [M+H]^+^: 258.1494; found: 258.1491.

2-(6-(1H-Benzo[d][1,2,3]triazol-1-yl)spiro[4.5]dec-6-en-7-yl)acetic acid (**15g**): white solid; Yield: 84%; M.p. 204–205 °C; ^1^H NMR (400 MHz, CDCl_3_) δ 1.05–1.21 (m, 2H), 1.22–1.32 (m, 1H), 1.39–1.59 (m, 3H), 1.60–1.70 (m, 1H), 1.72–1.91 (m, 4H), 2.11–2.22 (m, 1H), 2.23–2.32 (m, 1H), 2.35 (d, *J* = 16.7 Hz, 1H), 2.43–2.51 (m, 1H), 2.56 (d, *J* = 16.7 Hz, 1H), 7.35 (ddd, *J* = 8.1, 6.2, 1.7 Hz, 1H), 7.38–7.47 (m, 2H), 8.04 (d, *J* = 8.3 Hz, 1H), 10.40 (s, 1H). ^13^C NMR (100 MHz, CDCl_3_) δ 175.4, 144.8, 137.7, 135.0, 134.7, 127.9, 124.2, 119.8, 110.9, 47.6, 37.9, 37.7, 36.5, 36.0, 30.2, 24.3, 24.1, 19.6. HRMS m/z calcd for C_18_H_22_N_3_O_2_ [M+H]^+^: 312.1712; found: 312.1708.

(4b′R,8a′R)-6′,7′-Dihydro-5′H,9′H-spiro[cyclopentane-1,8′-[8a,4b](epoxyethano)carbazol]-11′-one (**17g**): white solid; Yield: 64%; ^1^H NMR (400 MHz, CDCl_3_) δ 1.37–1.79 (m, 12H), 1.90–2.13 (m, 2H), 2.88 (d, *J* = 2.9 Hz, 2H), 4.83 (s, 1H), 6.71 (d, *J* = 7.8 Hz, 1H), 6.83 (t, *J* = 7.4 Hz, 1H), 7.05 (d, *J* = 7.4 Hz, 1H), 7.11 (t, *J* = 7.7 Hz, 1H). ^13^C NMR (100 MHz, CDCl_3_) δ 175.0, 145.6, 135.8, 128.7, 122.8, 120.5, 110.2, 51.6, 48.9, 40.1, 36.1, 33.5, 32.4, 25.3, 23.1, 17.3. HRMS m/z calcd for C_18_H_22_NO_2_ [M+H]^+^: 284.1651; found: 284.1656.

3-(1H-Benzo[d][1,2,3]triazol-1-yl)-2-(2-(methoxymethoxy)ethyl)-4,4-dimethylcyclohex-2-en-1-one (**15h**): light yellow oil; Yield: 72%; ^1^H NMR (400 MHz, CDCl_3_) δ 0.95 (s, 3H), 1.39 (s, 3H), 1.92 (ddd, *J* = 13.3, 7.6, 5.8 Hz, 1H), 2.09–2.25 (m, 3H), 2.67–2.87 (m, 2H), 3.09 (s, 3H), 3.21–3.37 (m, 2H), 4.28–4.35 (m, 2H), 7.36–7.44 (m, 2H), 7.52 (ddd, *J* = 8.1, 7.0, 1.1 Hz, 1H), 8.11 (dt, *J* = 8.4, 1.0 Hz, 1H). ^13^C NMR (100 MHz, CDCl_3_) δ 198.1, 156.8, 145.2, 136.0, 134.0, 128.4, 124.3, 120.3, 110.4, 95.9, 65.3, 55.1, 38.0, 37.1, 34.7, 27.0, 26.3, 26.1. HRMS m/z calcd for C_18_H_24_N_3_O_3_ [M+H]^+^: 330.1818; found: 330.1805.

10-(2-(Methoxymethoxy)ethyl)-9,9-dimethyl-8,9-dihydropyrido[1,2-a]indol-6(7H)-one (**20h**): colorless oil; Yield: 42%; ^1^H NMR (400 MHz, CDCl_3_) δ 1.53 (s, 6H), 1.95 (t, *J* = 6.6 Hz, 2H), 2.81 (dd, *J* = 7.1, 6.2 Hz, 2H), 3.16 (dd, *J* = 8.2, 7.2 Hz, 2H), 3.36 (s, 3H), 3.78 (dd, *J* = 8.3, 7.2 Hz, 2H), 4.65 (s, 2H), 7.26–7.34 (m, 2H), 7.46–7.52 (m, 1H), 8.47–8.53 (m, 1H). ^13^C NMR (100 MHz, CDCl_3_) δ 169.5, 141.3, 134.5, 131.1, 124.8, 123.9, 118.0, 116.8, 113.6, 96.6, 67.3, 55.4, 37.0, 32.7, 30.9, 28.6, 25.7. HRMS m/z calcd for C_18_H_24_NO_3_ [M+H]^+^: 302.1756; found: 302.1751.

6-(1H-Benzo[d][1,2,3]triazol-1-yl)-7-(2-(methoxymethoxy)ethyl)spiro[4.5]dec-6-en-8-one (**15i**): light yellow oil; Yield: 74%; ^1^H NMR (400 MHz, CDCl_3_) δ 1.20–1.28 (m, 2H), 1.39–1.63 (m, 4H), 1.81–1.99 (m, 2H), 2.10–2.22 (m, 3H), 2.31–2.42 (m, 1H), 2.65–2.81 (m, 2H), 3.08 (s, 3H), 3.22–3.34 (m, 2H), 4.26–4.34 (m, 2H), 7.35–7.44 (m, 2H), 7.53 (ddd, *J* = 8.1, 7.0, 1.0 Hz, 1H), 8.11 (dt, *J* = 8.3, 1.0 Hz, 1H). ^13^C NMR (100 MHz, CDCl_3_) δ 198.3, 155.9, 145.2, 136.5, 134.3, 128.5, 124.4, 120.3, 110.3, 95.8, 65.4, 55.1, 48.7, 36.9, 35.2, 34.6, 33.9, 26.5, 24.5, 24.3. HRMS m/z calcd for C_20_H_26_N_3_O_3_ [M+H]^+^: 356.1974; found: 356.1961.

10′-(2-(Methoxymethoxy)ethyl)-7′,8′-dihydro-6′H-spiro[cyclo-pentane-1,9′-pyrido[1,2-a]indol]-6′-one (**20i**): light yellow oil; Yield: 35%; ^1^H NMR (400 MHz, CDCl_3_) δ 1.79–1.96 (m, 6H), 1.99 (dd, *J* = 7.1, 6.0 Hz, 2H), 2.15–2.29 (m, 2H), 2.76 (dd, *J* = 7.1, 5.9 Hz, 2H), 3.10 (dd, *J* = 8.3, 7.2 Hz, 2H), 3.36 (s, 3H), 3.81 (dd, *J* = 8.3, 7.2 Hz, 2H), 4.65 (s, 2H), 7.23–7.32 (m, 2H), 7.46–7.51 (m, 1H), 8.46–8.52 (m, 1H). ^13^C NMR (100 MHz, CDCl_3_) δ 169.7, 141.9, 134.5, 131.1, 124.6, 123.8, 118.0, 116.8, 112.8, 96.5, 67.0, 55.3, 43.4, 39.3, 34.5, 31.7, 25.7, 25.6. HRMS m/z calcd for C_20_H_26_NO_3_ [M+H]^+^: 328.1913; found: 328.1909.

Methyl 2-(2-(1H-benzo[d][1,2,3]triazol-1-yl)-3,3-dimethyl-6-oxocyclohex-1-en-1-yl) acetate (**15j**): light yellow oil; Yield: 73%; ^1^H NMR (400 MHz, CDCl_3_) δ 0.95 (s, 3H), 1.45 (s, 3H), 2.11–2.27 (m, 2H), 2.48 (d, *J* = 16.9 Hz, 1H), 2.77–2.86 (m, 2H), 3.03 (d, *J* = 16.9 Hz, 1H), 3.50 (s, 3H), 7.37–7.47 (m, 2H), 7.51 (ddd, *J* = 8.0, 6.8, 1.0 Hz, 1H), 8.11 (d, *J* = 8.4 Hz, 1H). ^13^C NMR (100 MHz, CDCl_3_) δ 197.3, 170.4, 157.4, 145.2, 134.0, 132.8, 128.7, 124.6, 120.3, 110.3, 52.1, 38.0, 36.9, 34.2, 31.2, 26.7, 25.9. HRMS m/z calcd for C_17_H_20_N_3_O_3_ [M+H]^+^: 314.1505; found: 314.1498.

Methyl 2-(9,9-dimethyl-6-oxo-6,7,8,9-tetrahydropyrido[1,2-a]indol -10-yl) acetate (**20j**): colorless oil; Yield: 32%; ^1^H NMR (400 MHz, CDCl_3_) δ 1.52 (s, 6H), 1.97 (t, *J* = 6.7 Hz, 2H), 2.83 (dd, *J* = 7.1, 6.2 Hz, 2H), 3.70 (s, 3H), 3.86 (s, 2H), 7.26–7.35 (m, 2H), 7.44–7.49 (m, 1H), 8.47–8.53 (m, 1H). ^13^C NMR (100 MHz, CDCl_3_) δ 171.6, 169.4, 142.1, 134.4, 130.8, 125.0, 124.1, 117.9, 116.8, 109.9, 52.3, 36.9, 32.6, 30.9, 30.7, 28.2. HRMS m/z calcd for C_17_H_20_NO_3_ [M+H]^+^: 286.1443; found: 286.1449.

1,1-Dimethyl-1,2,3,9-tetrahydro-4H-carbazol-4-one (**23**): white solid; Yield: 33%; M.p. 240–241 ℃; ^1^H NMR (400 MHz, CDCl_3_) δ 1.48 (s, 6H), 2.10 (dd, *J* = 7.0, 6.0 Hz, 2H), 2.68 (dd, *J* = 7.0, 6.0 Hz, 2H), 7.21–7.26 (m, 2H), 7.34–7.40 (m, 1H), 8.21–8.29 (m, 1H), 8.65 (s, 1H). ^13^C NMR (100 MHz, CDCl_3_) δ 194.2, 158.1, 135.7, 125.1, 123.5, 122.8, 121.9, 111.6, 111.1, 38.7, 35.6, 32.2, 27.5. HRMS m/z calcd for C_14_H_16_NO [M+H]^+^: 214.1232; found: 214.1227.

Methyl 2-(6-(1H-benzo[d][1,2,3]triazol-1-yl)-8-oxospiro[4.5]dec-6-en-7-yl)acetate (**15k**): light yellow oil; Yield: 66%; ^1^H NMR (400 MHz, CDCl_3_) δ 1.19–1.29 (m, 2H), 1.37–1.58 (m, 2H), 1.60–1.70 (m, 2H), 1.96 (ddd, *J* = 12.9, 7.7, 4.3 Hz, 1H), 2.20 (t, *J* = 6.3 Hz, 2H), 2.38–2.51 (m, 2H), 2.77 (td, *J* = 6.9, 5.9, 4.1 Hz, 2H), 3.00 (d, *J* = 16.8 Hz, 1H), 3.49 (s, 3H), 7.38–7.48 (m, 2H), 7.49–7.56 (m, 1H), 8.11 (d, *J* = 8.3 Hz, 1H). ^13^C NMR (100 MHz, CDCl_3_) δ 197.6, 170.4, 156.6, 145.1, 134.2, 133.3, 128.7, 124.6, 120.3, 110.3, 52.1, 48.7, 36.8, 34.8, 34.8, 33.9, 31.2, 24.6, 24.5. HRMS m/z calcd for C_19_H_22_N_3_O_3_ [M+H]^+^: 340.1661; found: 340.1653.

Methyl 2-(6′-oxo-7′,8′-dihydro-6′H-spiro[cyclopentane-1,9′-pyrido[1,2-a]indol]-10′- yl)acetate (**20k**): light yellow oil; Yield: 18%; ^1^H NMR (400 MHz, CDCl_3_) δ 1.81–1.96 (m, 6H), 2.02 (t, *J* = 6.5 Hz, 2H), 2.15–2.24 (m, 2H), 2.78 (dd, *J* = 7.0, 6.0 Hz, 2H), 3.71 (s, 3H), 3.80 (s, 2H), 7.27–7.35 (m, 2H), 7.40–7.47 (m, 1H), 8.43–8.53 (m, 1H). ^13^C NMR (100 MHz, CDCl_3_) δ 171.6, 169.7, 142.9, 134.4, 130.8, 124.9, 124.0, 118.0, 116.8, 109.4, 52.4, 43.2, 39.3, 34.7, 31.8, 30.7, 25.9. HRMS m/z calcd for C_19_H_22_NO_3_ [M+H]^+^: 312.16; found: 312.1588.

2,3-Dihydrospiro[carbazole-1,1′-cyclopentan]-4(9H)-one (**24**): white solid; Yield: 45%; M.p. 229–230 °C; ^1^H NMR (400 MHz, CDCl_3_) δ 1.84–1.93 (m, 4H), 1.93–2.08 (m, 4H), 2.14 (dd, *J* = 7.0, 5.9 Hz, 2H), 2.65 (dd, *J* = 7.0, 5.9 Hz, 2H), 7.20–7.26 (m, 2H), 7.32–7.41 (m, 1H), 8.21–8.29 (m, 1H), 8.89 (s, 1H). ^13^C NMR (100 MHz, CDCl_3_) δ 194.5, 158.3, 135.8, 125.1, 123.4, 122.7, 121.7, 112.3, 111.1, 43.3, 38.2, 36.6, 36.2, 25.5. HRMS m/z calcd for C_16_H_18_NO [M+H]^+^: 240.1388; found: 240.1386.

## 4. Conclusions

In summary, we systematically explored the photo-induced denitrogenative transformations of various 1-alkenylbenzotrizoles. Through rationally manipulating the structures of 1-alkenylbenzotrizole precursors, we could access two different types of polycyclic skeletons associated with monoterpene indole alkaloids. Specifically, starting from 1-alkenylbenzotrizoles bearing a suitable nucleophile (e.g., OH and COOH) on their side chains, the 4a,9a-heterocycle-fused tetrahydrocarbazole skeleton could be assembled through a photo-induced denitrogenative annulation followed by the cyclization of the nucleophilic side chain onto the indolenine intermediate. Comparably, for 1-alkenylbenzotrizoles with a masked side chain, the resulting indolenine intermediate will divert to the other tricyclic skeleton, namely dihydropyrido[1,2-a]indolone, through further skeletal rearrangement reaction. Taken together, the results of the present study clearly showcase the appealing pottnial of photo-induced denitrogenative transformations of 1-alkenylbenzotrizoles, which, as we anticipated, may find considerable application in the total synthesis of complex natural products.

## Figures and Tables

**Table 1:** Condition optimization of the photoreaction.

Entry	Reactant	Solvent	Light Source (nm)	Yield (%)
1	**15a**	MeCN	254	**17a:** 0%
2	**15b**	MeCN	254	**17b:** 23%
3	**15b**	THF	254	**17b:** 30%
4	**15b**	Ethyl acetate	254	**17b:** 22%
5	**15b**	1,4-Dioxane	254	**17b:** 22%
6	**15b**	MeOH	254	**17b:** 14%
7	**15b**	DMF	254	**17b:** 16%
8	**15b**	Toluene	254	**17b:** 15%
9	**15b**	THF	311	**17b:** 27%
10	**15b**	MeCN	311	**17b:** 20%
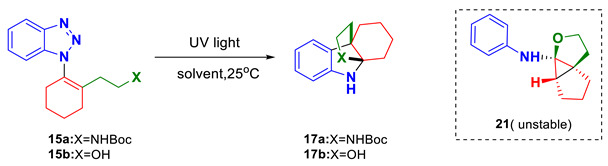				

Without special note, all reactions were conducted with a 40 mg scale of substrate in 5 mL solvent under nitrogen protection.

## Data Availability

Not applicable.

## Supplementary Materials

The following supporting information can be downloaded at: https://www.mdpi.com/article/10.3390/molecules28010363/s1, Scheme S1: The general procedure for the preparation of 1-alkenylbenzotriazoles; Figure S1: Setup of the photoreactor. Furthermore, all ^1^H and C NMR spectra of synthesized compounds are shown [42,43,44,45,46,47,48,49,50].

## Sample Availability

Samples of the compounds are available from the authors.

## References

[1] Mohammed A.E., Abdul-Hameed Z.H., Alotaibi M.O., Bawakid N.O., Sobahi T.R., Abdel-Lateff A., Alarif W.M. (2021). Chemical diversity and bioactivities of monoterpene indole alkaloids (mias) from six apocynaceae genera. Molecules.

[2] Rosales P.F., Bordin G.S., Gower A.E., Moura S. (2020). Indole alkaloids: 2012 until now, highlighting the new chemical structures and biological activities. Fitoterapia.

[3] Adams G.L., Smith A.B., Knölker H.-J. (2016). Chapter three—The chemistry of the akuammiline alkaloids. The Alkaloids: Chemistry and Biology.

[4] Eckermann R., Gaich T. (2013). The akuammiline alkaloids; origin and synthesis. Synthesis.

[5] Hosoya K., Iida K., Odagi M., Nagasawa K. (2021). Recent advances in synthetic strategies for the c4a,c9a-fused tetracyclic hydrocarbazole core structure of minfiensine and related akuammiline alkaloids. Heterocycles.

[6] Koyama K., Hirasawa Y., Nugroho A.E., Kaneda T., Hoe T.C., Chan K.-L., Morita H. (2012). Alsmaphorazines c–e, indole alkaloids from alstonia pneumatophora. Tetrahedron.

[7] Pfaffenbach M., Gaich T. (2016). The diaza[5.5.6.6]fenestrane skeleton—Synthesis of leuconoxine alkaloids. Chem. Eur. J..

[8] Gao B., Yao F., Zhang Z., Ding H. (2021). Total synthesis of (+)-alsmaphorazine c and formal synthesis of (+)-strictamine: A photo-fries approach. Angew. Chem. Int. Ed..

[9] Katritzky A.R., Rachwal S. (2010). Synthesis of heterocycles mediated by benzotriazole. 1. Monocyclic systems. Chem. Rev..

[10] Katritzky A.R., Rachwal S. (2011). Synthesis of heterocycles mediated by benzotriazole. 2. Bicyclic systems. Chem. Rev..

[11] Bollikolla H.B., Boddapati S.N.M., Thangamani S., Mutchu B.R., Alam M.M., Hussien M., Jonnalagadda S.B. (2022). Advances in synthesis and biological activities of benzotriazole analogues: A micro review. J. Heterocycl. Chem..

[12] Briguglio I., Piras S., Corona P., Gavini E., Nieddu M., Boatto G., Carta A. (2015). Benzotriazole: An overview on its versatile biological behavior. Eur. J. Med. Chem..

[13] Akter M., Rupa K., Anbarasan P. (2022). 1,2,3-triazole and its analogues: New surrogates for diazo compounds. Chem. Rev..

[14] Wang Y., Wang Z., Tang Y. (2020). Renaissance of ring-opening chemistry of benzotriazoles: New wine in an old bottle. Chem. Rec..

[15] Yu J., Singh A.S., Yan G., Yu J., Tiwari V.K. (2020). Recent developments on denitrogenative functionalization of benzotriazoles. Synthesis.

[16] Li W., Zhang J. (2020). Synthesis of heterocycles through denitrogenative cyclization of triazoles and benzotriazoles. Chem. Eur. J..

[17] An T., Liu C., Yin Y., Wu X.-F., Yin Z. (2022). Palladium-catalyzed denitrogenative carbonylation of benzotriazoles with cr(co)6 as the carbonyl source. Organometallics.

[18] Li Y.-L., Zhang P.-C., Wu H.-H., Zhang J. (2021). Palladium-catalyzed asymmetric tandem denitrogenative heck/tsuji–trost of benzotriazoles with 1,3-dienes. J. Am. Chem. Soc..

[19] Hari Balakrishnan M., Mannathan S. (2020). Palladium/copper-catalyzed denitrogenative alkylidenation and ortho-alkynylation reaction of 1,2,3-benzotriazin-4(3h)-ones. Org. Lett..

[20] Alekseyev R.S., Kurkin A.V., Yurovskaya M.A. (2009). Γ-carbolines and their hydrogenated derivatives. 1. Aromatic γ-carbolines: Methods of synthesis, chemical and biological properties (review). Chem. Heterocycl. Compd..

[21] Alimi I., Remy R., Bochet C.G. (2017). Photochemical c–h activation: Generation of indole and carbazole libraries, and first total synthesis of clausenawalline d. Eur. J. Org. Chem..

[22] Wender P.A., Cooper C.B. (1986). The photochemistry of 1-alkenylbenzotriazoles: Methodology for the synthesis of indoles. Tetrahedron.

[23] Dutton J.K., Pleynet D.P.M., Johnson A.P. (1999). Synthesis of hindered spiro-oxindoles by photolysis of 1-(1-alkenyl)benzotriazoles. Tetrahedron.

[24] Kazuo T., Mamoru O., Teijiro Y. (1972). The photochemical decomposition of benzotriazoles. Bull. Chem. Soc. Jpn..

[25] Al-Jalal N.A., Ibrahim M.R., Al-Awadi N.A., Elnagdi M.H., Ibrahim Y.A. (2014). Photochemistry of benzotriazoles: Generation of 1,3-diradicals and intermolecular cycloaddition as a new route toward indoles and dihydropyrrolo[3,4-b]indoles. Molecules.

[26] Teders M., Gómez-Suárez A., Pitzer L., Hopkinson M.N., Glorius F. (2017). Diverse visible-light-promoted functionalizations of benzotriazoles inspired by mechanism-based luminescence screening. Angew. Chem. Int. Ed..

[27] Teders M., Pitzer L., Buss S., Glorius F. (2017). Regioselective synthesis of 2-substituted indoles from benzotriazoles and alkynes by photoinitiated denitrogenation. ACS Catal..

[28] Wang Y., Wu Y., Li Y., Tang Y. (2017). Denitrogenative suzuki and carbonylative suzuki coupling reactions of benzotriazoles with boronic acids. Chem. Sci..

[29] Wang Y., Li Y., Fan Y., Wang Z., Tang Y. (2017). Palladium-catalyzed denitrogenative functionalizations of benzotriazoles with alkenes and 1,3-dienes. Chem. Commun..

[30] Wang Y., Wang Z., Chen X., Tang Y. (2018). Palladium-catalyzed denitrogenative cycloadditions and alkenylations of benzotriazoles with alkynes. Org. Chem. Front..

[31] Teuber H.-J., Cornelius D., Worbs E. (1964). Indole aus cyclischen β-dicarbonyl-verbindungen, i. Tetrahedron Lett..

[32] Ban Y., Yoshida K., Goto J., Oishi T., Takeda E. (1983). Ishigamori A synthetic road to the forest of strychnos, aspidosperma, schizozygane and eburnamine alkaloids by way of the novel photoisomerization. Tetrahedron.

[33] Ban Y., Yoshida K., Goto J., Oishi T. (1981). Novel photoisomerization of 1-acylindoles to 3-acylindolenines. General entry to the total synthesis of strychnos and aspidosperma alkaloids. J. Am. Chem. Soc..

[34] Somei M., Natsume M. (1973). Photochemical rearrangements for the syntheses of 3-, 4-, and 6-substituted indoles. Tetrahedron Lett..

[35] Takechi H., Machida M., Kanaoka Y. (1988). Intramolecular photoreactions of phthalimide-alkene systems. Oxetane formation of n-(&omega;-indol-3-ylalkyl)phthalimides. Chem. Pharm. Bull..

[36] Teuber H.-J., Worbs E., Cornelius D. (1982). Indole aus cyclischen 1.3-dicarbonylverbindungen mit substituierter 2-stellung. Arch. Pharm..

[37] Edmondson S.D., Mastracchio A., Parmee E.R. (2000). Palladium-catalyzed coupling of vinylogous amides with aryl halides:  Applications to the synthesis of heterocycles. Org. Lett..

[38] Li W., Dong Z., Zhang Y., Zeng Z., Usman M., Liu W.-B. (2019). Cu-catalyzed arylation/acyl migration cascade reaction of enaminones: Access to n-fused polycyclic and 2,3-disubstituted indoles. J. Org. Chem..

[39] Dong Z., Zhang X.-W., Li W., Li Z.-M., Wang W.-Y., Zhang Y., Liu W., Liu W.-B. (2019). Synthesis of n-fused polycyclic indoles via ligand-free palladium-catalyzed annulation/acyl migration reaction. Org. Lett..

[40] Barker S.J., Storr R.C. (1990). Flash-pyrolysis of 1-vinylbenzotriazoles. J. Chem. Soc. Perkin Trans..

[41] Naruse Y., Ito Y., Inagaki S. (1991). Highly regioselective functionalization of 2,3-dialkyl substituents on indoles. J. Org. Chem..

[42] Bhunia S., Ghorpade S., Huple D.B., Liu R.S. (2012). Gold-catalyzed oxidative cyclizations of cis-3-en-1-ynes to form cyclopentenone derivatives. Angew. Chem. Int. Ed..

[43] Das J., Sarkar A., Ghosh P. (2018). Friedelane triterpenoids: Transformations toward A-ring modifications including 2-homoderivatives. N. J. Chem..

[44] Lee E., Shin I.J., Kim T.S. (1990). Enantiocontrolled synthesis of quaternary carbon centers via anionic oxy-cope rearrangement: An efficient synthesis of (+)-Dihydromayurone. J. Am. Chem. Soc..

[45] Wong L.S.-M., Sherburn M.S. (2003). IMDA-radical cyclization approach to (+)-Himbacine. Org. Lett..

[46] Jiang X.G., Zhang J.S., Ma S.M. (2016). Iron catalysis for room temperature aerobic oxidation of alcohols to carboxylic acids. J. Am. Chem. Soc..

[47] Guo Y.L., Quan T.F., Lu Y.D., Luo T.P. (2017). Enantioselective total synthesis of (+)-Wortmannin. J. Am. Chem. Soc..

[48] Wang C., Yamamoto H. (2014). Tungsten-catalyzed asymmetric epoxidation of allylic and homoallylic alcohols with hydrogen peroxide. J. Am. Chem. Soc..

[49] Li Z., Watkins E.B., Liu H., Chittiboyina A.G., Carvalho P.B., Avery M.A. (2008). 1,3-Diaxially substituted trans-decalins: Potential nonsteroidal human progesterone receptor inhibitors. J. Org. Chem..

[50] Daniel D., Andrew A.S. (1999). Reductive N-alkylation of amides, carbamates and ureas. Tetrahedron Lett..

