# Supplementary material for: Natural Product-Oriented Photo-Induced Denitrogenative Annulations of 1-Alkenylbenzotriazoles
Source: Molecules. 2023 Jan 2;28(1):363. doi: 10.3390/molecules28010363 (PMC9823906; doi:10.3390/molecules28010363)
Supplement: Supplementary file 1 [file molecules-28-00363-s001.zip › molecules-2080130-supplementary.pdf]

Supplementary Materials

Natural Product-Oriented Photo-Induced Denitrogenative Annulation of 1-Alkenylbenzotriazoles

Zhiguo Wang [1,2], Yi Chen [1], Zhen Dong [1], and Yefeng Tang [1,]*

1 School of Pharmaceutical Sciences, MOE Key Laboratory of Bioorganic Phosphorus Chemistry & Chemical Biology, Tsinghua University, Beijing 100084, China.

2 College of Chemical Engineering, Qinghai University, Xining 810016, China.

** Correspondence: yefengtang@tsinghua.edu.cn*

CONTENTS

1. The general procedures for the preparation of 1-alkenylbenzotriazoles 2-4

2. Setup of the photoreactor 5

3. ^{1}H and ^{13}C NMR spectra of synthesized compounds 6-33

1. The general procedures for the preparation of 1-alkenylbenzotriazoles

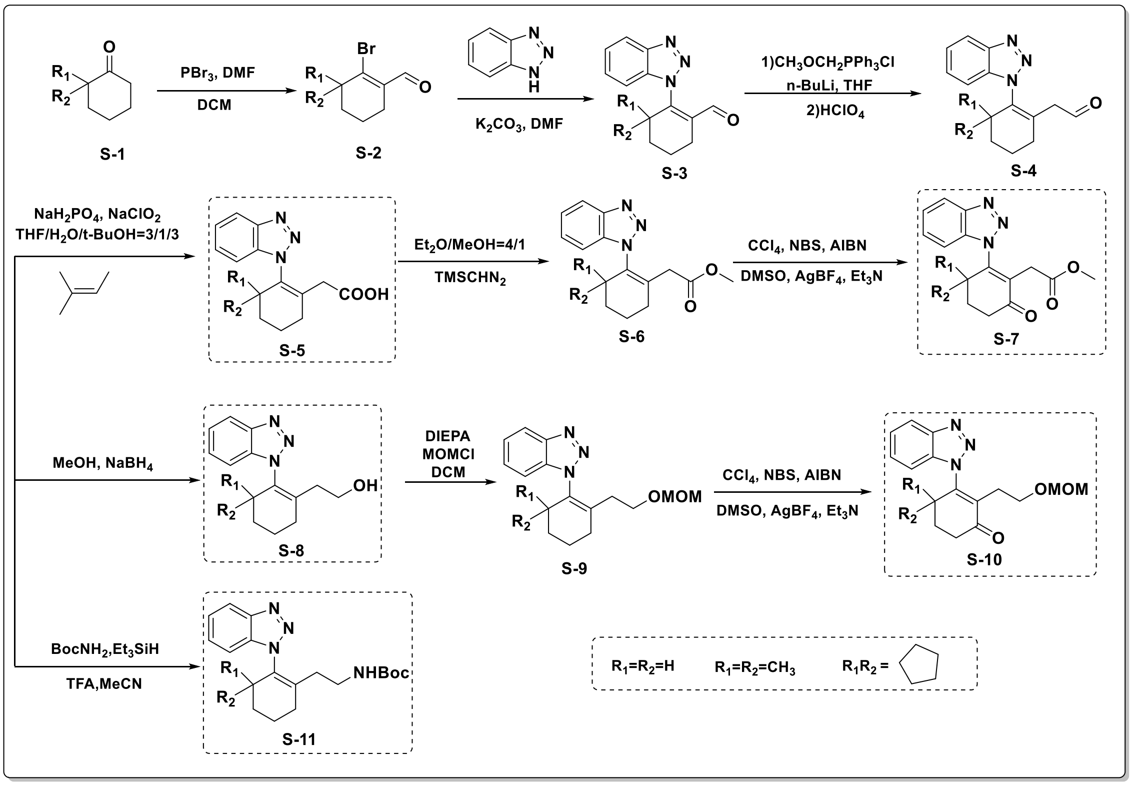

Scheme S1. The general procedure for the preparation of 1-alkenylbenzotriazoles

Synthesis of compound S-2[1]

To a solution of dichloromethane (30 mL) and DMF (5.3 mL, 68.85 mmol) was added PBr_3 (7.2mL, 76.5 mmol) at 0 ℃ slowly; the resulting solution was stirred for 45 min before the addition of cyclohexanone **S-1** (25.5 mmol). The reaction mixture was stirred for 40 h at room temperature. To the resulting solution was added cold water, saturated $NaHCO_3$, and then extracted with dichloromethane. The extracts were washed with saturated NaCl solution, dried over anhydrous $MgSO_4$, and concentrated under reduced pressure. The residue was purified by elution through a short silica column to afford compound **S-2**.

Synthesis of compound S-3[2]

Compound **S-2** (0.25 mmol), 1H-benzotriazole (74.4 mg, 0.625 mmol), and K_2CO_3 (172.5mg, 1.25 mmol) in dry DMF (5 mL) was heated at 80℃-120℃ under N_2. The reaction was complete as monitored by TLC. After cooling to room temperature, the reaction mixture was poured onto ice-cold water and was extracted with dichloromethane and the combined organic solvent was washed successively with water and brine solution, dried ($MgSO_4$) and the solvent was removed at reduced pressure. Finally, column chromatography furnished compound **S-3**.

Synthesis of compound S-4[3]

Triphenyl (methoxymethyl) phosphonium chloride (362 mg, 1.056 mmol) was placed in

a three-neck flask under nitrogen and suspended in 2 mL of THF. n-Butyllithium (0.5 mL of a 2.2 M hexane solution) was slowly added with a syringe at 0 ℃. After 10 min, **S-3** (0.88 mmol) in THF (0.3ml) was added to the mixture. The mixture was further stirred for 2 h at room temperature, quenched with water, and worked up in ether. The crude product was purified by column chromatography to give the enol ether.

Perchloric acid (0.86 mL) was added to the enol ether (0.2 mmol) in THF (1.5 mL) at room temperature. After 30 min, the reaction mixture was poured into water and worked up with ether, the crude product was purified by column chromatography to give compound **S-4**.

Synthesis of compound S-5[4]

Compound **S-4** (0.083 mmol), NaH_2PO_4 (29.9 mg, 0.249 mmol), $NaClO_2$ (22.5 mg, 0.249 mmol), THF (0.3 mL), water (0.1 mL), t-BuOH (0.3 mL), and 2-methyl-2-butene (46.6mg, 0.664 mmol) was stirred vigorously for 2h at rt. The mixture was then diluted with saturated aqueous NH_4Cl and extracted with ethyl acetate. The combined organic extracts were washed with brine, dried over $MgSO_4$, filtered, concentrated and through a short silica column to give compound **S-5**.

Synthesis of compound S-6[5]

To a round flask were added compound **S-5** (0.1 mmol) and 0.5 mL of Et_2O/MeOH (4/1 by volume) at room temperature. The resulting mixture was then cooled to 0℃. trimethylsilyldiazomethane（$TMSCHN_2$ 2.0 M in hexanes, 0.075 mL, 0.15 mmol) was added dropwise. The flask was warmed to room temperature naturally. The reaction was complete as monitored by TLC. After evaporation, the residue was purified by chromatography on silica gel to afford compound **S-6**.

Synthesis of compound S-7[6]

To a solution of compound **S-6** (0.293 mmol) in CCl_4 (10 mL) at room temperature under N_2 was added N-bromosuccinimide (78.32mg, 0.44 mmol) and azobisisobutyronitrile (14.43 mg, 0.0879 mmol), and was degassed for 15 min under N_2 balloon. The mixture was heated to reflux for 1 h, then cooled to room temperature, and concentrated in vacuo. To a solution of the residue in dry DMSO (6.7 mL) was added a solution of $AgBF_4$ (68.4 mg, 0.3516 mmol) in dry DMSO (6.7 mL) dropwise at room tempeture, after which AgBr precipitated immediately. The resulting suspension was stirred for 30 min, after which time Et_3N (0.082 mL, 0.586 mmol) was added and the reaction mixture was stirred for an additional 2 h. Then the mixture was filtered through a short column of silica gel and the column was washed with EtOAc. The filtrate was poured into water and extracted with EtOAc three times. The organic layer was dried over $MgSO_4$ and concentrated in vacuo. The residue was purified by column chromatography on silica gel to afford compound **S-7**.

Synthesis of compound S-8[7]

To a solution of compound **S-4** (1 mmol) in MeOH (1 mL) was added $NaBH_4$ (113.49mg 3 mmol) portion wise at 0℃. The resulting mixture was stirred for 30 min at 0℃ and further stirred at room temperature. After quenching with water, the contents

were extracted with ether, The combined organic layers were washed with brine, dried over $MgSO_4$, filtered and evaporated under reduced pressure. The crude product was purified through column chromatography to afford compound **S-8**.

Synthesis of compound S-9[8]

To a solution of **S-8** (2.36 mmol) in DCM (9 mL) was added diisopropylethylamine (2 ml, 11.8 mmol) and chloromethyl methyl ether (0.75 ml, 9.44 mmol) at 0℃. The reaction mixture was warmed to room temperature and stirred. The resulting solution was concentrated under reduced pressure and the resulting residue was purified by silica gel chromatography to provide the required compound **S-9**.

Synthesis of compound S-10

The synthesis procedure for preparation of compound **S-10** is the same as **S-7**.

Synthesis of compound S-11[9]

A solution of **S-4** (0.48 mmol), t-butylcarbamate (168.7 mg, 1.44 mmol), Et_3SiH (0.23 mL, 1.45 mmol), TFA (0.074 mL,0.96 mmol) in CH_3CN (5.8 mL) was stirred room temperature. The resulting solution were diluted with Et_2O, washed with $NaHCO_3$ sol. and brine. The organic layer was dried and the solvents evaporated. The resulting residue was purified by silica gel chromatography to provide the required compound **S-11**.

References:

1. Bhunia, S.; Ghorpade, S.; Huple, D. B.; Liu, R. S. Gold-catalyzed oxidative cyclizations of cis-3-en-1-ynes to form cyclopentenone derivatives. *Angew. Chem. Int. Ed.* 2012, 51, 2939-2942.
2. Das, J.; Sarkar, A.; Ghosh, P. Friedelane triterpenoids: transformations toward A-ring modifications including 2-homoderivatives. *New J. Chem.* 2018, 42, 6673-6688.
3. Lee, E.; Shin, I. J.; Kim, T. S. Enantiocontrolled synthesis of quaternary carbon centers via anionic oxy-cope rearrangement: an efficient synthesis of (+)-Dihydromayurone. *J. Am. Chem. Soc.* 1990, 112, 260-264.
4. Wong, L. S.-M.; and Sherburn, M. S. IMDA-radical cyclization approach to (+)-Himbacine. *Org. Lett.* 2003, 5, 3603-3606.
5. Jiang, X. G.; Zhang, J. S.; Ma, S. M. Iron catalysis for room temperature aerobic oxidation of alcohols to carboxylic acids. *J. Am. Chem. Soc.* 2016, 138, 8344-8347.
6. Guo, Y. L.; Quan, T. F.; Lu, Y. D.; Luo, T. P. Enantioselective total synthesis of (+)-Wortmannin. *J. Am. Chem. Soc.* 2017, 139, 6815-6818.
7. Wang, C.; Yamamoto, H. Tungsten-catalyzed asymmetric epoxidation of allylic and homoallylic alcohols with hydrogen peroxide. *J. Am. Chem. Soc.* 2014, 136, 1222-1225.
8. Li, Z.; Watkins, E. B.; Liu, H.; Chittiboyina, A. G.; Carvalho, P. B.; Avery, M. A. 1,3-Diaxially substituted trans-decalins: potential nonsteroidal human progesterone receptor inhibitors. *J. Org. Chem.* 2008, 73, 7764-7767.
9. Daniel, D.; Andrew, A. S. Reductive N-alkylation of amides, carbamates and ureas *Tetrahedron Lett*. 1999, 40,2295-2298.

2. Setup of the photoreactor

The set-up below (UVC lamp, 254 nm, 25 W×6) has been used for the photo-induced denitrogenative annulations of 1-alkenylbenzotriazoles.

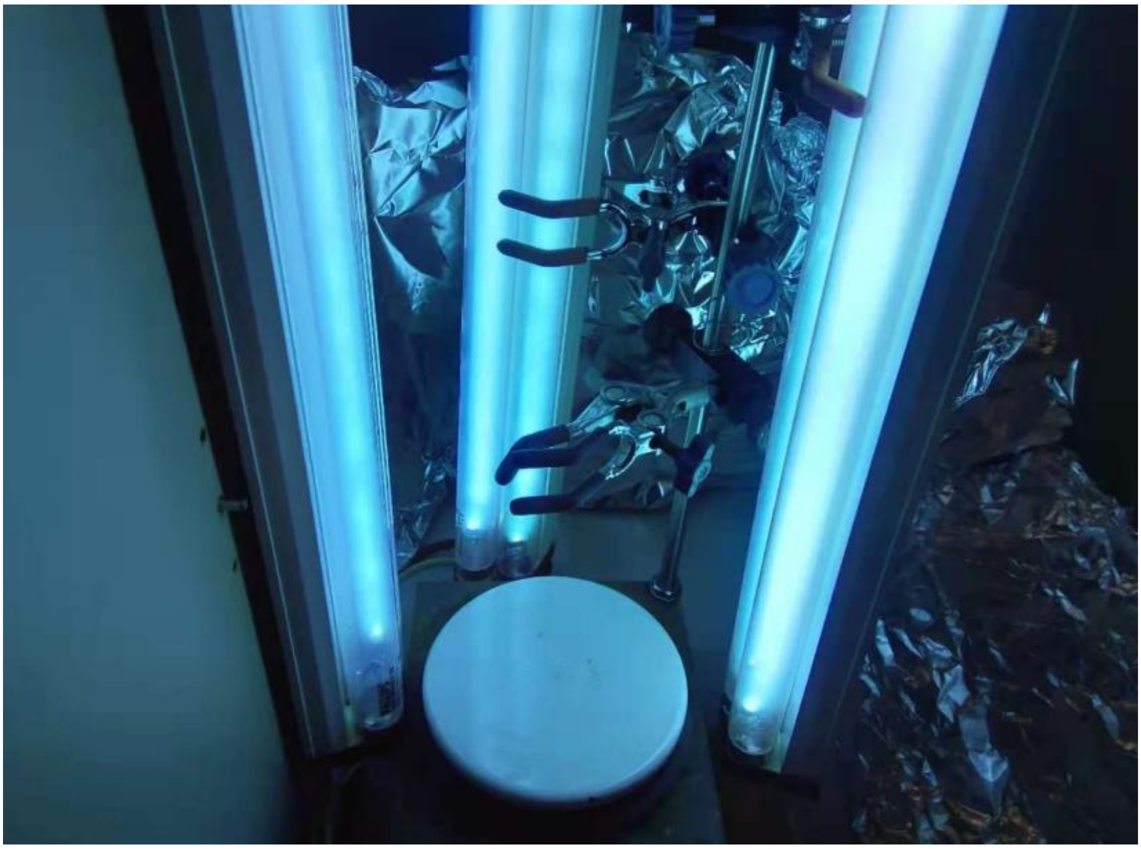

Figure S1. Setup of the photoreactor

3. ^{1}H NMR and ^{13}C NMR spectra of compounds

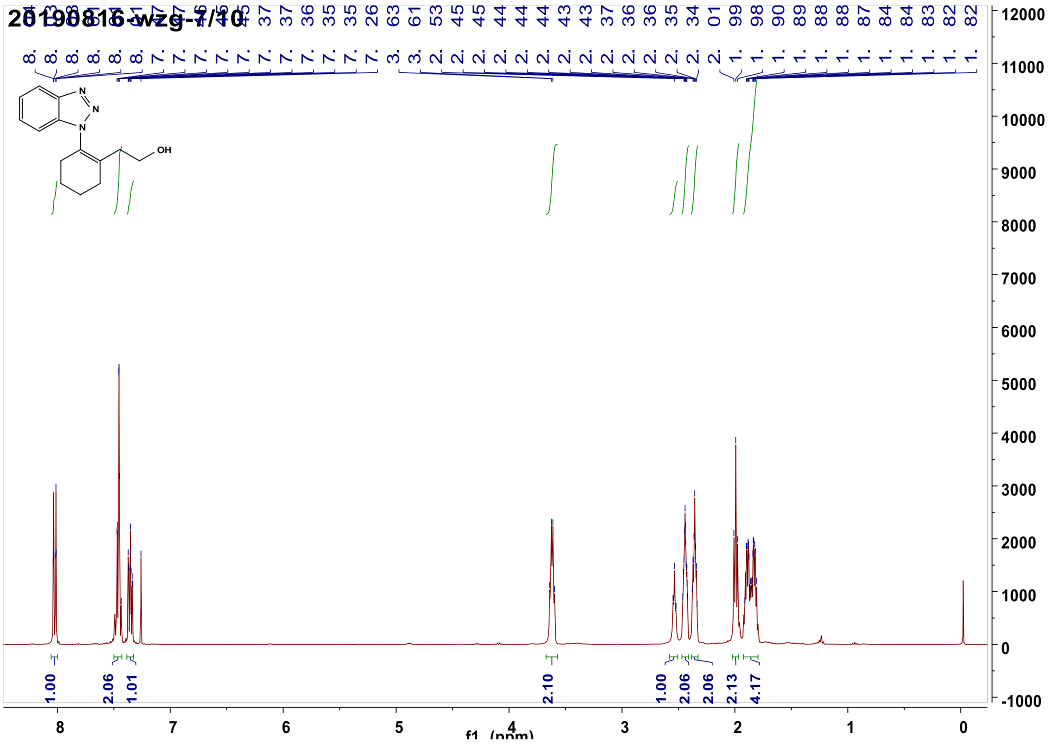

^{1}H NMR spectrum of compound **15b**

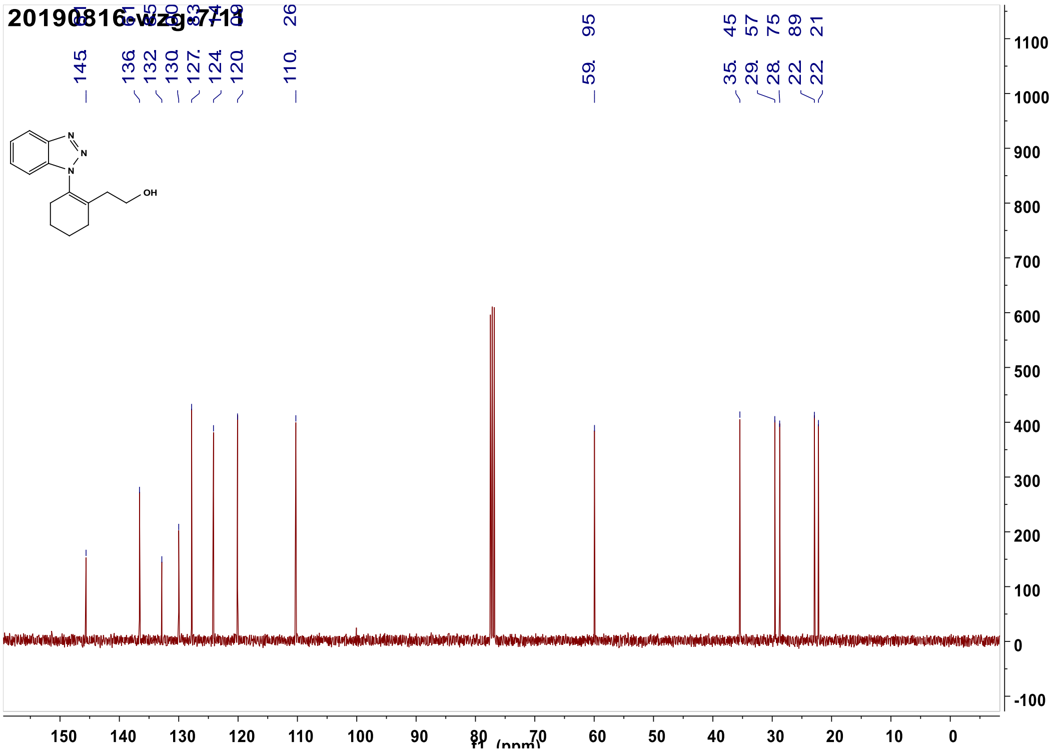

^{13}C NMR spectrum of compound **15b**

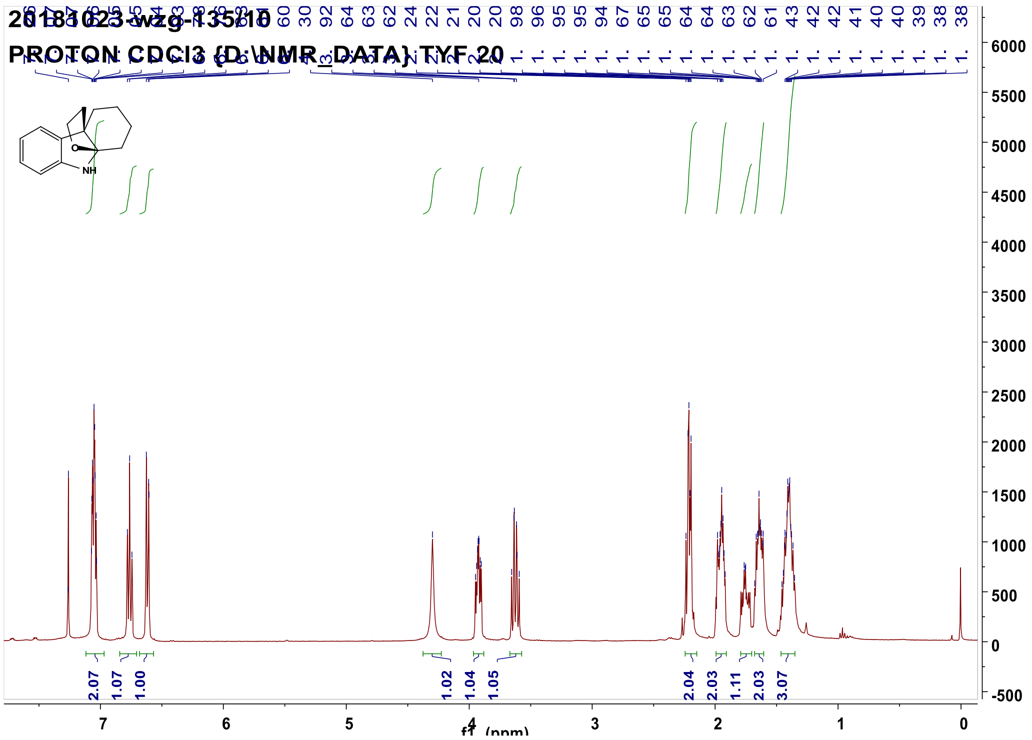

^{1}H NMR spectrum of compound **17b**

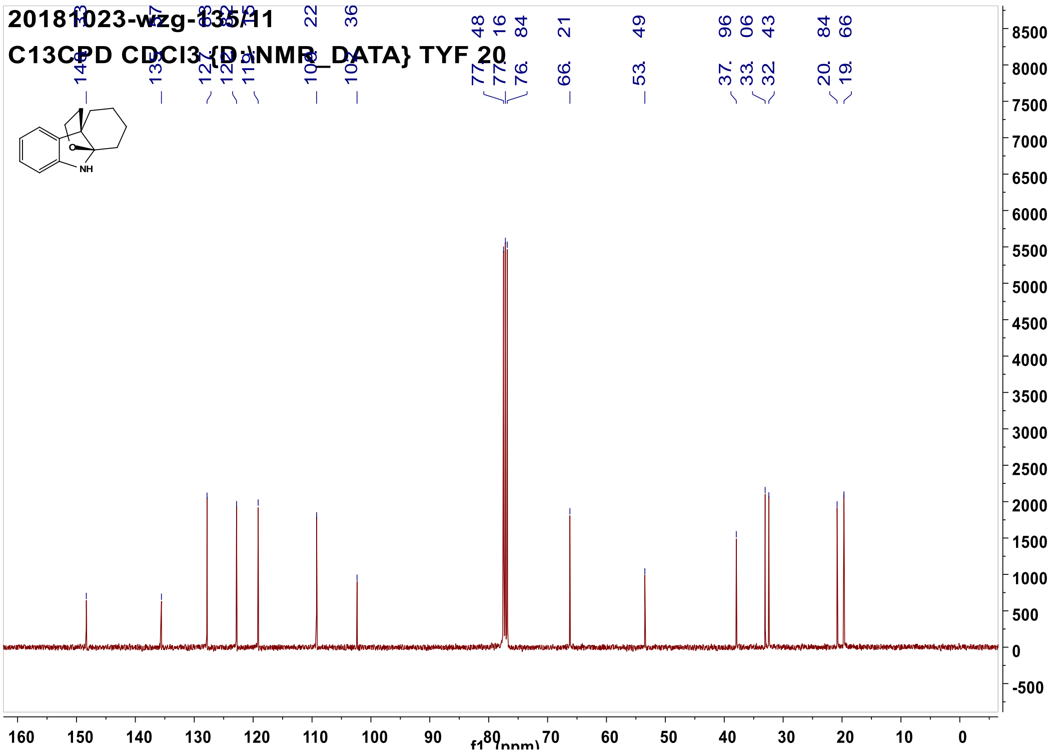

^{13}C NMR spectrum of compound **17b**

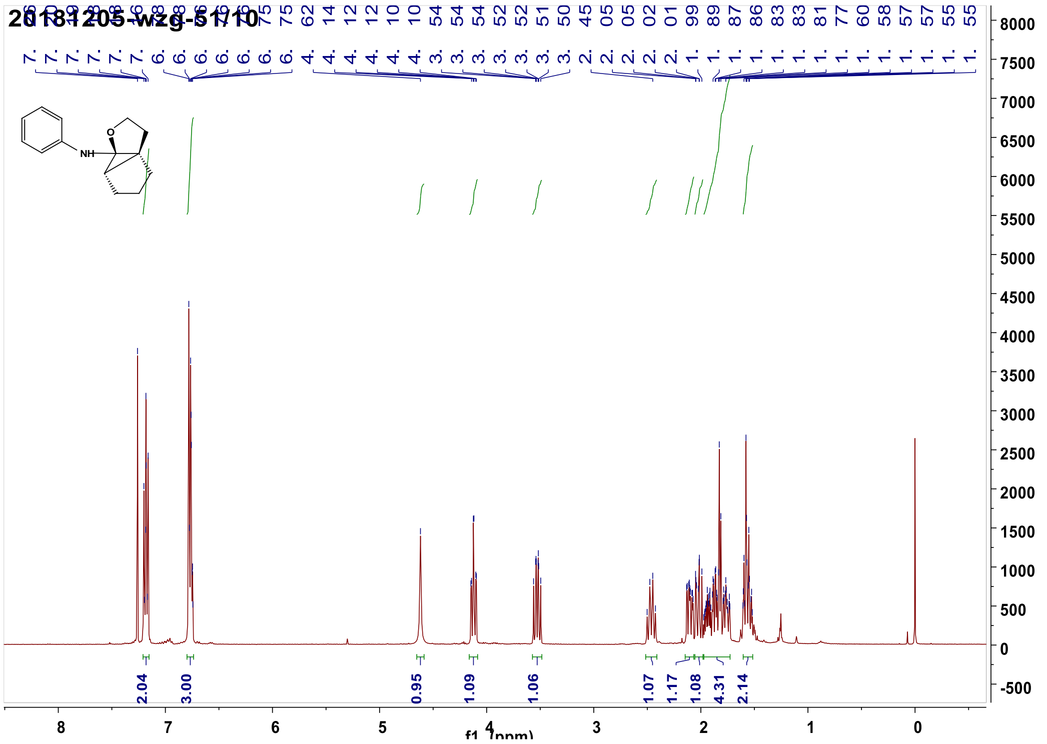

^{1}H NMR spectrum of compound **21**

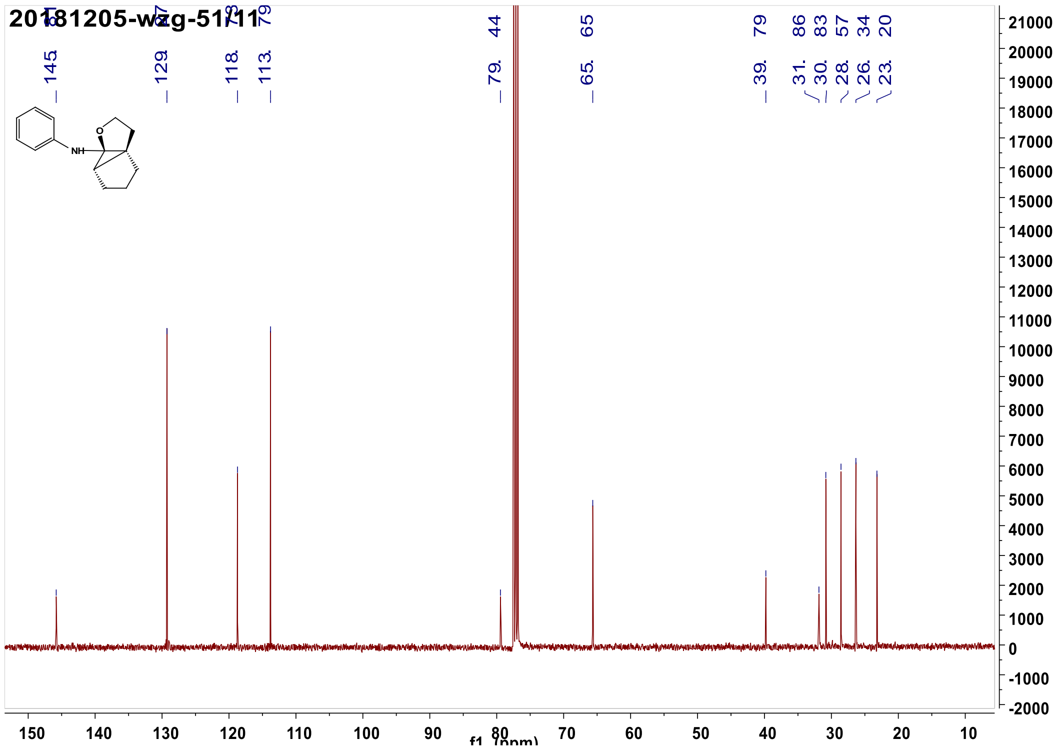

^{13}C NMR spectrum of compound **21**

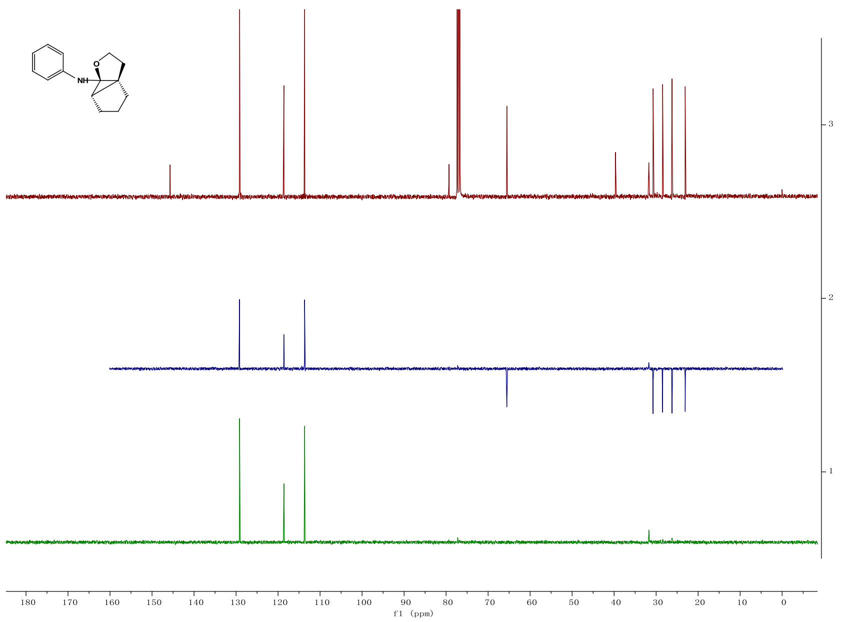

The DEPT90° and 135° spectrum of compound **21**

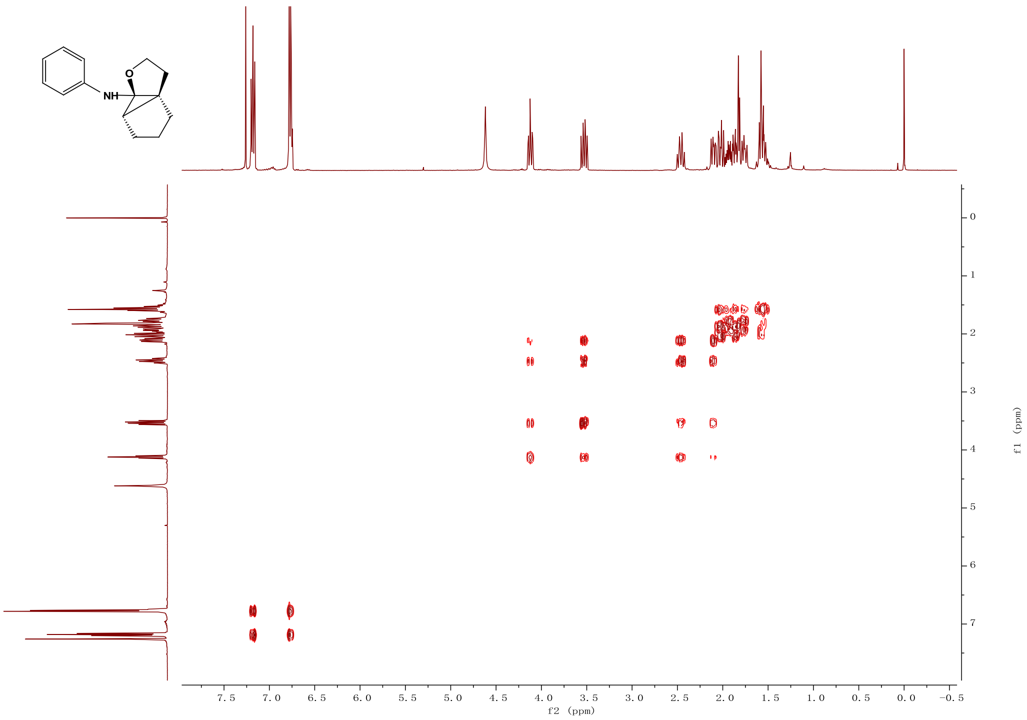

The COSY spectrum of compound **21**

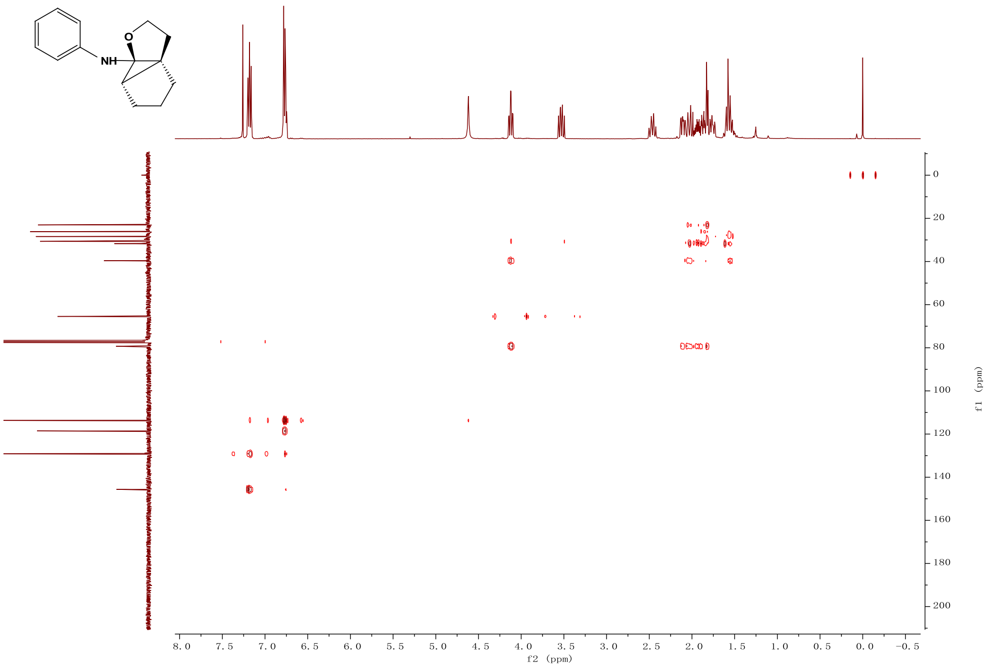

The HMBC spectrum of compound **21**

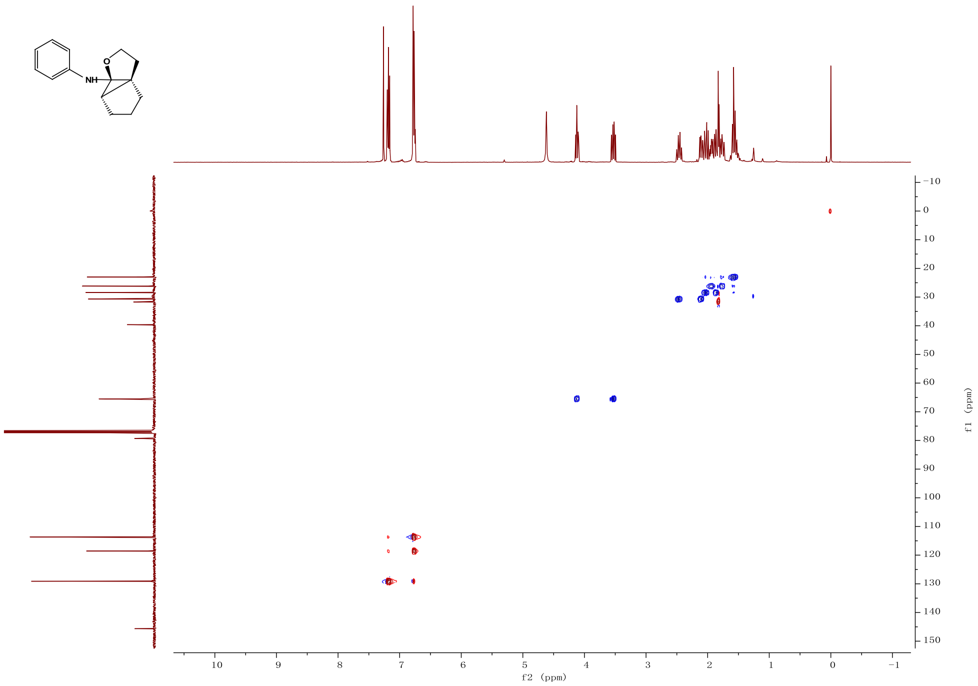

The HSQC spectrum of compound **21**

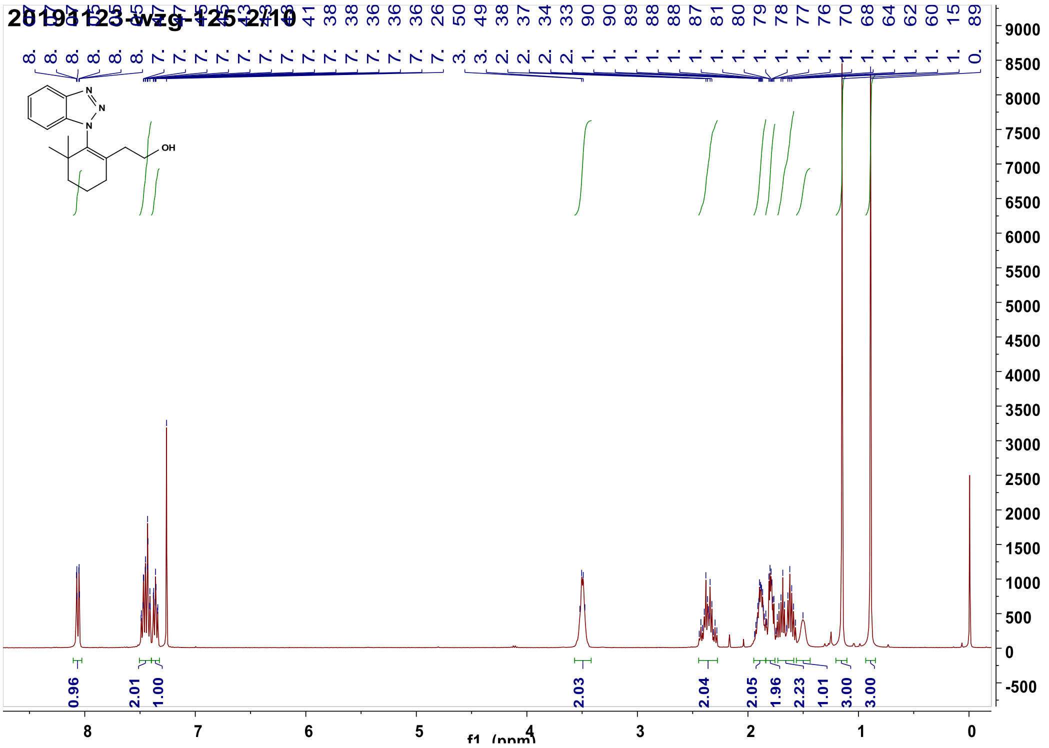

^{1}H NMR spectrum of compound **15c**

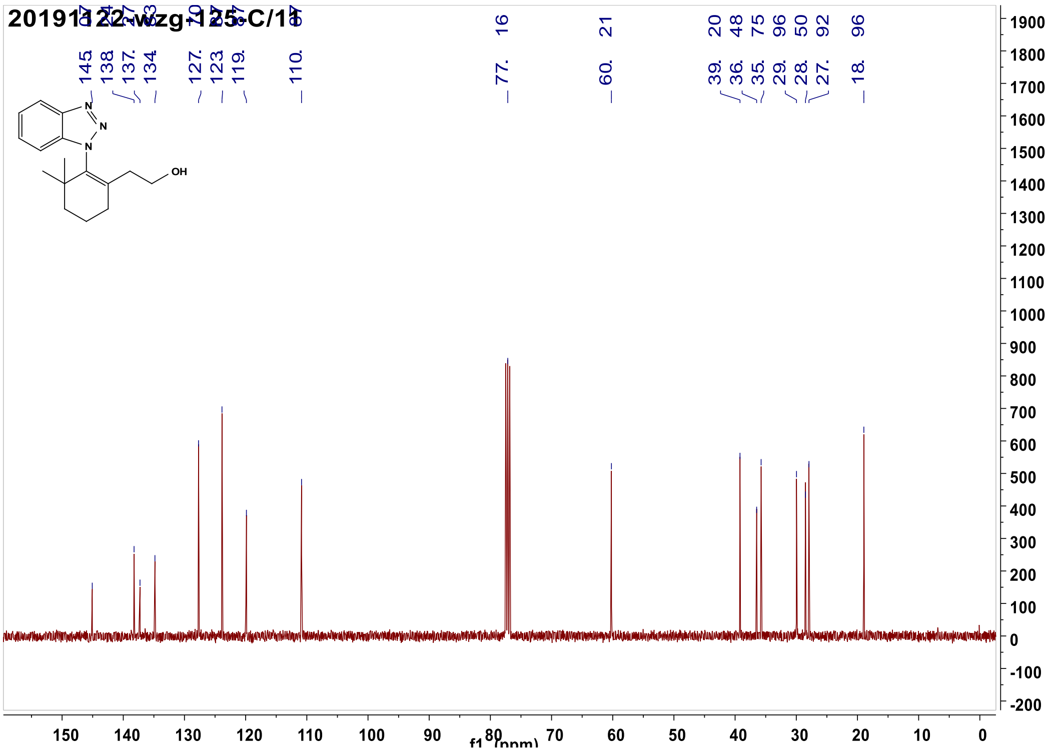

^{13}C NMR spectrum of compound **15c**

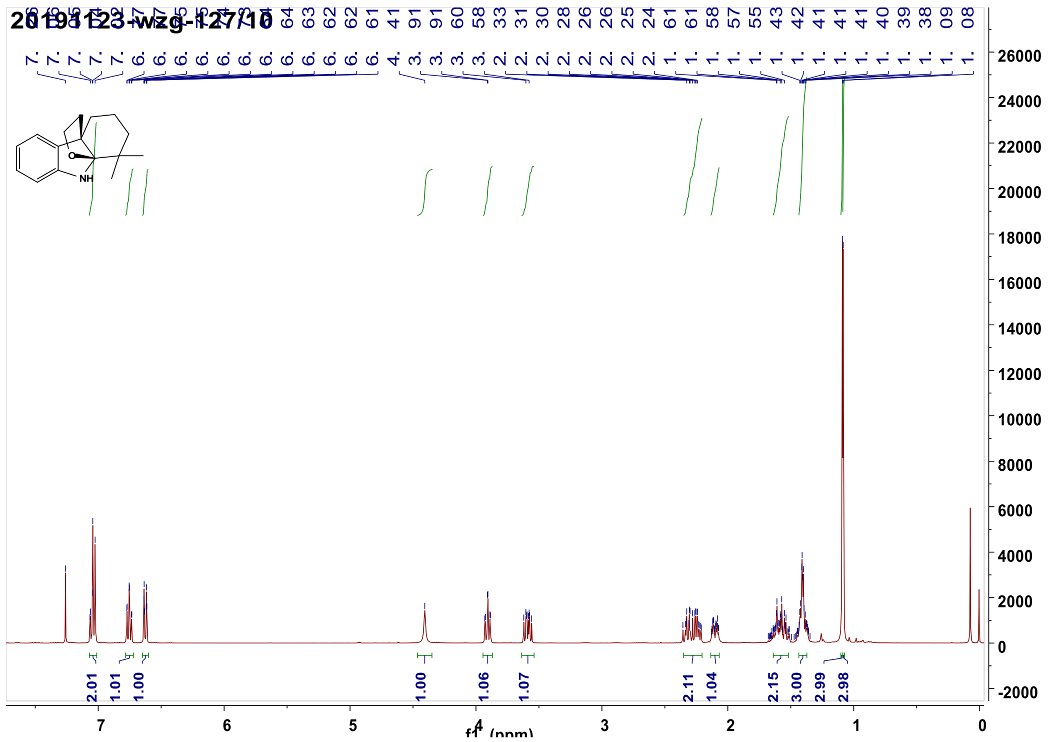

^{1}H NMR spectrum of compound **17c**

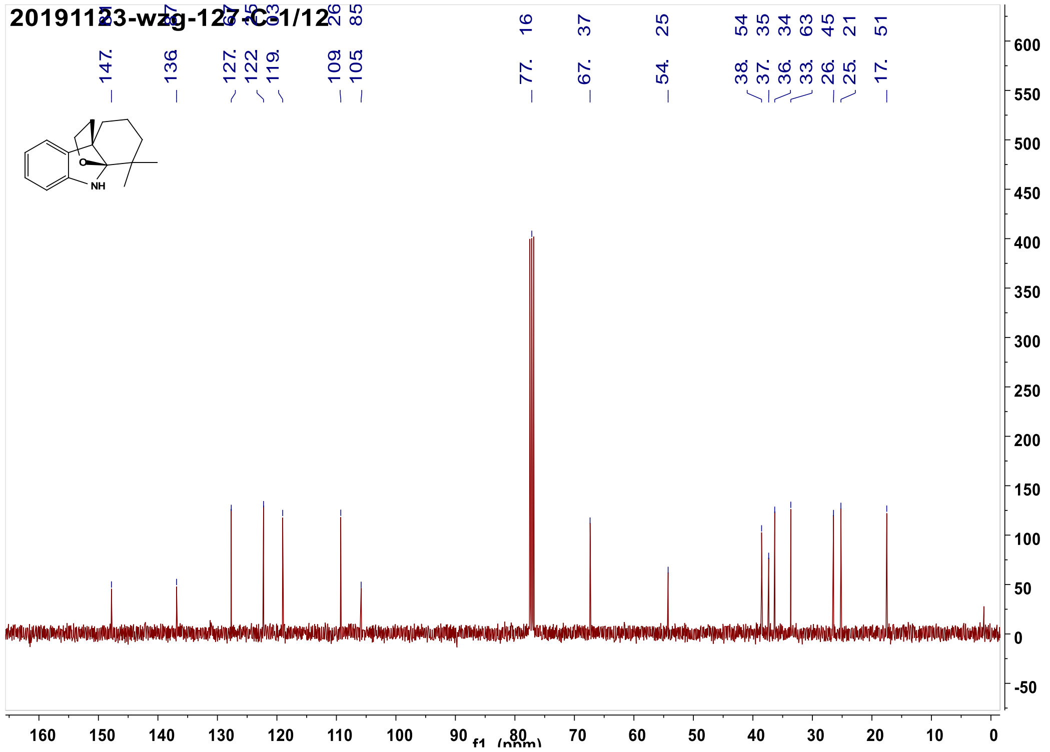

^{13}C NMR spectrum of compound **17c**

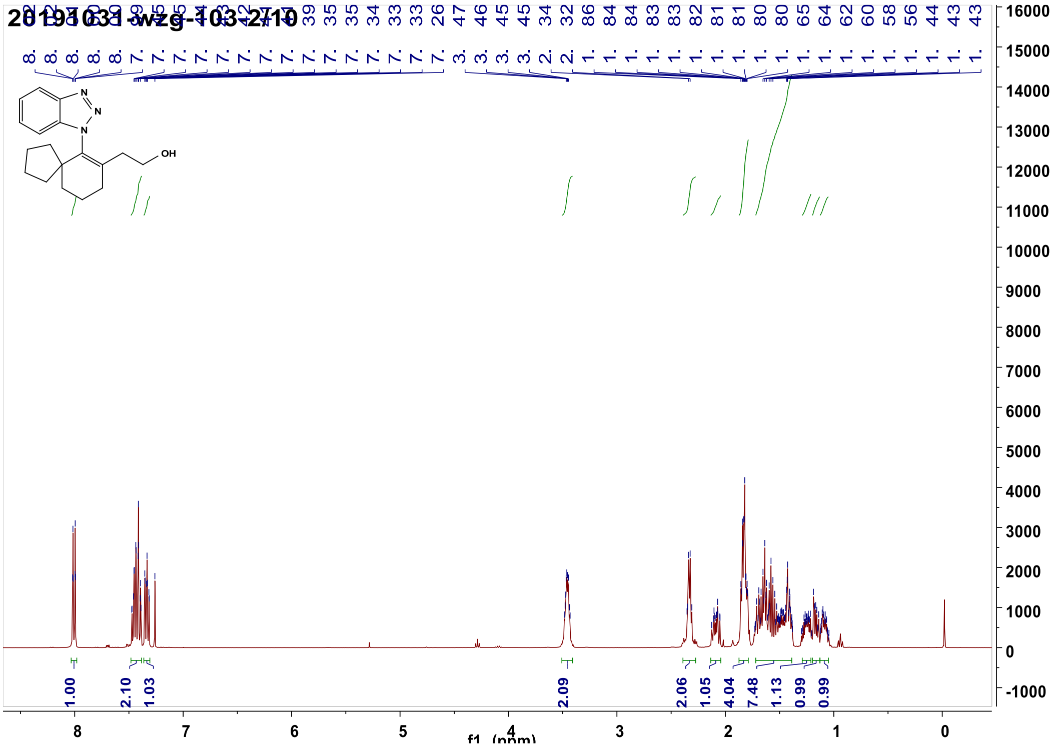

^{1}H NMR spectrum of compound **15d**

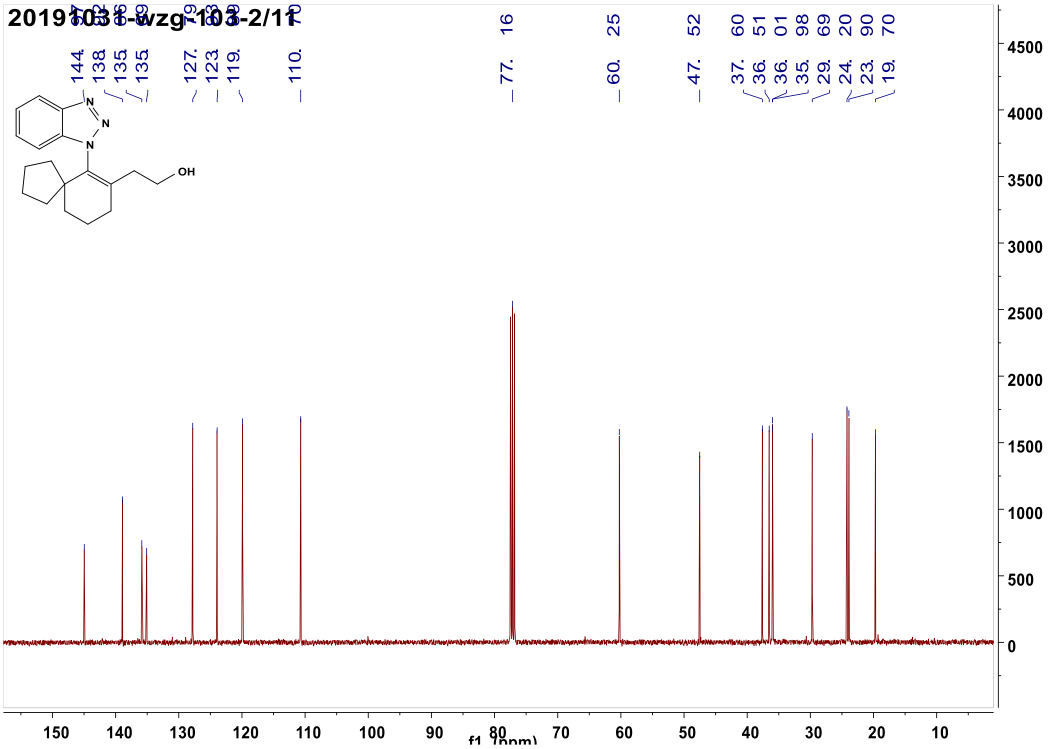

^{13}C NMR spectrum of compound **15d**

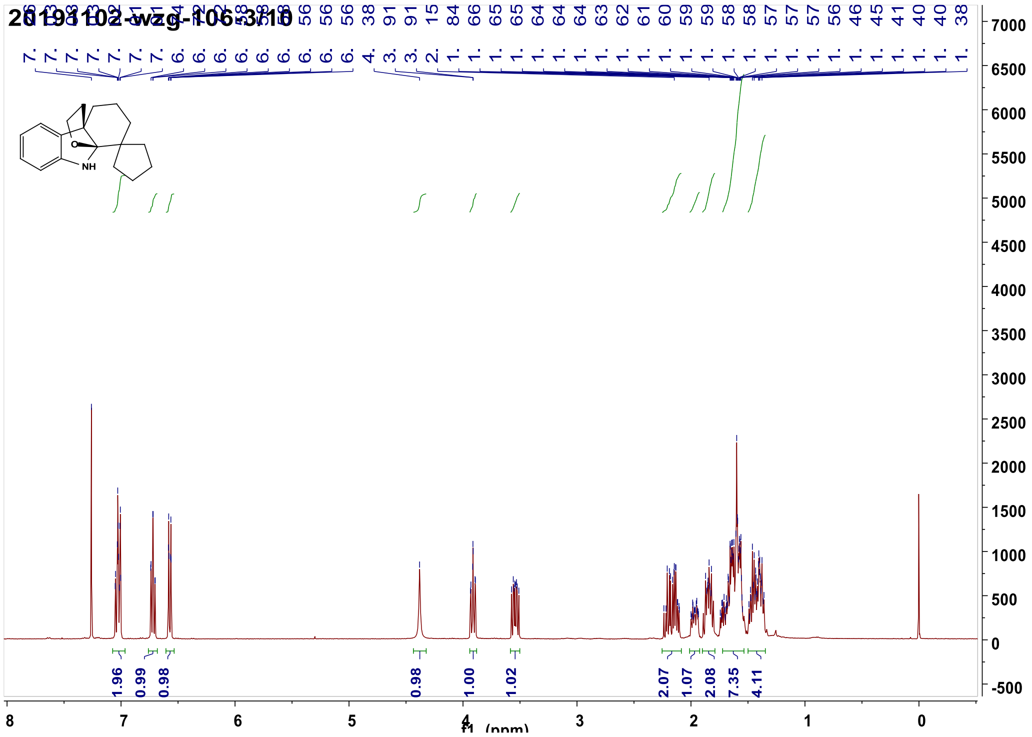

^{1}H NMR spectrum of compound **17d**

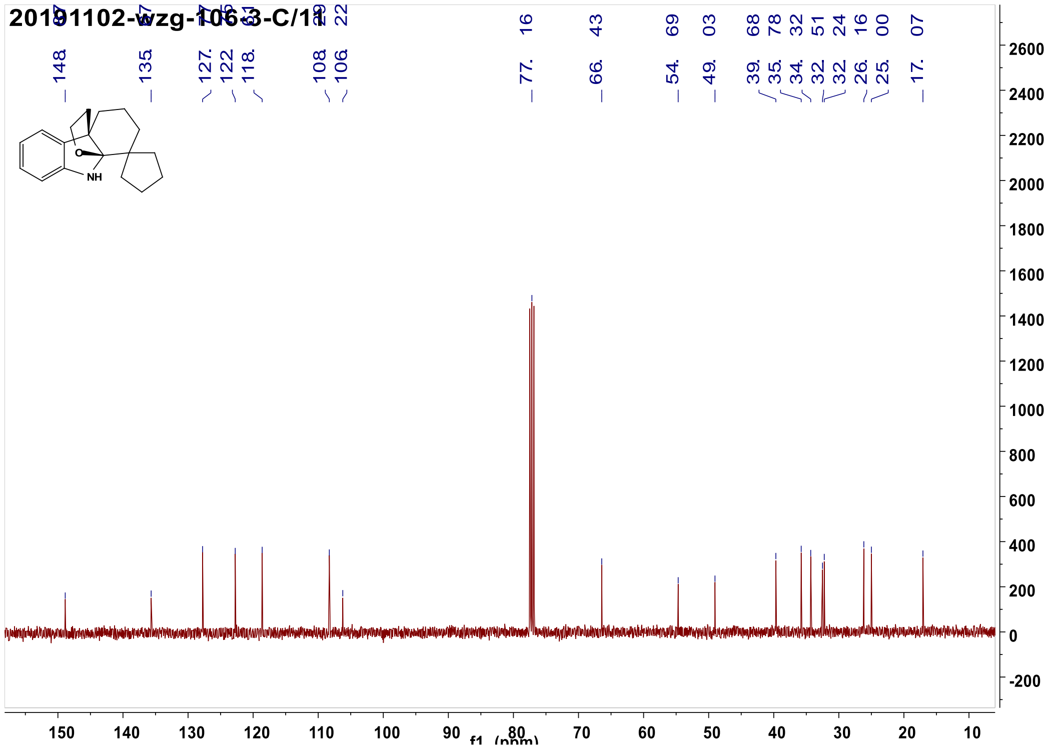

^{13}C NMR spectrum of compound **17d**

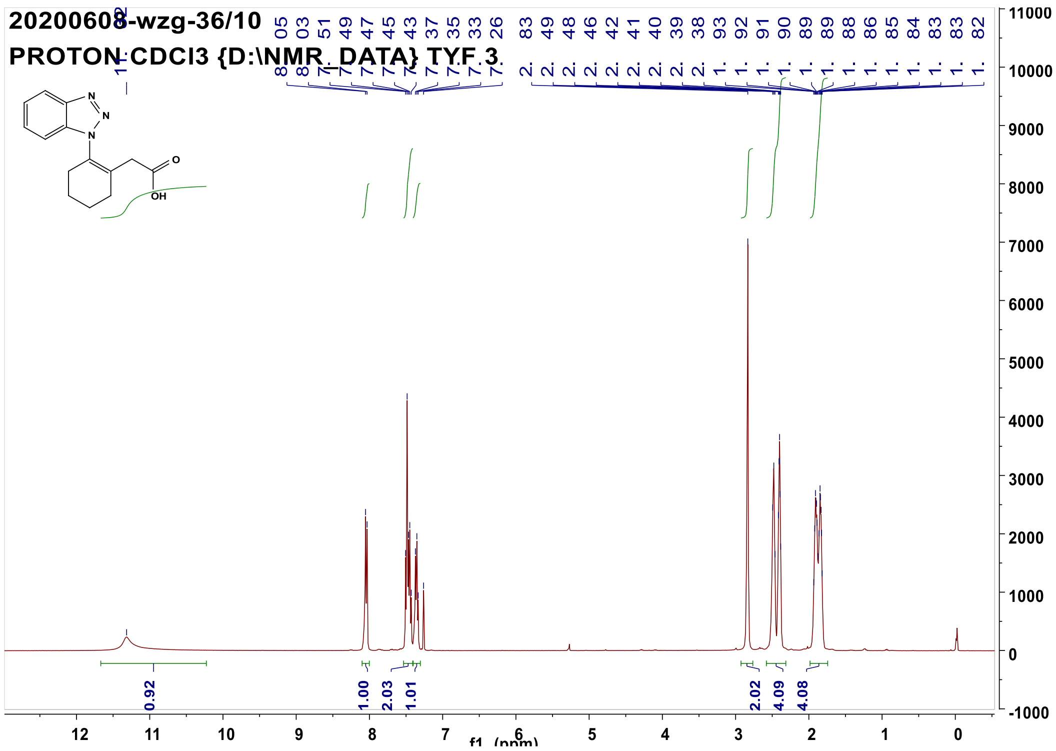

^{1}H NMR spectrum of compound **15e**

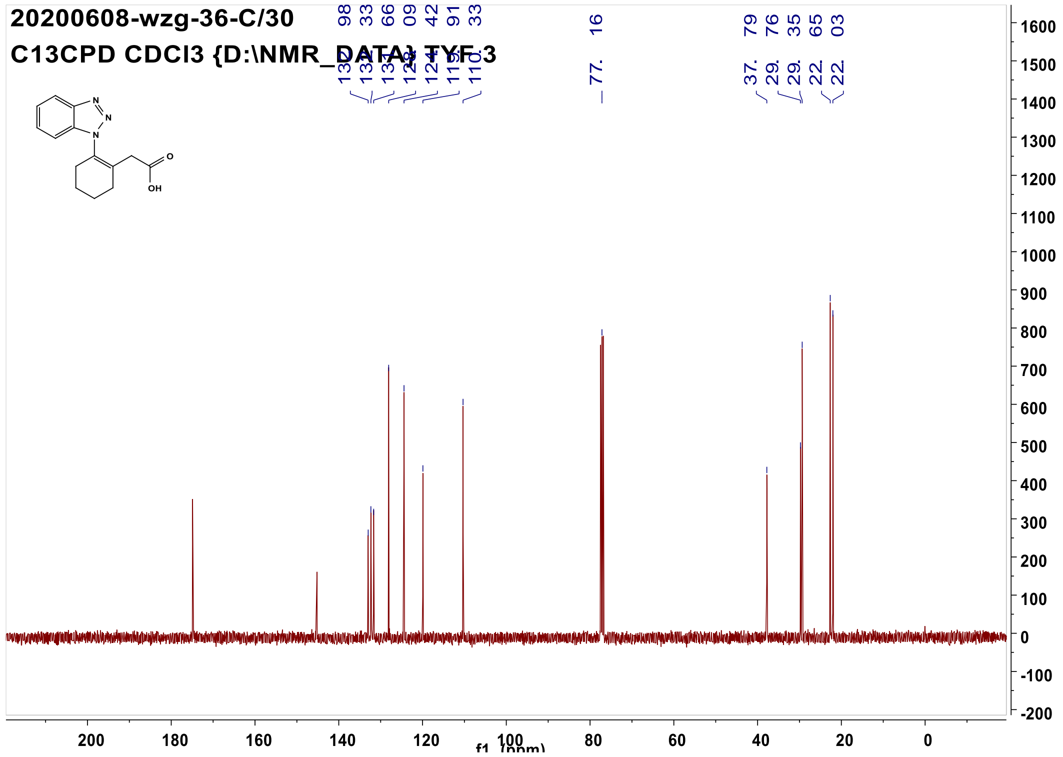

^{13}C NMR spectrum of compound **15e**

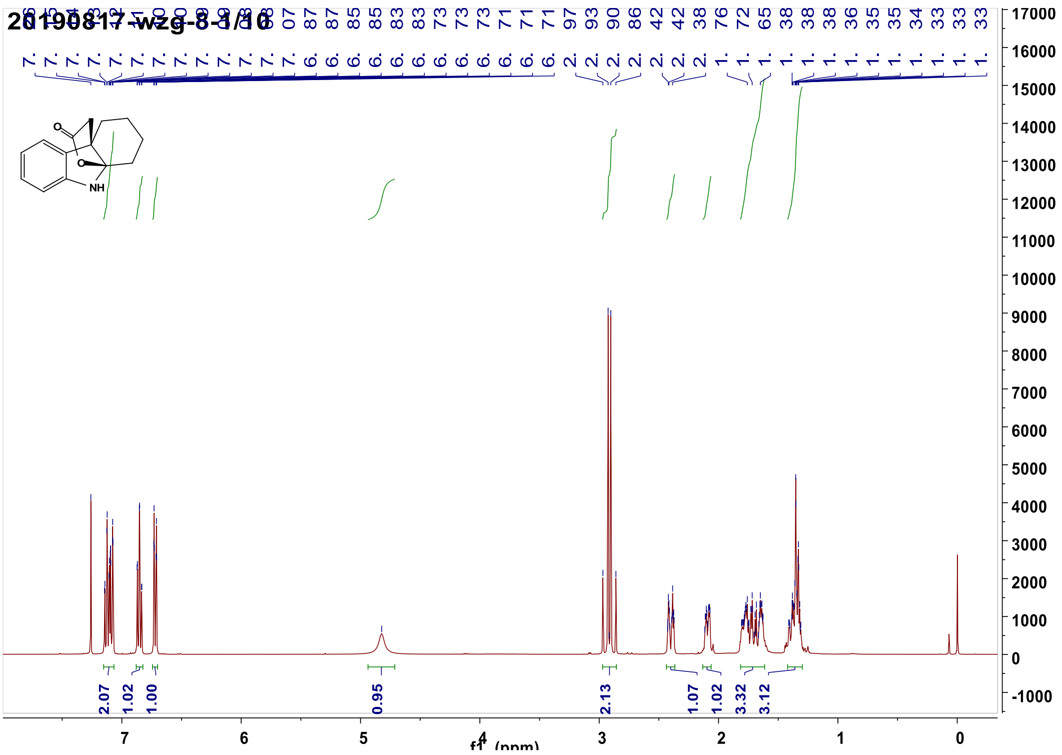

^{1}H NMR spectrum of compound **17e**

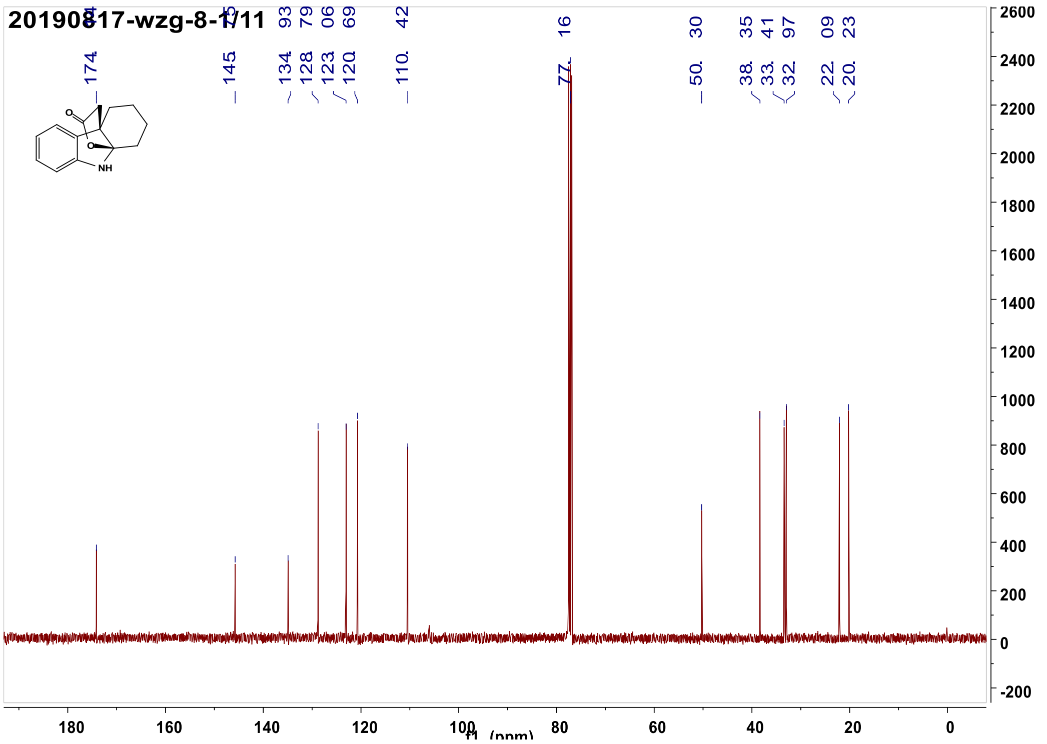

^{13}C NMR spectrum of compound **17e**

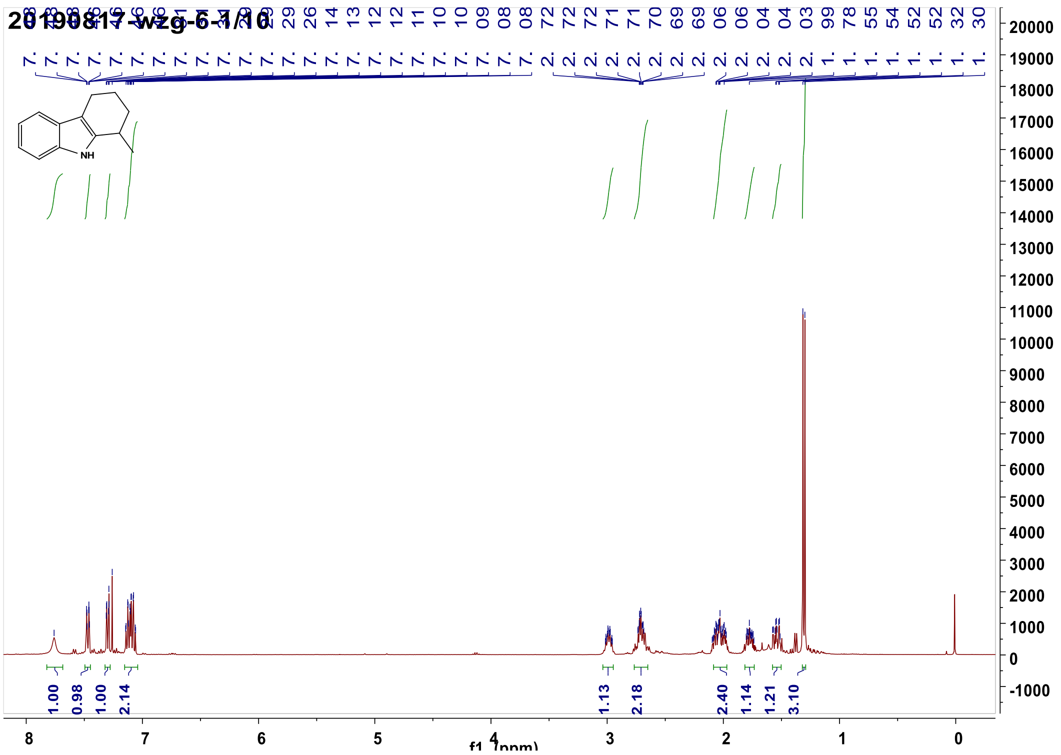

^{1}H NMR spectrum of compound **22**

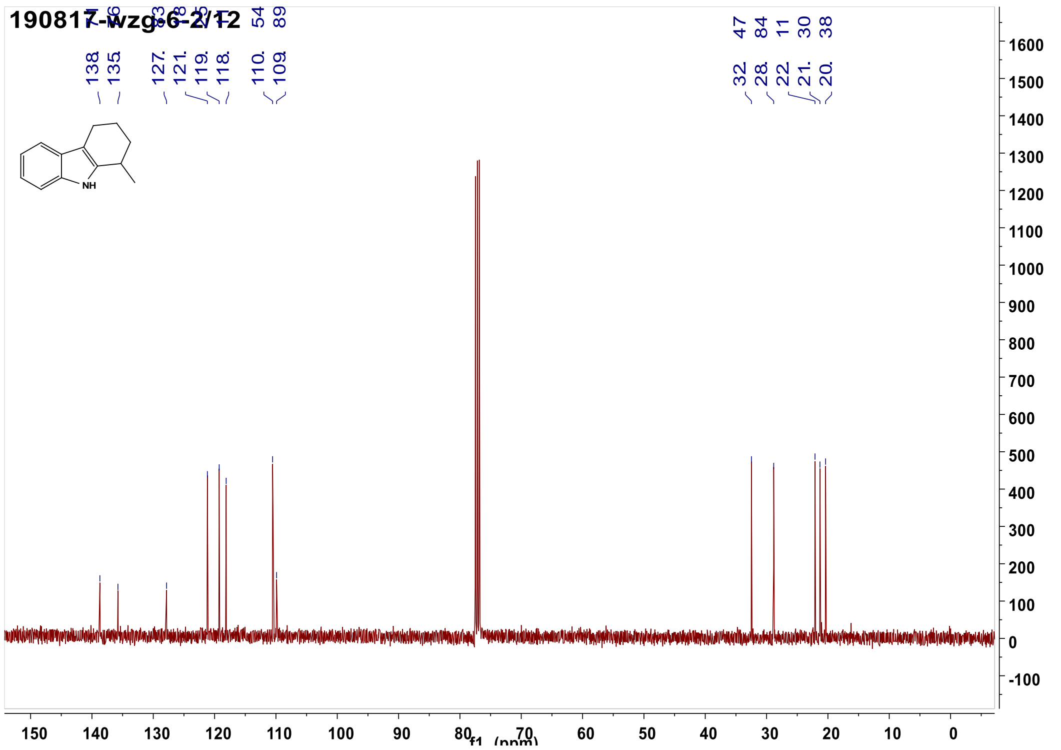

^{13}C NMR spectrum of compound **22**

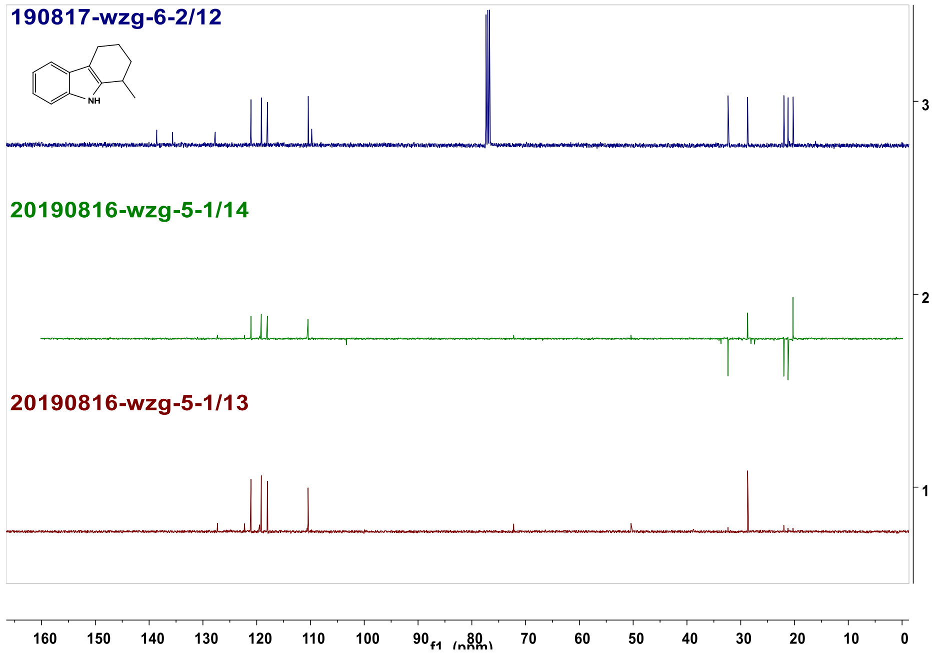

The DEPT90^{o} and 135° spectrum of compound **22**

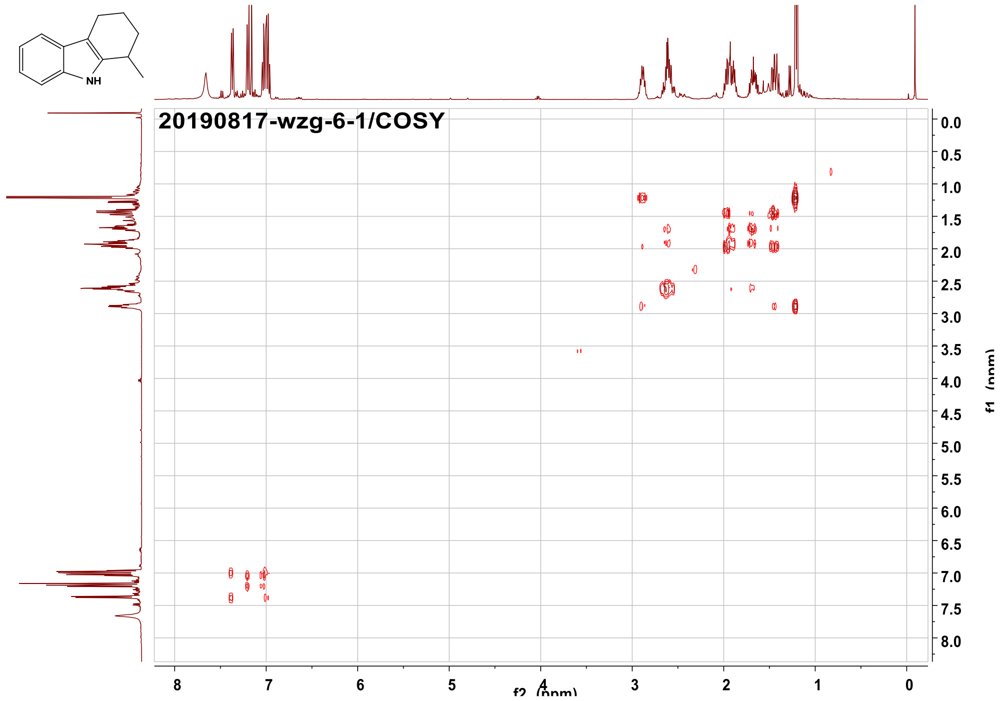

The COSY spectrum of compound **22**

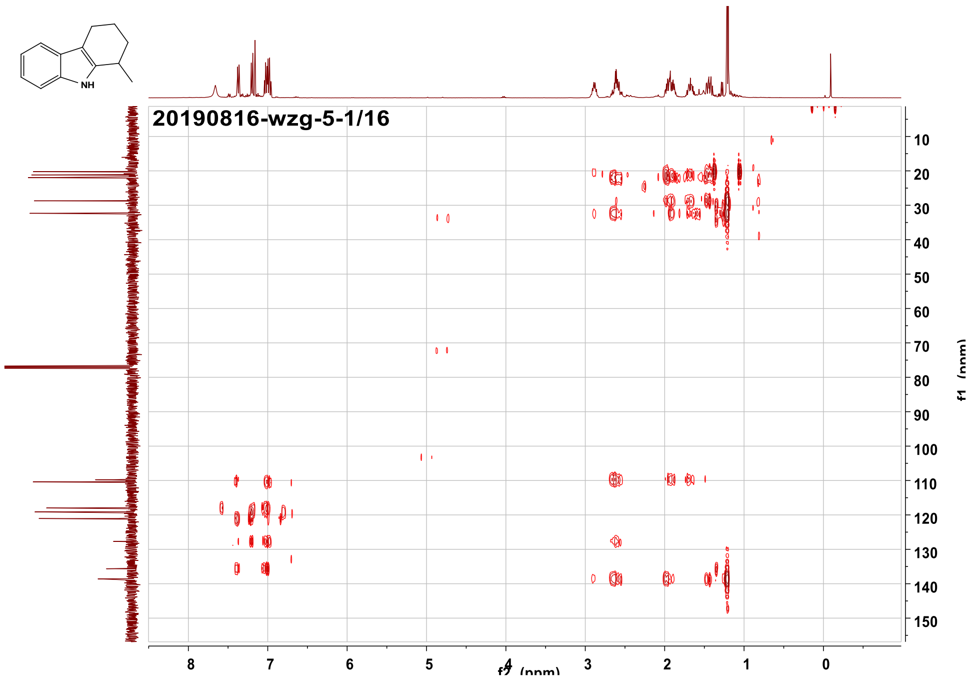

The HMBC spectrum of compound **22**

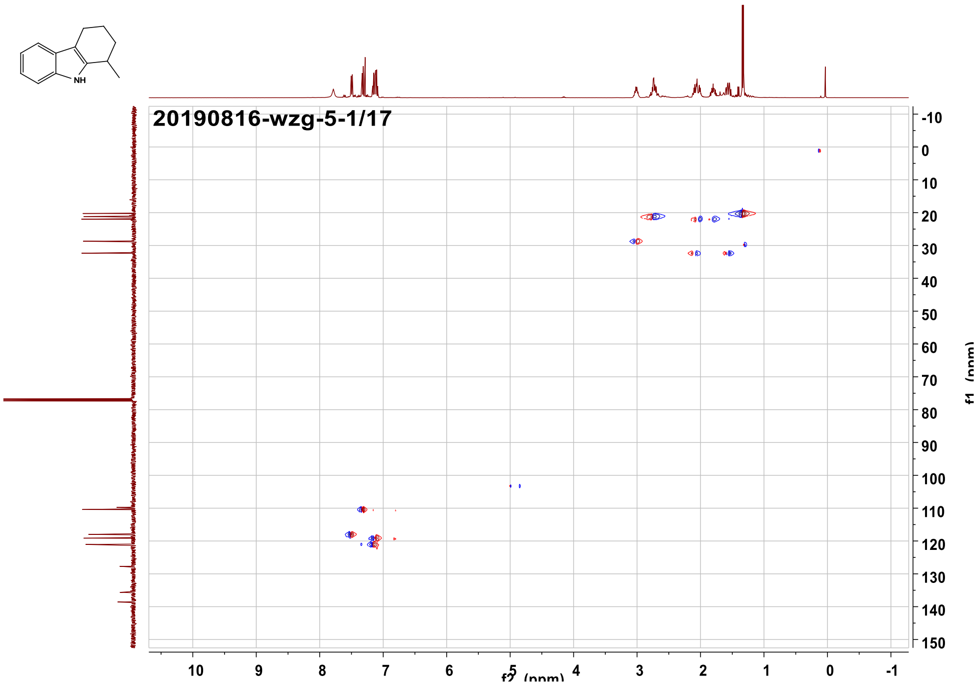

The HSQC spectrum of compound **22**

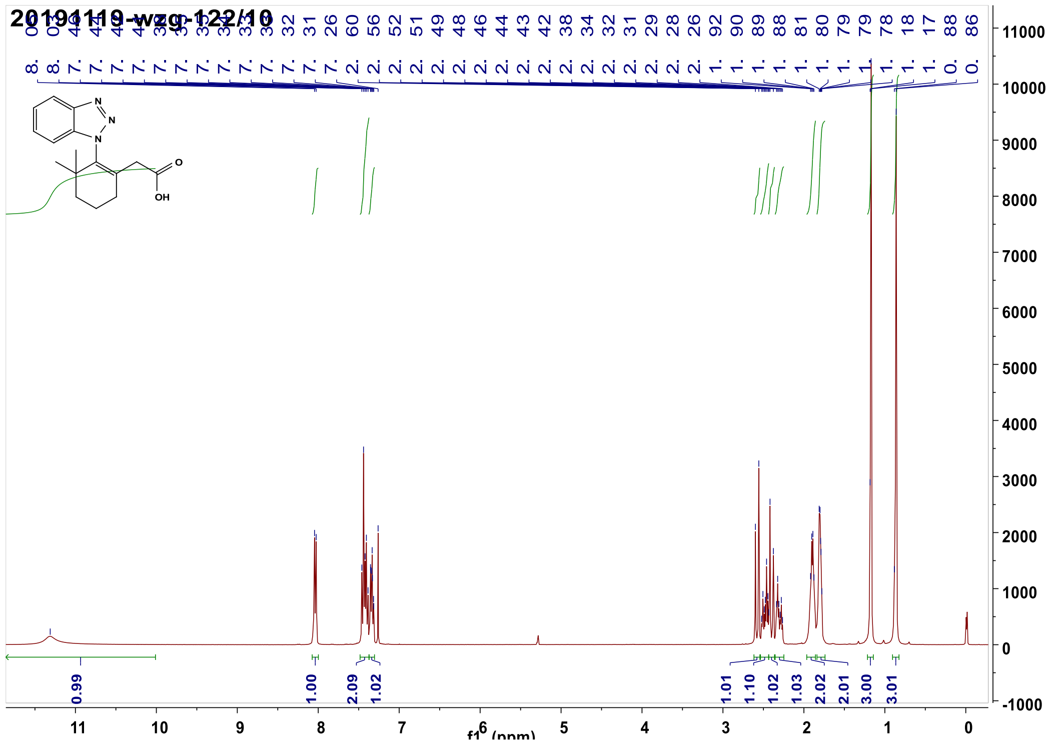

^{1}H NMR spectrum of compound **15f**

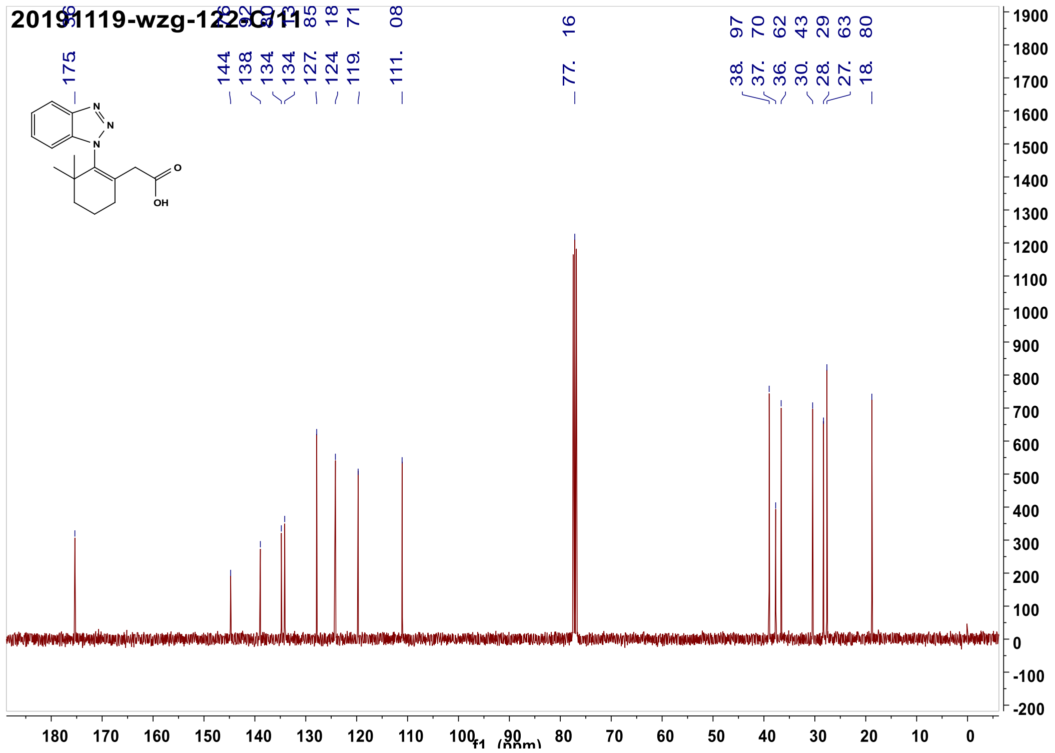

^{13}C NMR spectrum of compound **15f**

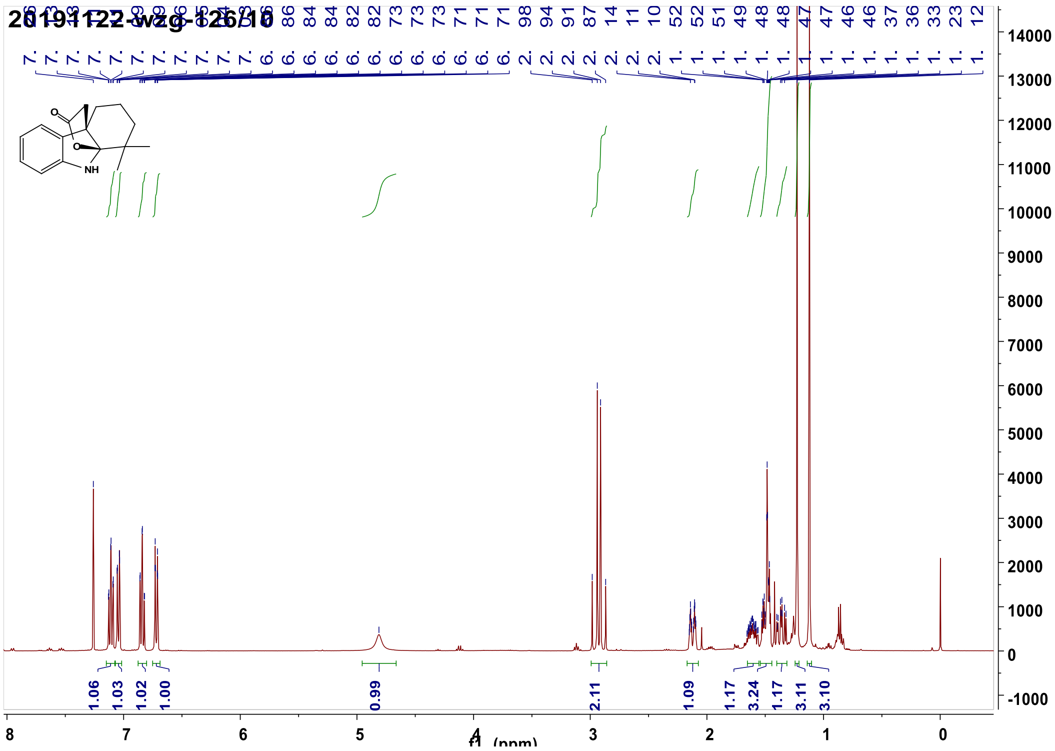

^{1}H NMR spectrum of compound **17f**

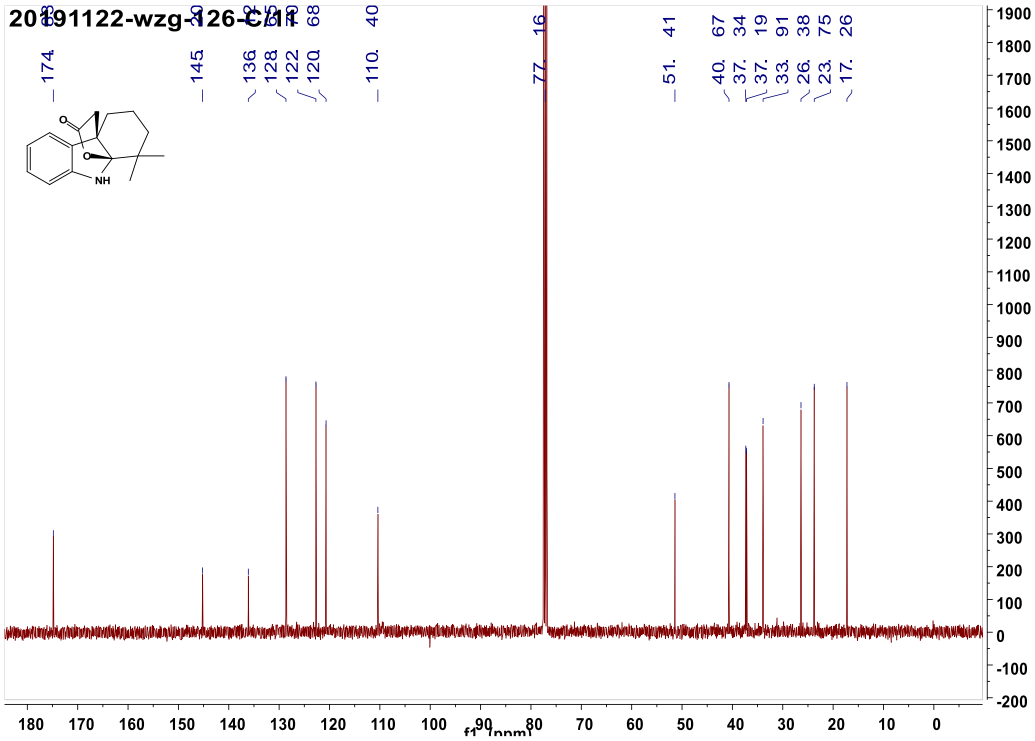

^{13}C NMR spectrum of compound **17f**

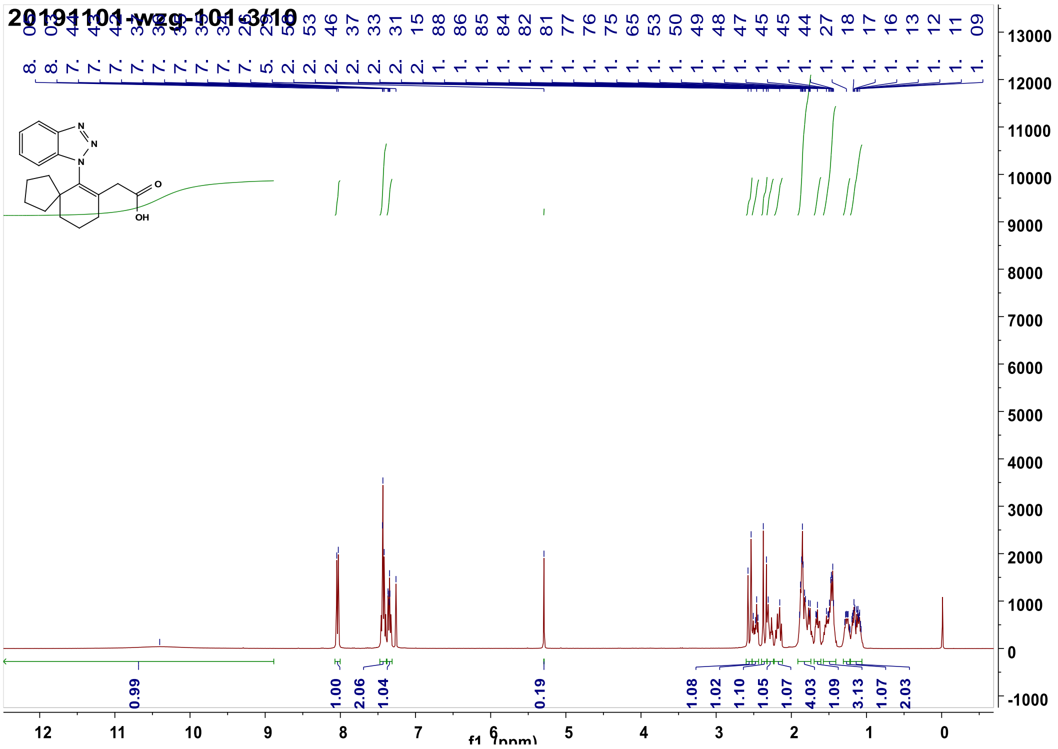

^{1}H NMR spectrum of compound **15g**

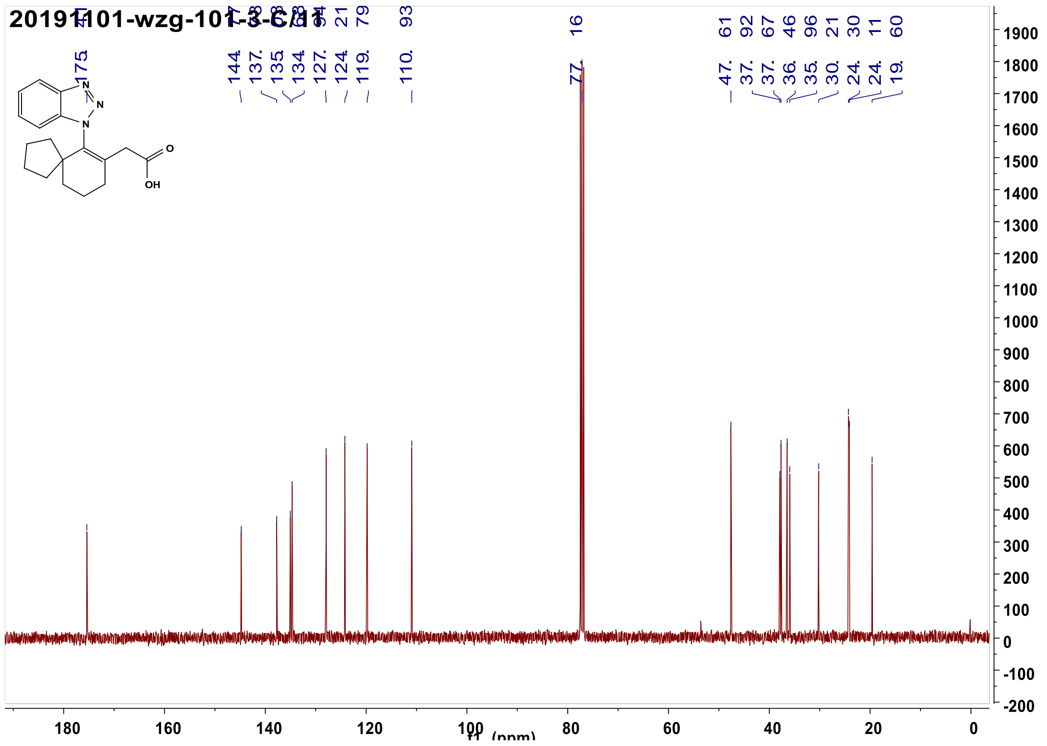

^{13}C NMR spectrum of compound **15g**

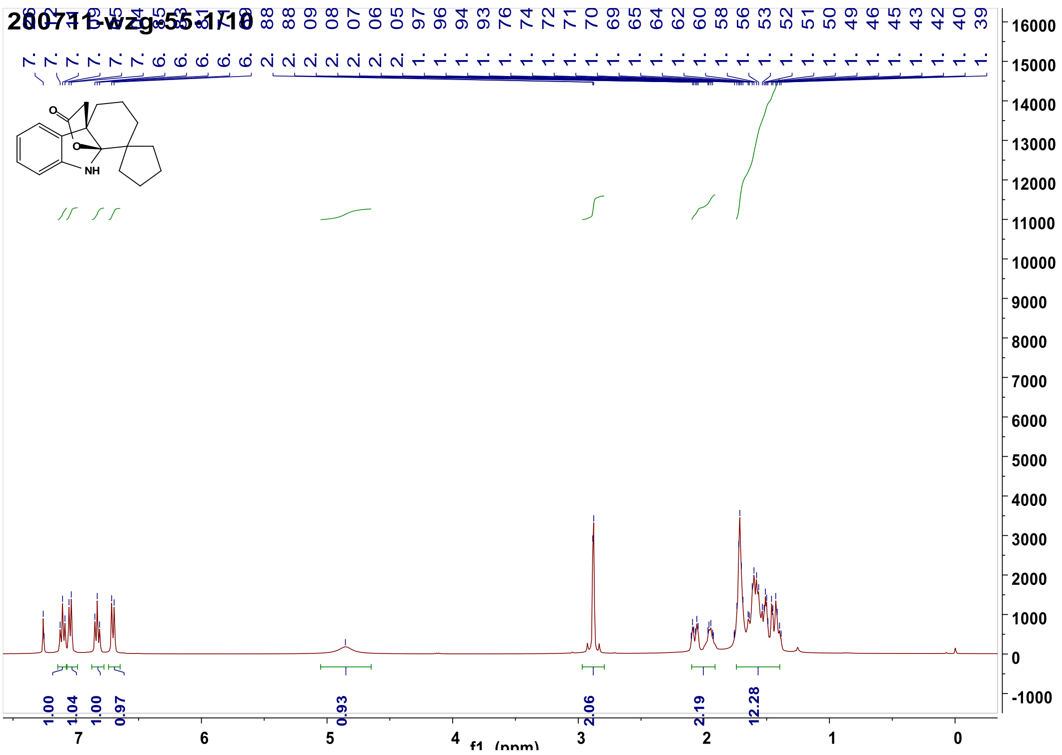

^{1}H NMR spectrum of compound **17g**

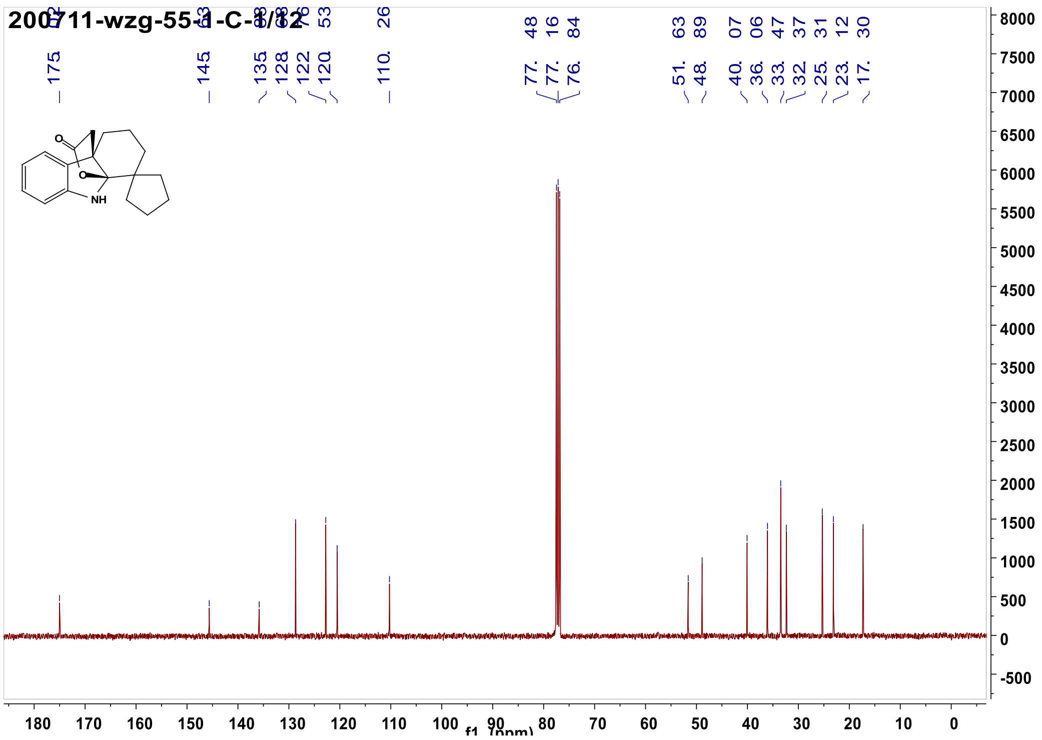

^{13}C NMR spectrum of compound **17g**

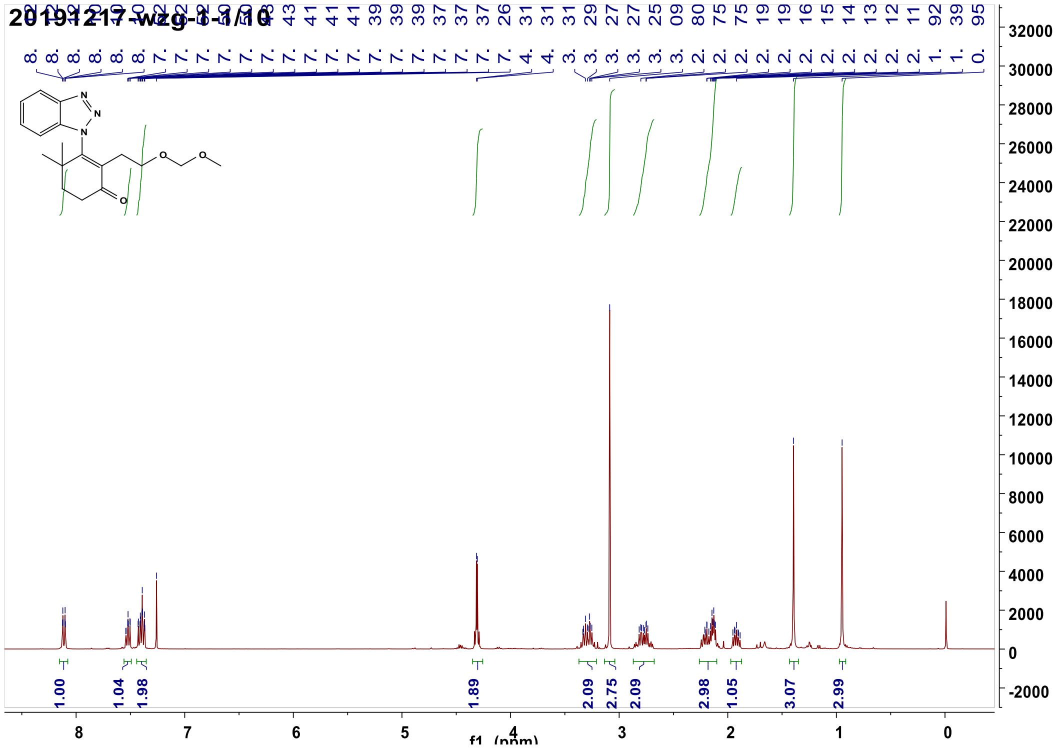

^{1}H NMR spectrum of compound **15h**

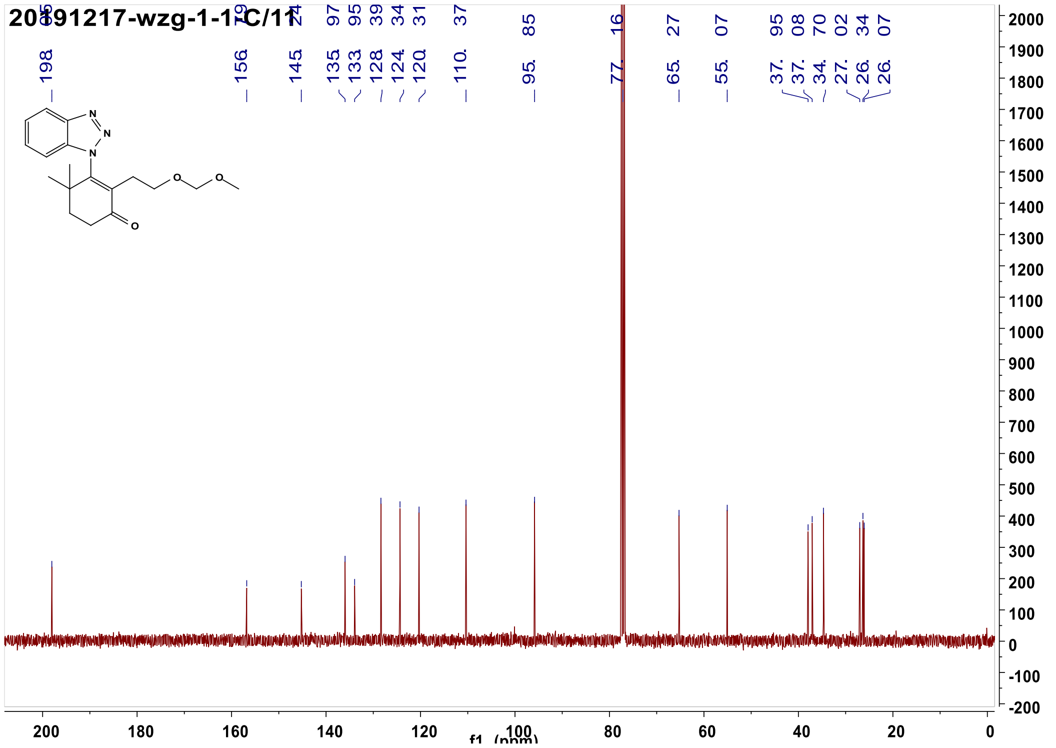

^{13}C NMR spectrum of compound **15h**

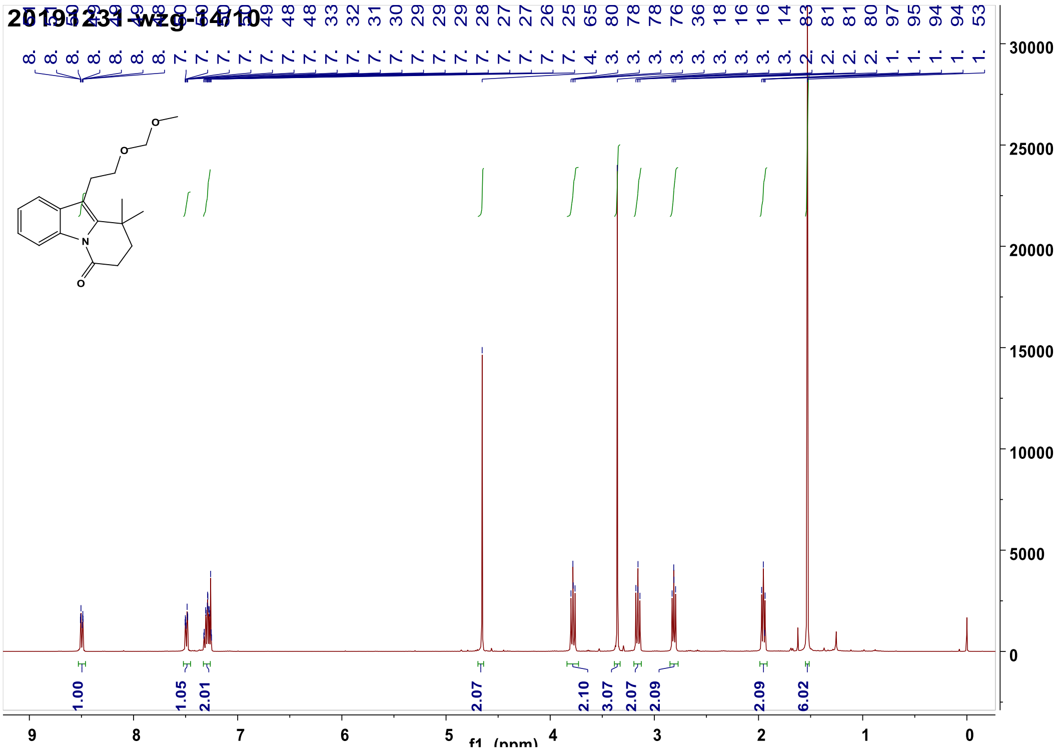

^{1}H NMR spectrum of compound **20h**

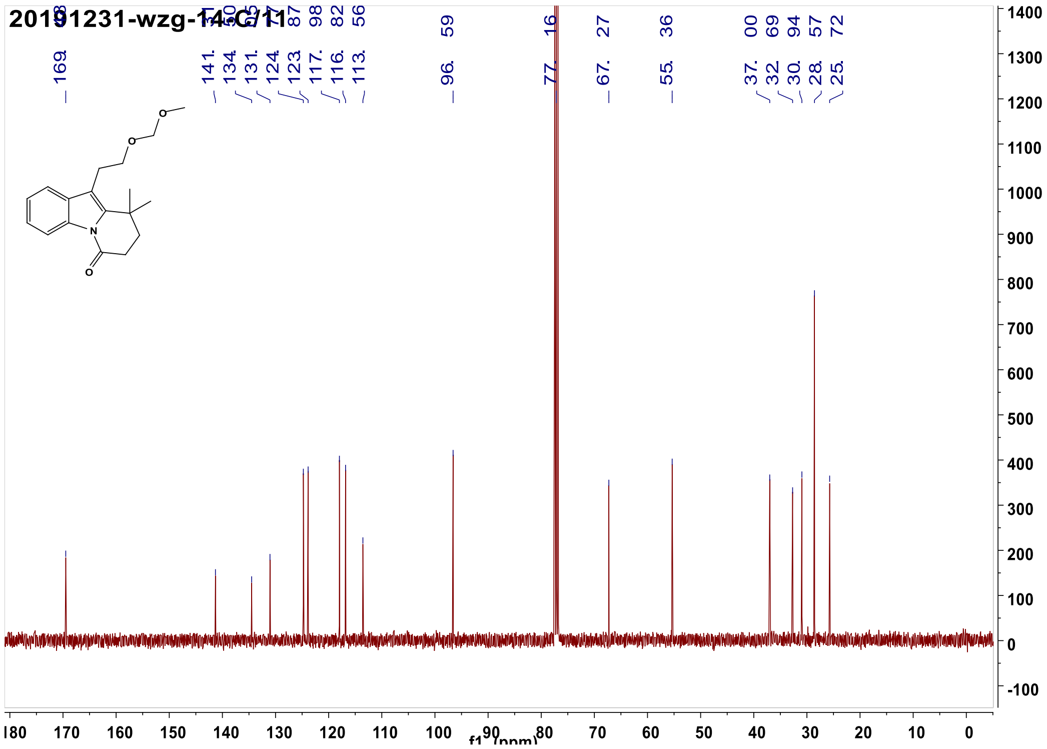

^{13}C NMR spectrum of compound **20h**

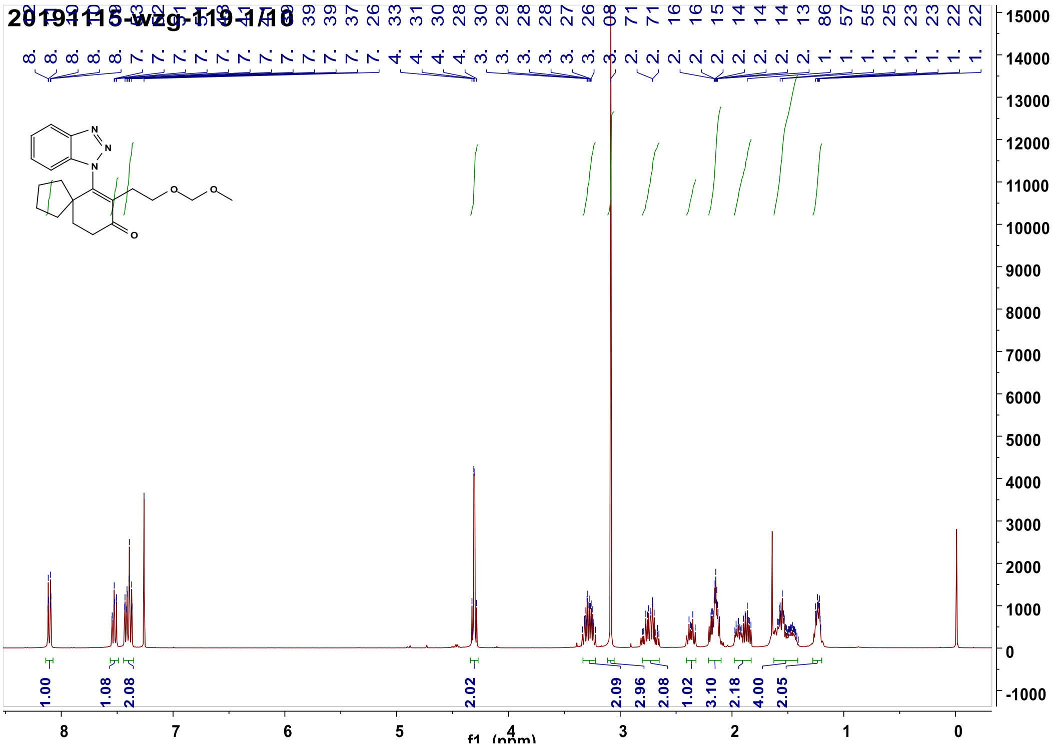

^{1}H NMR spectrum of compound **15i**

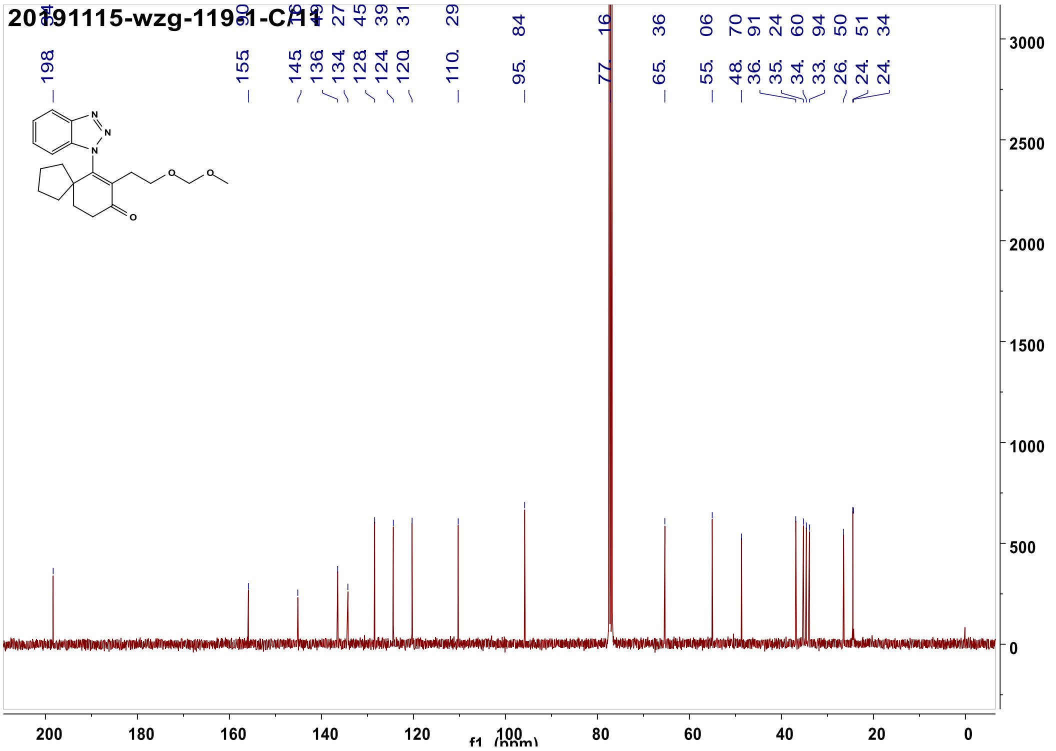

^{13}C NMR spectrum of compound **15i**

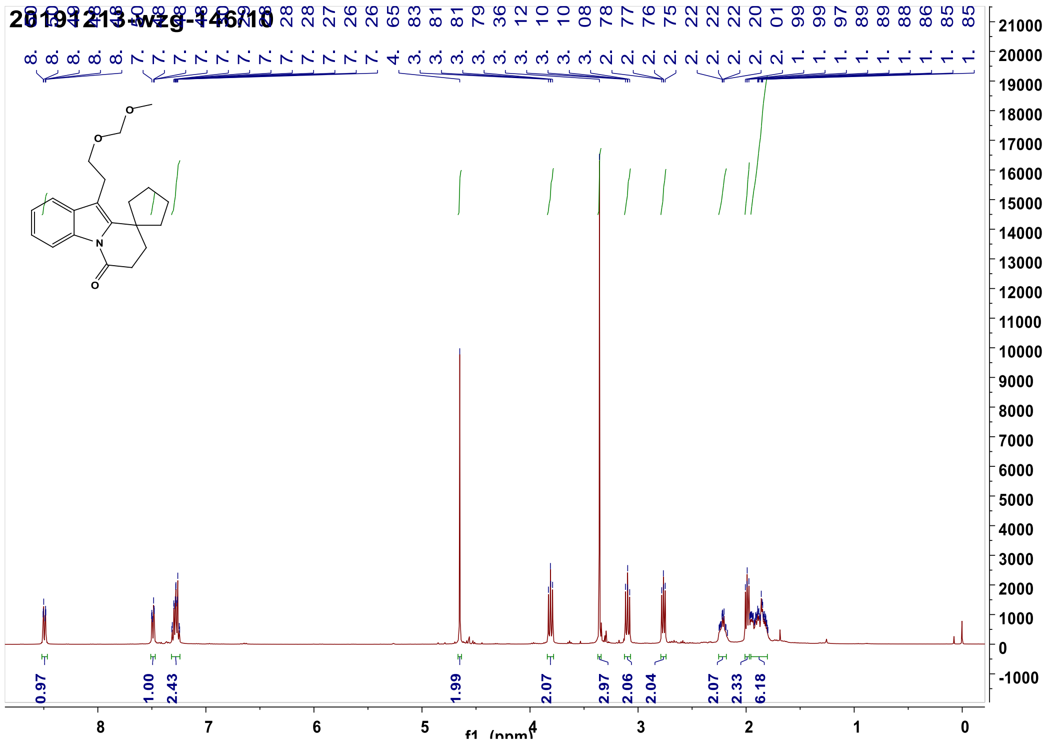

^{1}H NMR spectrum of compound **20i**

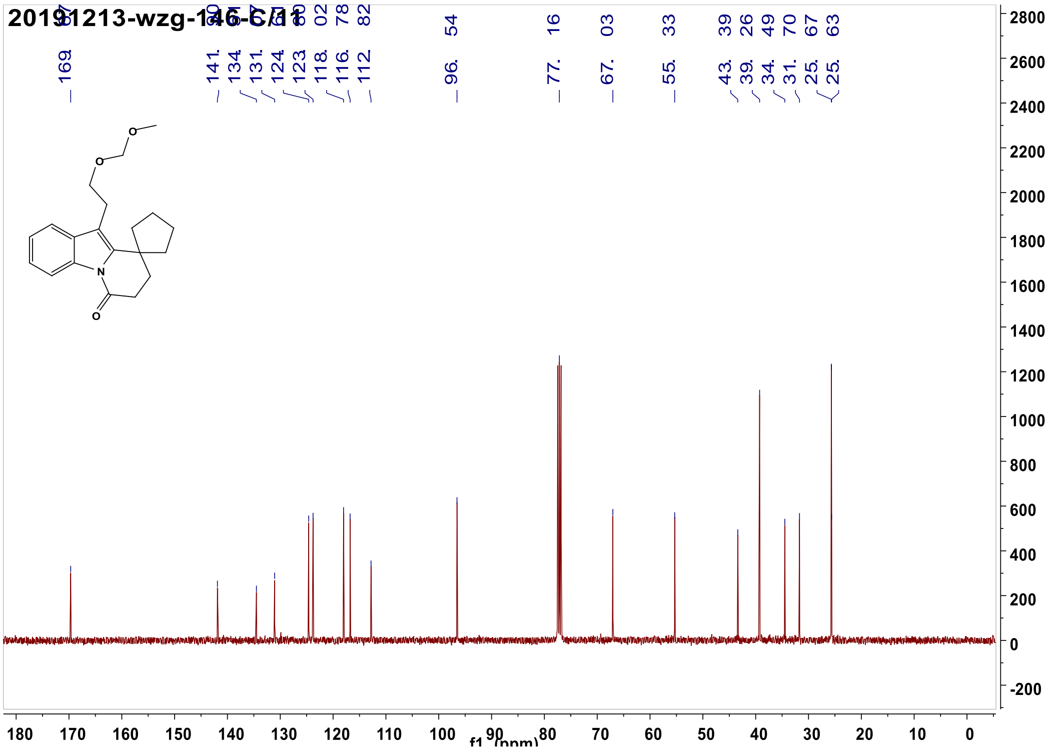

^{13}C NMR spectrum of compound **20i**

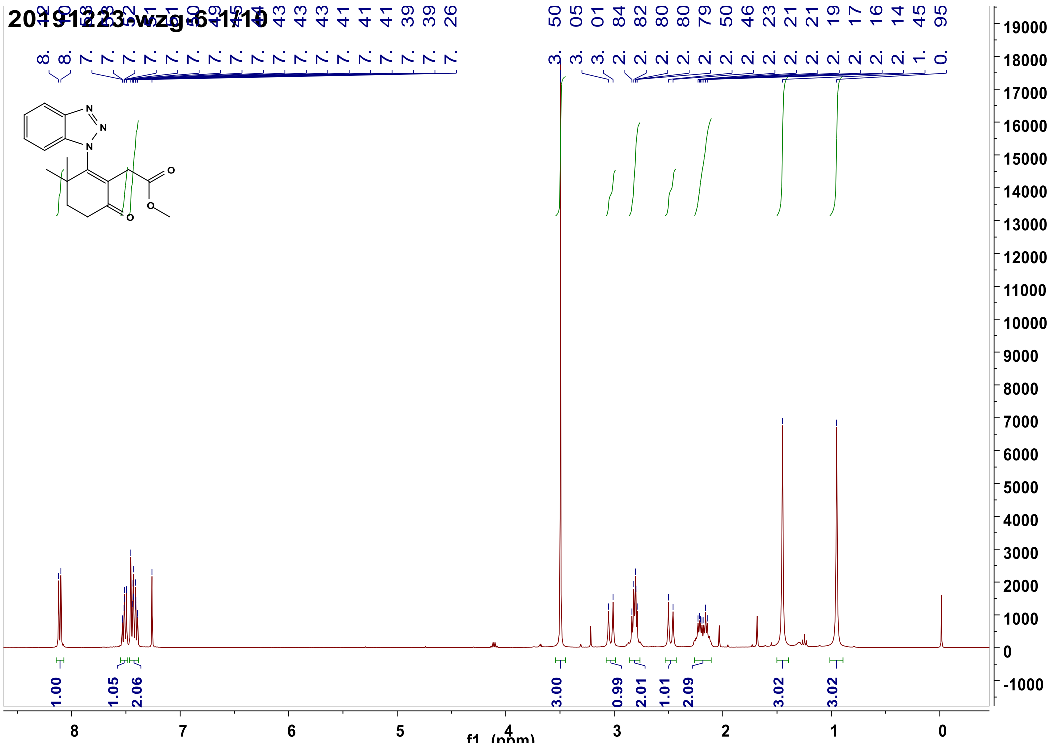

^{1}H NMR spectrum of compound **15j**

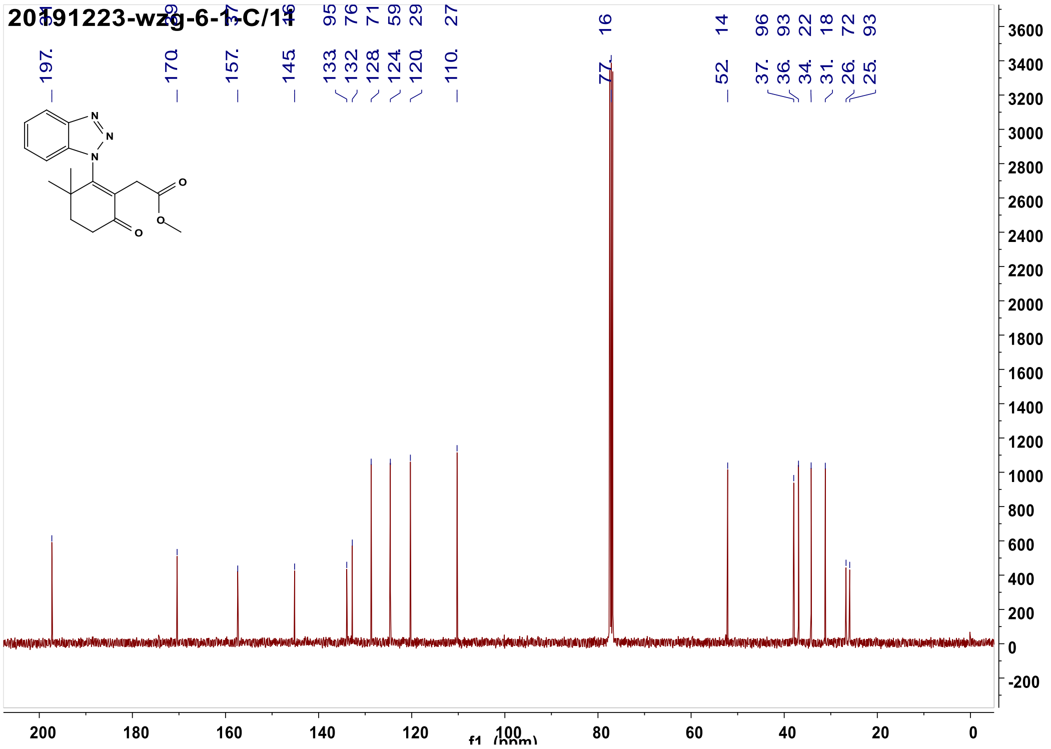

^{13}C NMR spectrum of compound **15j**

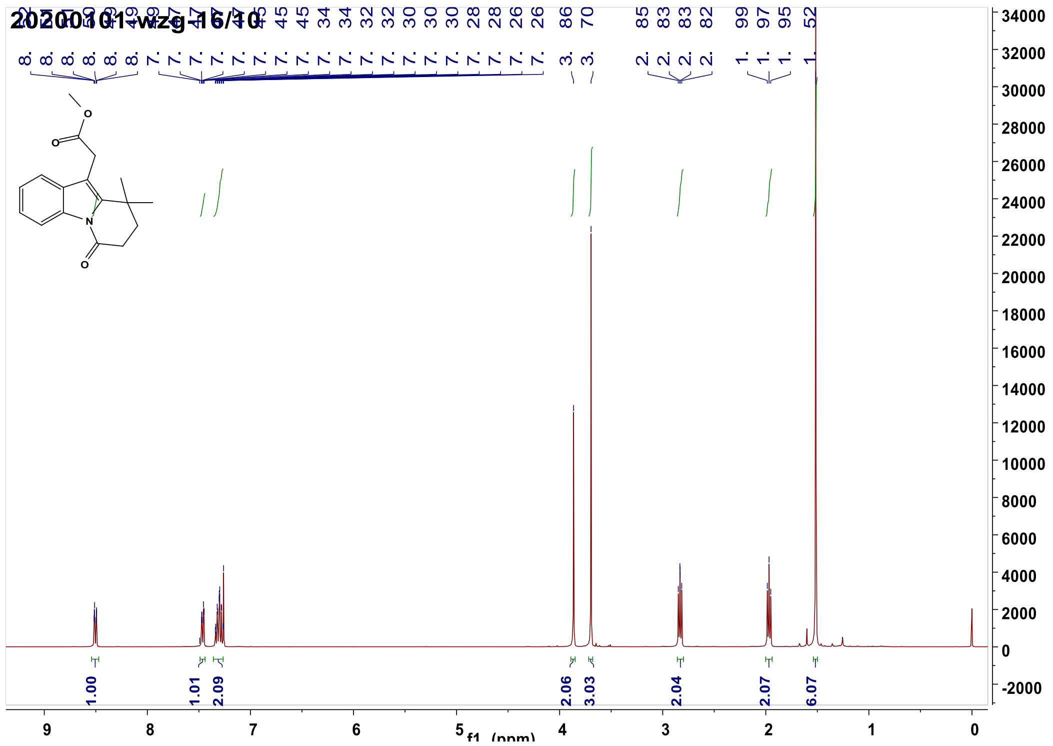

^{1}H NMR spectrum of compound **20j**

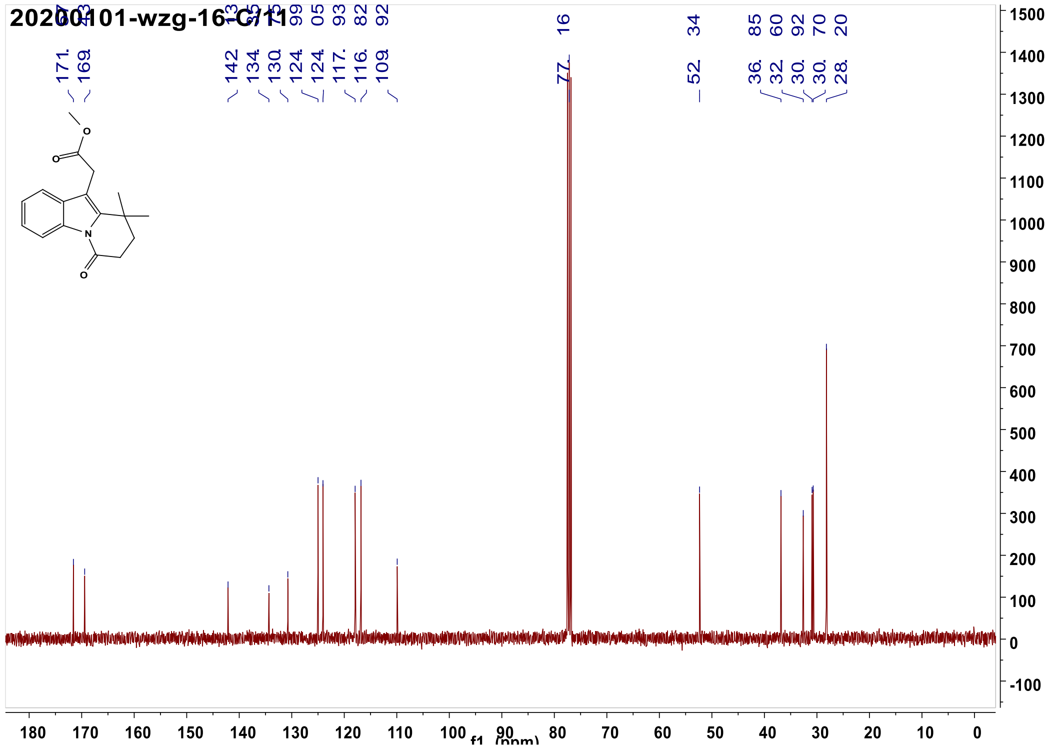

^{13}C NMR spectrum of compound **20j**

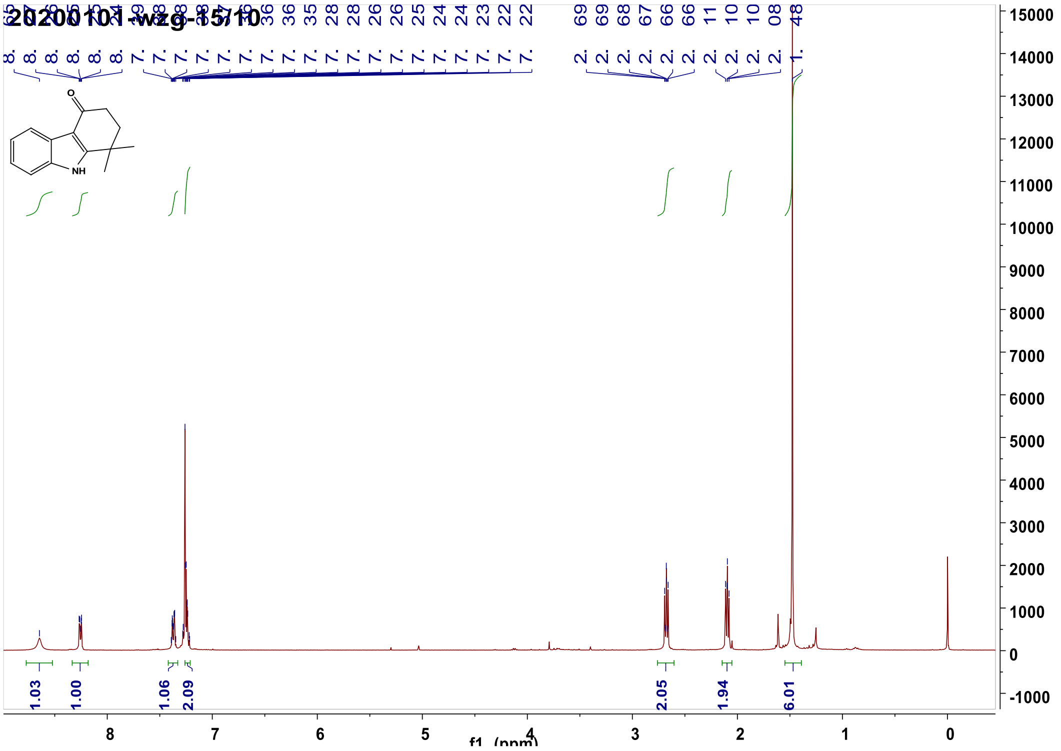

^{1}H NMR spectrum of compound **23**

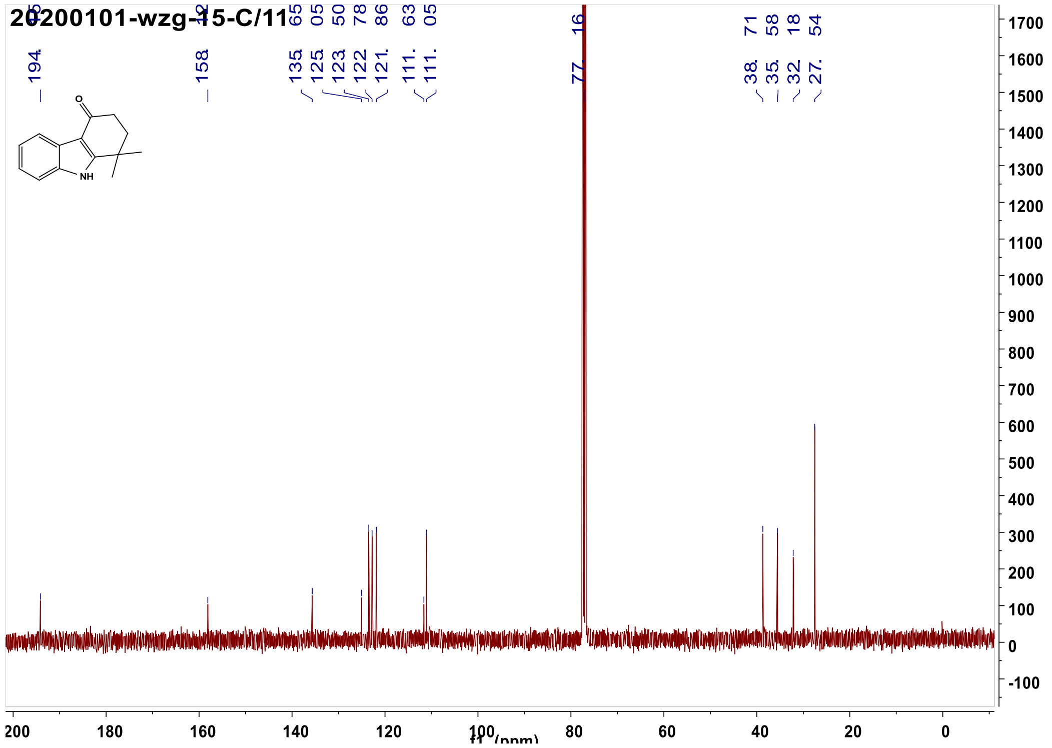

^{13}C NMR spectrum of compound **23**

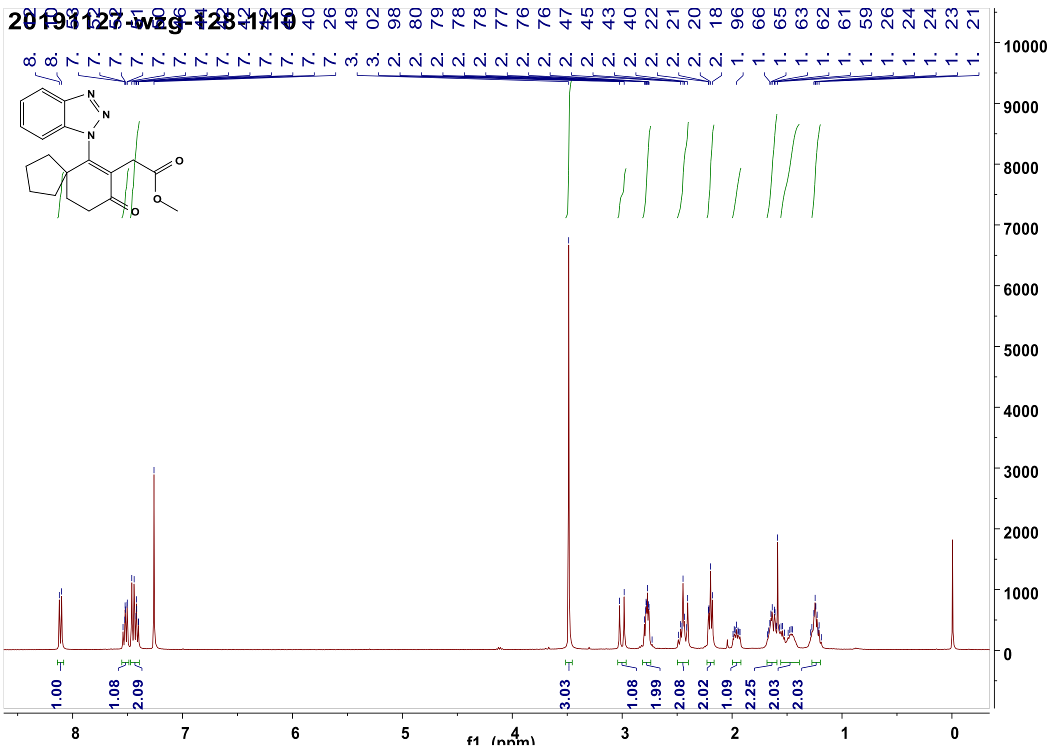

^{1}H NMR spectrum of compound **15k**

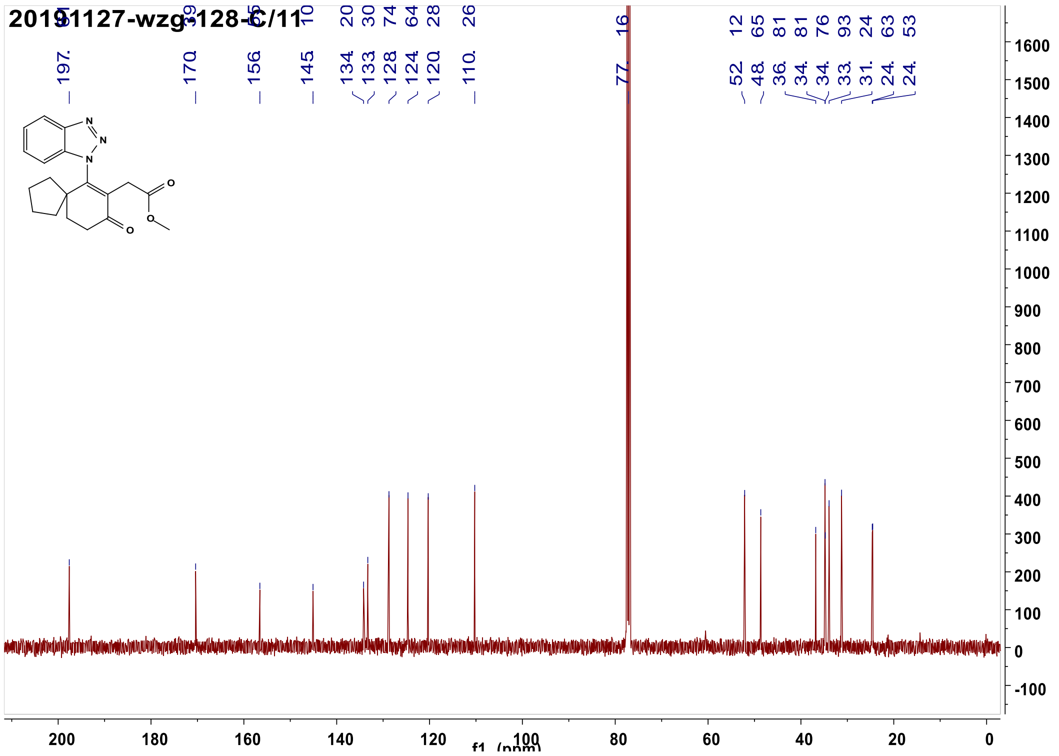

^{13}C NMR spectrum of compound **15k**

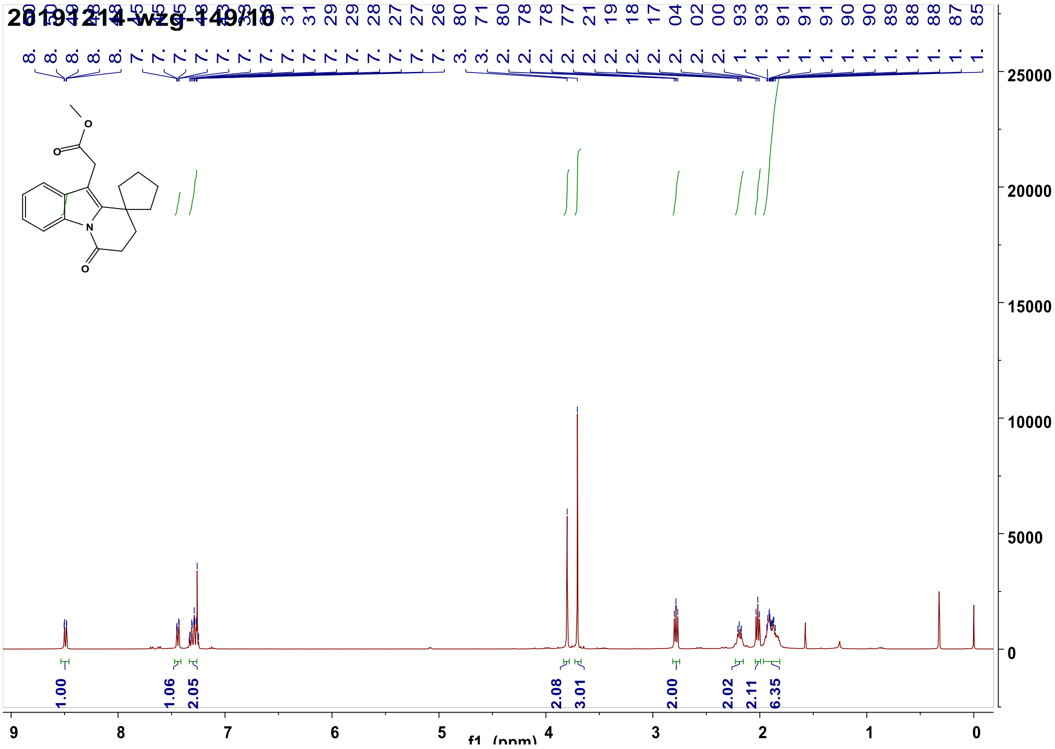

^{1}H NMR spectrum of compound **20k**

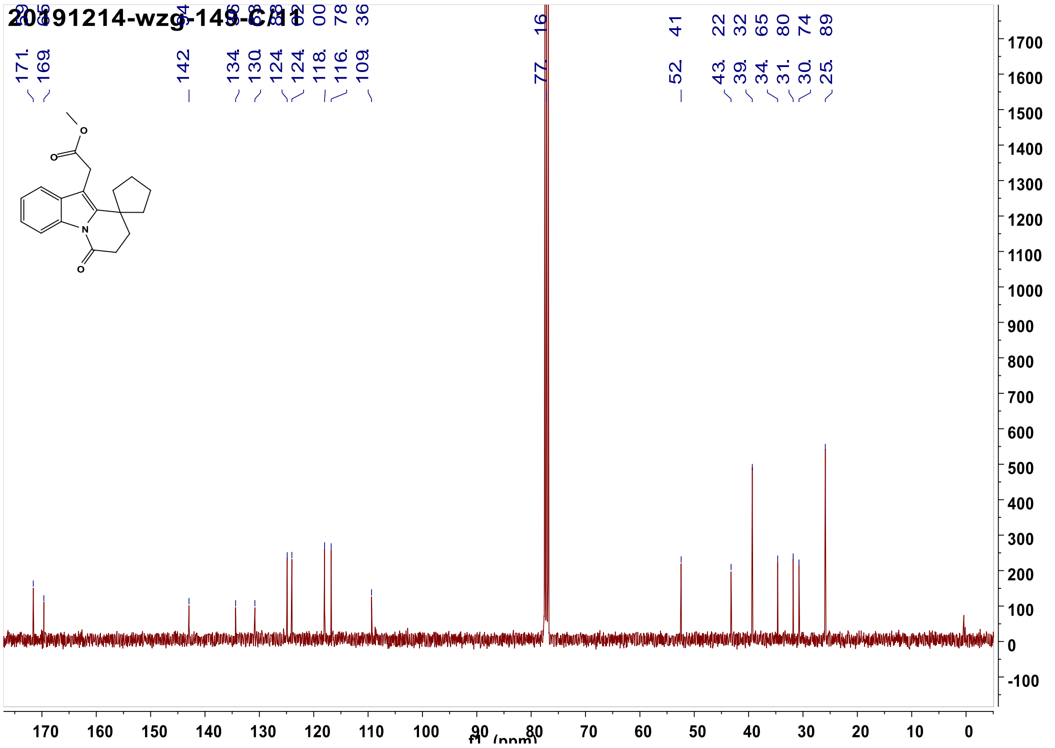

^{13}C NMR spectrum of compound **20k**

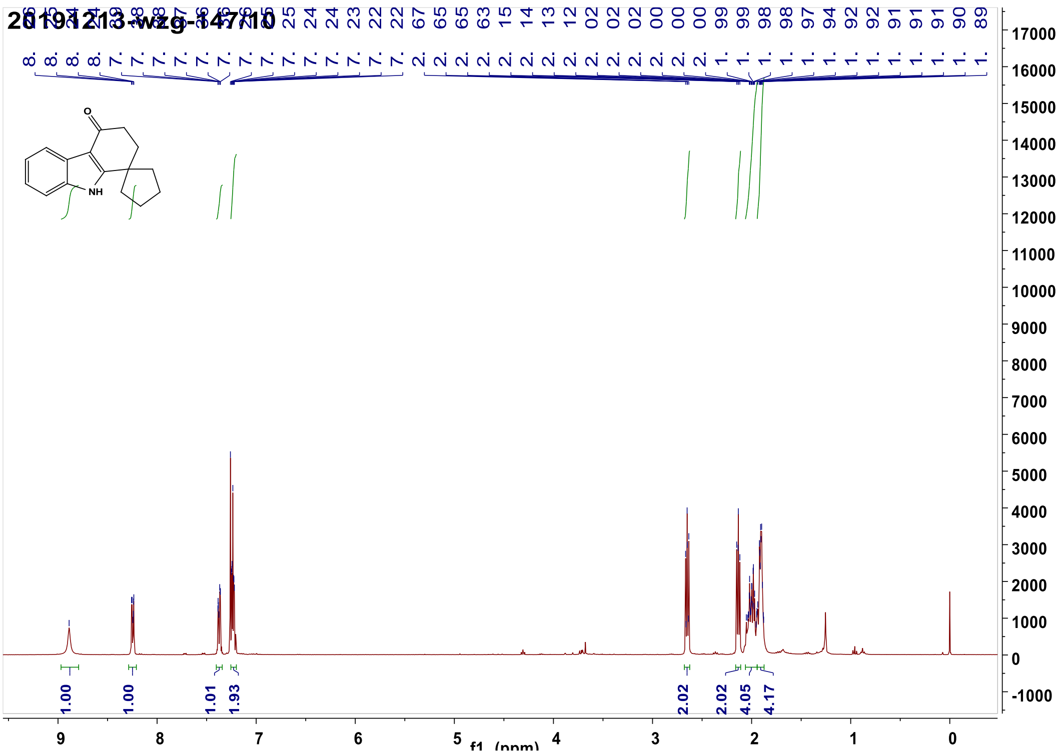

^{1}H NMR spectrum of compound **24**

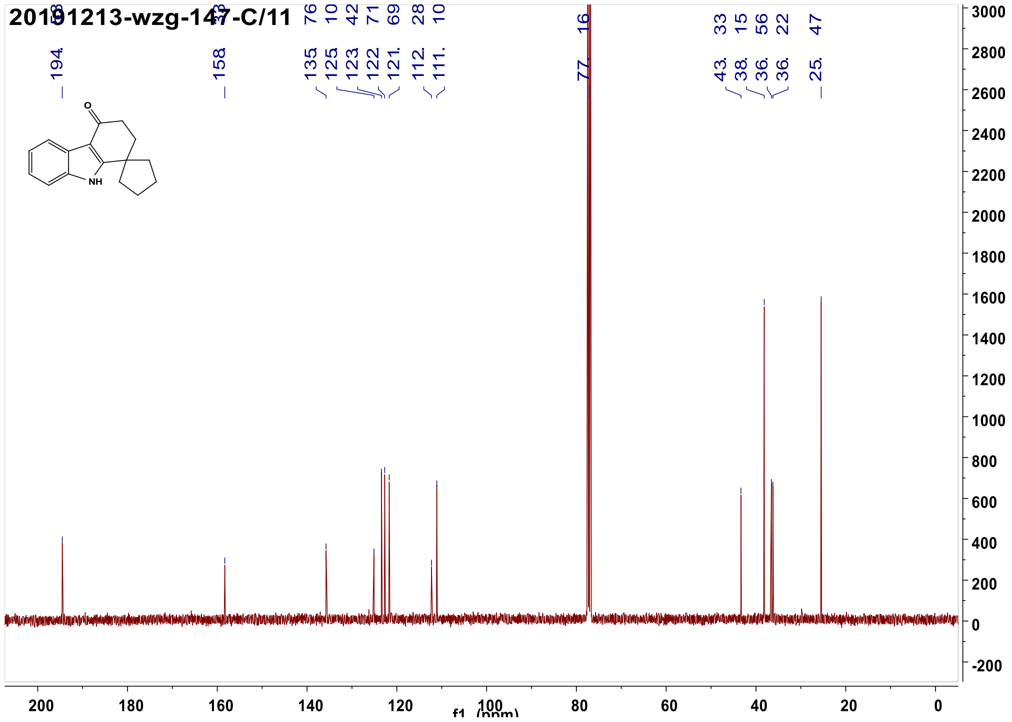

^{13}C NMR spectrum of compound **24**
